# Evidence for an Adaptation of a Phage-Derived Holin/Endolysin System to Toxin Transport in *Clostridioides difficile*

**DOI:** 10.3389/fmicb.2018.02446

**Published:** 2018-10-18

**Authors:** Denise Mehner-Breitfeld, Claudia Rathmann, Thomas Riedel, Ingo Just, Ralf Gerhard, Jörg Overmann, Thomas Brüser

**Affiliations:** ^1^Institute of Microbiology, Leibniz Universität Hannover, Hanover, Germany; ^2^Department of Microbial Ecology and Diversity Research, Leibniz Institute DSMZ-German Collection of Microorganisms and Cell Cultures, Braunschweig, Germany; ^3^German Center for Infection Research, Partner Site Hannover-Braunschweig, Braunschweig, Germany; ^4^Institute of Toxicology, Hannover Medical School, Hanover, Germany

**Keywords:** *Clostridioides difficile*, toxins, holins, endolysins, protein transport

## Abstract

The pathogenicity locus (PaLoc) of *Clostridioides difficile* usually comprises five genes (*tcdR, tcdB, tcdE, tcdA, tcdC*). While the proteins TcdA and TcdB represent the main toxins of this pathogen, TcdR and TcdC are involved in the regulation of their production. TcdE is a holin family protein, members of which are usually involved in the transport of cell wall-degrading enzymes (endolysins) for phage-induced lysis. In the past, TcdE has been shown to contribute to the release of TcdA and TcdB, but it is unclear whether it mediates a specific transport or rather a lysis of cells. TcdE of *C. difficile* strains analyzed so far can be produced in three isoforms that are initiated from distinct N-terminal ATG codons. When produced in *Escherichia coli*, we found that the longest TcdE isoform had a moderate effect on cell growth, whereas the shortest isoform strongly induced lysis. The effect of the longest isoform was inhibitory for cell lysis, implying a regulatory function of the N-terminal 24 residues. We analyzed the PaLoc sequence of 44 *C. difficile* isolates and found that four of these apparently encode only the short TcdE isoforms, and the most closely related holins from *C. difficile* phages only possess one of these initiation codons, indicating that an N-terminal extension of TcdE evolved in *C. difficile*. All PaLoc sequences comprised also a conserved gene encoding a short fragment of an endolysin remnant of a phage holin/endolysin pair. We could produce this peptide, which we named TcdL, and demonstrated by bacterial two-hybrid analysis a self-interaction and an interaction with TcdB that might serve to mediate TcdE-dependent transport.

## Introduction

*Clostridioides difficile* is an opportunistic human pathogen that causes antibiotic-associated diarrhea (Schäffler and Breitrück, 2018). Strains of *C. difficile* usually produce two large protein toxins, TcdA and TcdB, and hypervirulent strains in addition often produce a third toxin, CDT (Shen, 2012). TcdA and TcdB are encoded in a genomic region termed pathogenicity locus (PaLoc) together with the regulatory proteins TcdR and TcdC, and the holin family protein TcdE (Monot et al., 2015). Holins are known to mediate phage lysis by transporting endolysins across the cytoplasmic membrane (Saier and Reddy, 2015). Already in 2001, it had been postulated that TcdE may function as a lytic protein that mediates toxin transport (Tan et al., 2001). Evidence for a direct involvement of TcdE in toxin transport has been obtained for strains that produce high toxin levels (Govind and Dupuy, 2012; Govind et al., 2015) whereas strains that produce low toxin levels release the toxins by TcdE-unrelated lysis that most likely involves a recently discovered peptidoglycan-degrading transglycosylase (Olling et al., 2012; Govind et al., 2015; Wydau-Dematteis et al., 2018). There is not much known about the holin mechanism. Phage holins have been demonstrated to permit the membrane passage of endolysins and to depolarize the cytoplasmic membrane (Catalão et al., 2013), but it is unknown how a holin can mediate the translocation of very large toxins such as TcdA (308 kDa) or TcdB (270 kDa) without non-specific cell lysis. However, there is clear evidence for a TcdE-mediated transport that does not involve cellular lysis (Mukherjee et al., 2002; Govind et al., 2015). *C. difficile* TcdE is known to self-interact (Govind and Dupuy, 2012) and has been shown to trigger lysis of *C. difficile* and *Escherichia coli* (Olling et al., 2012; Govind et al., 2015). In the most recent studies on TcdE, the presence of differential translational start codons came into the focus (Govind and Dupuy, 2012; Olling et al., 2012; Govind et al., 2015), as previous studies on lambda phage holins showed a regulatory function of the co-existence of holin isoforms with close-by but distinct translational starts (Bläsi et al., 1990; Chang et al., 1995; Barenboim et al., 1999). In this well-studied case, only the short isoform is lytic, whereas the isoform extended by two residues is not lytic and believed to serve as antiholin whose production regulates the timing of lysis (Barenboim et al., 1999). The situation with TcdE is complicated by the existence of a third translational start. The three isoforms, named here by their translational start TcdE-M1, TcdE-M25, and TcdE-M27, have been studied with contradictory results. While one study found that the longest construct induced cellular lysis in the absence of shorter isoforms whose production was abolished by mutation of the alternative start codons (Govind et al., 2015), another study found that the longest isoform is non-lytic and attributed the cellular integrity of the tested *C. difficile* strains to the production of this non-lytic TcdE-M1 (Olling et al., 2012). Moreover, the latter study showed that the short isoform was lytic, at that time disregarding the translational start at M25 and focusing the analyses on the two starts at M1 and M27. Although TcdE is required for toxin secretion in strains with high toxin production, it is unknown why this holin is conserved also in the other strains (Govind et al., 2015). TcdE could be shown to be able to transport endolysins (Govind and Dupuy, 2012) and nothing is known about recognitions or interactions that could mediate a toxin specificity.

Here we present data that suggest specific roles for the three translational starts, with TcdE-M27 serving as holin, Tcd-M25 as typical antiholin, and TcdE-M1 as additional non-lytic isoform. The use of M1 provides an N-terminal extension that is inhibitory for lysis even in the presence of abundant short isoforms. This extended N-terminus is not present in the phage holins from which TcdE originated. Genomic analyses indicate that a significant number of *C. difficile* strains still do not contain this extension, suggesting that it evolved during the radiation of the species, most likely to optimize a toxin-secretion related function. A remnant of an endolysin gene that previously has been believed not to be relevant for the PaLoc system is conserved with a strong ribosomal binding site. First experimental data are presented that support the idea that this fragment might mediate interactions with the endolysin-unrelated large toxin TcdB.

## Materials and Methods

### Strains and Growth Conditions

*Escherichia coli* strain ER2566 (NEB, Ipswich, United States) was used for all fractionation studies, and *E. coli* XL1-Blue Mrf’ Kan or Tet (Stratagene, La Jolla, CA, United States), or DH5α were used for cloning. Cells were grown aerobically in LB medium (1% tryptone, 0.5% yeast extract, 0.5% NaCl) at 37°C with the appropriate antibiotics (100 μg/ml ampicillin, 25 μg/ml chloramphenicol, 50 μg/ml kanamycin). 0.5 mM IPTG or 0.1% rhamnose were used to induce P*_lacZ_*- or P*_rhaB_*-dependent protein production at indicated time points. *E. coli* strain BTH101 (Euromedex, Souffelweyersheim, France) was used for bacterial-2-hybrid (B2H) studies. For growth curves, 25-ml cultures were inoculated with OD_600_ of 0.1 and grown aerobically. The OD_600_ was determined in 30-min intervals. For induction of gene expression, 0.01% rhamnose was added at indicated time points. 1% glucose was added for repression of rhamnose-dependent gene expression.

### Genetic Methods and Plasmids

The genes *tcdE* and *tcdL* were rhamnose-inducibly expressed from pBW22 based vectors (Wilms et al., 2001) and C-terminally fused to a hexahistidine-tag. The *tcdE* expression vectors pBW-*tcdE*-M1-H6, pBW-*tcdE_(1-64)_*-H6, pBW-*tcdE_(1-104)_*-H6, pBW-*tcdE_(1-142)_*-H6, pBW-*tcdE*-M25-H6, and pBW-*tcdE*-M27-H6 were cloned via standard methods (see **Table 1** for all primers). A cloned *tcdE* was used as PCR template, which was amplified from chromosomal DNA using the primers SpeI-*tcdE*-F and BamHI-*tcdE*-R, and cloned into the corresponding sites of the vector pCR2.1 TOPO (Thermo Fisher Scientific, Waltham, MA, United States). To construct pBW-*lepB*-H6, the *lepB* gene of *E. coli* was amplified with chromosomal DNA as template, using the primers NdeI-*lepB*-F in combination with BamHI-*lepB*-R, and the fragment was cloned into the corresponding sites of pBW-*tatA*-H6 (Berthelmann et al., 2008). For construction of pBW-*tcdL*-strep, *tcdL* was amplified by PCR in a first step with overlapping primers *tcdL*-synth-NdeI-F and *tcdL*-synth-BamHI-R. The amplified product was used as template for a second step with the primers *tcdL*-NdeI-F and *tcdL*-BamHI-F. PCR products were cut with NdeI/BamHI and cloned in the corresponding sites of pBW-*tatA*-H6 (Berthelmann et al., 2008) or pBW-*tatA*-strep (Mehner et al., 2012), thereby exchanging the fragments. For all these PCR amplifications, Pfu polymerase (Promega, Mannheim, Germany) was used according to the suppliers protocol.

**Table 1:** Primers used in this study^1^.

Cloning of genomic *tcdE*	
SpeI-*tcdE*-F	ACTAGTATGCACAGTAGTTCACC
BamHI-*tcdE*-R	GGATCCCTTTTCACCCTTAGCATTC
Cloning of *tcdE* and its variants	
*tcdE*-M1-NdeI-F	ATTATCATATGCACAGTAGTTCACCTTTTTATATTTC
*tcdE*-BamHI-R	ATATTGGATCCCTTTTCATCCTTAGCATTCATTTC
*tcdE*-S64-BamHI-R	TATATGGATCCAGAATTAAATTTACGACTTTTTATTGC
*tcdE*-I104-BamHI-R	TATATGGATCCGATACAATCTTGTGGTAACATAAATAAAAAG
*tcdE*-P142-BamHI-R	TATATGGATCCAGGTACTGGTAATCCACATAAGCAC
*tcdE*-Met25-NdeI-F	ATTATCATATGAATATGACAATATCTTTTTTATCAG
*tcdE*-Met27-NdeI-F	ATTATCATATGACAATATCTTTTTTATCATGAGC
Cloning of *tcdL*	
*tcdL*-synth-NdeI-F	ATTATCATATGCCAAGAGACACACAAGTATTAAATACATATAATTTCGAAGCAAGTGTTCATTACTATATGGATGACAAGGTAGTATATC
*tcdL*-synth-BamHI-R	ATATTGGATCCATAGATTTTACCAACTGACCATGCACCATCTTTGTGAACCAATGTTTGATATACTACCTTGTCATCCATATAGTAATG
*tcdL*-NdeI-F	ATTATCATATGCCAAGAGACACACAAG
*tcdL*-BamHI-F	ATATTGGATCCATAGATTTTACCAACTG
Cloning of *lepB*	
NdeI-*lepB*-F	TATTACATATGGCGAATATGTTTGCCCTGATTCTGG
BamHI-*lepB*-R	TAATAGGATCCATGGATGCCGCCAATGCGACTTAAGC
Single amino acid exchanges^2^	
*tcdE*_M25/27L_-F	AACTTTATAAATATATGCTCTGATAAAAAAGATATTGTGAGATTGAGAACGCCTCCTAGGTTTATATAAAAAAATATTTTGTTACCATTAG
*tcdE*_ΔRBS_-F	GCTCTGATAAAAAAGATATTGTCATATTCATAACGAGTAATAGGTTTATATAAAAAAATATTTTGTTACCATTAGAAATATAAAAAGGTGA
*tcdE*-M25_M27L_-F	ATATGCTCTGATAAAAAAGATATTGTGAGATTCATATGTATATCTCCTTCTTAAGAATTGTTC
*tcdE*-I151>V-F	GATTAAAGGAAAAAATAGCAGTTTTACTAGATGCAATGACAG
*tcdB*-sil-NdeI-F	CTTAAGTGGCCCTGAAGCGTATGCGGCAGCTTATCAAG
Bacterial-2-hybrid screen	
2H-*tcdE*-BamHI-F	TATATGGATCCCACAGTAGTTCACCTTTTTATATTTC
2H-*tcdE*-KpnI-R	TATATGGTACCCCCTTTTCATCCTTAGCATTC
2H-*tcdB*-BamHI-F	TATATGGATCCGATAAACTTGTTCACTTAAATC
2H-*tcdB*-KpnI-R	TATGGATCCAGTTTAGTTAATAGAAAACAGTTAG
2H-*tcdL*-BamHI-F	TATATGGATCCCCAAGAGACACACAAGTATTAAATAC
2H-*tcdL*-KpnI-R	TATATGGTACCCCATAGATTTTACCAACTGAC

*^*1*^Primer sequences are given in 5′-3′ direction. ^*2*^Corresponding reverse primers covered the same sequence.*

Single amino acid exchanges in TcdE were introduced by QuikChange mutagenesis (Stratagene) of pBW-*tcdE-*M1-H6 or pBW-*tcdE*-M25-H6 vectors and the listed primers (**Table 1**) in conjunction with reverse primers that cover the identical sequence region. To correct the sequence to TcdE of *C. difficile* R20291, I151 was exchanged to V151 in TcdE with the following primer pair *tcdE*-I151>V-F and *tcdE*-I151>V-R using the same protocol.

Site specific mutagenesis of pHIS1522-*tcdB* D286/288N (Wohlan et al., 2014) was used to introduce a silent mutation in *tcdB* at postion A473 with the primer pair *tcdB*-sil-NdeI-F and *tcdB*-sil-NdeI-R, which eliminated an intrinsic NdeI-site, resulting in the vector pHIS1522-*tcdB* D286/288N silent NdeI. To analyze protein–protein interactions by the B2H screen, *tcdE*-M1, *tcdL*, and *tcdB* were amplified with the primer pairs 2H-*tcdE*-BamHI-F/2H-*tcdE*-KpnI-R, 2H-*tcdB*-BamHI-F/2H-*tcdB*-KpnI-R, and 2H-*tcdL*-BamHI-F/2H-*tcdL*-KpnI-R, and cloned into the corresponding sites of pU-strep-*pspA*(25–144)-T18, pUT18C-*pspA*(25–144)-strep, pK-strep-*pspA*(25–144)-T25, and pKT25-*pspA*(25–144)-strep (Heidrich and Brüser, 2018). Single amino acid exchanges in TcdE for I151V were done as described above. All constructs were verified via restriction analysis and sequencing. The plasmid pLysS was obtained from Promega.

### Biochemical Methods

SDS–PAGE and subsequent Western blotting were carried out by standard procedures (Laemmli, 1970; Towbin et al., 1979). BN-PAGE was performed with 5 to 13.5% gradient gels, and samples were prepared as described previously (Behrendt and Brüser, 2014). Solubilisation of membrane proteins was achieved by adding 1% (w/v) Digitonin. For small proteins, Schägger gels (16% T, 6 M urea) were performed as described elsewhere (Schägger, 2006). For detection of hexahistidine-tagged proteins, Western blots were developed employing specific mouse monoclonal His-tag antibodies (1:1 mixture of penta-His/tetra-His; Qiagen, Venlo, Netherlands) in combination with the goat polyclonal anti mouse IgG secondary antibodies (Roth, Karlsruhe, Germany) coupled to horseradish peroxidase for enhanced chemoluminescence (ECL). Strep-tagged proteins were detected using Strep-Tactin alkaline phosphatase conjugate (IBA, Göttingen, Germany). For this, Western blots were washed once with 100 mM Tris pH 9.5, 100 mM NaCl, 50 mM MgCl_2_, and incubated in the same buffer supplied with 0.22 M NBT in DMF and 0.16 M BCIP in DMF for staining. Images of Western blots were acquired utilizing the Intas Advanced Imager (INTAS, Göttingen, Germany).

For subcellular fractionations, cells were aerobically grown at 37°C in the presence of the appropriate antibiotics. If protein production was rhamnose-dependent, rhamnose was added to a final concentration of 0.1 or 0.01% (v/v), and cultivation proceeded for 1 h. Cells were harvested via centrifugation at 4.500 × *g* for 10 min at 4°C. Cell densities of corresponding cultures were normalized prior to the centrifugation step. Cell pellets were suspended in 50 mM Tris HCl pH 8.0, 250 mM NaCl, and after adding DNase I and 1 mM PMSF, and cells were homogenized by ultrasonication or French press (two passages, 800 p.s.i.). Cell debris was separated by centrifugation at 14.000 × *g* for 10 min at 4°C. Fractions of cell debris, supernatant (crude extract) or membranes were analyzed by SDS–PAGE and Western blotting.

In the course of the B2H analyses in *E. coli* strain BTH101, LacZ activities were determined using the classic activity assay by Miller (1972). In brief, overnight cultures were diluted 1:25 and cells were grown aerobically in the presence 0.5 mM IPTG at 30°C for 4 h. Cells from 0.5 ml of culture were harvested and resuspended in 2 ml Z-buffer (60 mM Na_2_HPO_4_, 40 mM NaH_2_PO_4_, 10 mM KCl, 1 mM MgSO_4_), and the optical density at 600 nm was determined. Cells from 1 ml of this dilution were permeabilized by adding two drops chloroform and one drop 0.1% SDS. After vortexing for 10 s, the reaction was started at 28°C by addition of 0.2 ml ONPG (4 mg/ml). The reaction was terminated after appropriate incubation times by the addition of 0.5 ml of the 1 M Na_2_CO_3_, the cells were sedimented (13.000 rpm, 5 min, RT), and the absorption at 420 nm was recorded for calculation of the activity as described (Miller, 1972).

### Genomic Sequence Extraction in Between the Genes *tcdB* and *tcdA*

*Clostridioides difficile* genomes of 38 strains were sequenced using SMRT and Illumina technology, assembled and annotated as described previously (Riedel et al., 2015a,b, 2017; Dannheim et al., 2017). From these genomes and from six additional *C. difficile* reference genomes (M68, CF5, 2007855, BI1, R20291, and M120) downloaded from GenBank^1^, the nucleotide sequence regions in between the two genes *tcdB* and *tcdA* were extracted (**Supplementary Data**) and analyzed as described.

## Results

### Evidence for a Suppression of Translational Initiation at M1

**FIGURE 1:**
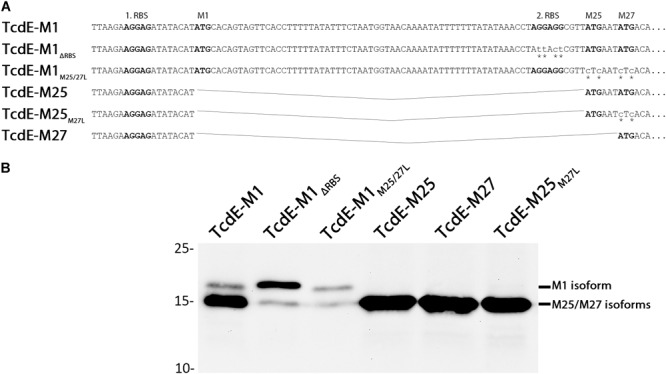
Analysis of the translation of the three distinct TcdE isoforms in *E. coli*. **(A)** Constructs for the production of TcdE isoforms that initiate translation at M1, M25, or M27, with or without an inactivated second ribosomal binding site (ΔRBS) or translational start at M25 or M27, as indicated. Mutated bases are labeled by asterisks. Ribosomal binding sites and translational starts are in bold letters. **(B)** SDS–PAGE/Western blot detection of M1 and M25/M27 isoforms produced by the *E. coli* strains ER2566 pBW-*tcdE-M1*-H6, pBW-*tcdE*-M1_ΔRBS_-H6, pBW-*tcdE*-M1_M25/27L_-H6, pBW-*tcdE*-M25-H6, pBW-*tcdE*-M27-H6, and pBW-*tcdE*-M25_M27L_-H6, respectively, using specific His-tag antibodies and ECL reaction. Induction was with 0.1% rhamnose. Molecular weight markers (in kDa) are indicated on the left.

We analyzed the three TcdE isoforms Tcd-M1, Tcd-M25, and Tcd-M27 in the heterologous *E. coli* system that has been previously demonstrated to be functional with respect to lysis induction (Olling et al., 2012). In addition, we generated TcdE-M1 variants in which the other two translational starts were abolished either by M25L/M27L exchanges (TcdE-M1_M25/27L_) or by mutation of the second Shine-Dalgarno sequence that serves to initiate translation from M25 and M27 on (TcdE-M1_ΔRBS_). We also generated a TcdE-M25_M27L_ construct that can only initialize at M25 due to a M27L mutation (**Figure 1A**). To ensure comparable and regulated expression, we used an identical Shine-Dalgarno sequence and the rhamnose-inducible P*_rhaB_* promoter for all constructs. A similar approach was taken in one of the previous studies that used an isopropyl-β-D-thiogalactopyranoside inducible T7 promoter, but in that study the TcdE-M25 construct was not included and the strong T7 promoter might have masked differential effects of the constructs (Olling et al., 2012). We observed primarily a production of the short isoform(s) with the TcdE-M1 construct, and only little full-length TcdE-M1 was detected (**Figure 1B**). Importantly, the removal of the internal initiation starts at M25/M27 in the construct TcdE-M1_M25/27L_ resulted in a marked decrease of TcdE abundance selectively for the shorter isoforms, indicating that M25 and M27 are predominantly used as translational starts, even in the presence of an M1 start codon in conjunction with a very good Shine-Dalgarno sequence. As the TcdE-M25, TcdE-M25_M27L_, and TcdE-M27 constructs were highly abundant when produced with the same Shine-Dalgarno sequence (**Figure 1B**, compare lanes 4,5,6 with lanes 2,3) it is obvious that the translational start at M1 is suppressed in TcdE-M1 as well as in TcdE-M1_M25/27L_ constructs, and the translational start at M25/M27 is therefore not responsible for the suppression of translational start at M1. The lower abundance cannot be explained by degradation, as the amount of full-length TcdE initiated at M1 was only slightly reduced. Nevertheless, the formation of small amounts of TcdE with the size of the small isoforms especially in the TcdE-M1_ΔRBS_ construct indicates some degradation to about the size of the M25/M27 isoforms, which therefore likely represent a protease-resistant core. Taken together, these initial data indicated that the N-terminal 24 residues are only made in small amounts and rather represent some kind of extension of the TcdE core that begins around M25/M27. This suppression of translation at M1 likely results from an mRNA secondary structure around the M1 initiation codon and its Shine-Dalgarno sequence, which is evidenced by the fact that the TcdE-M1_ΔRBS_ construct results in significantly more “full-length” TcdE-M1 than the TcdE-M1_M25/27L_ construct (compare M1 bands in **Figure 1B**, lanes 2 and 3) and this is supported by the predicted mRNA secondary structures in that region. We used the software RNAstructure (Reuter and Mathews, 2010) to predict the secondary structure formation in a 130 nucleotides region (from -24 to +106) around the first initiation site and found that, in full agreement with the hypothesis, the TcdE-M1 construct formed a quite stable secondary structure including the Shine-Dalgarno sequence, and the stability of secondary structures around that region decreased as a consequence of the exchanges in the TcdE-M1_ΔRBS_ construct due to a competing hairpin (**Supplementary Figure S1**). This explains the observed increase of translation initiation at M1 in the TcdE-M1_ΔRBS_ relative to TcdE-M1 and TcdE-M1_M25/27L_.

### TcdE-M27 Is the Lytic Isoform, TcdE-M25 Is Hardly Active, and TcdE-M1 Inhibits Lysis

**FIGURE 2:**
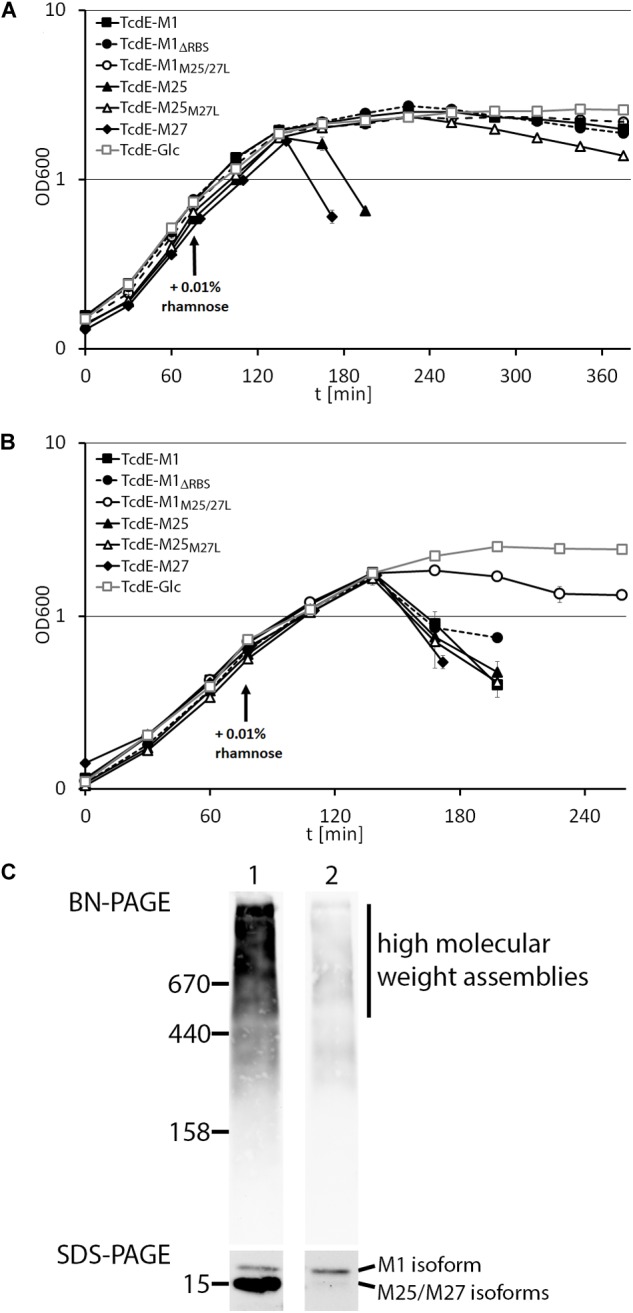
TcdE-dependent lysis can be triggered by endolysins. Growth curves of *E. coli* strain ER2566 with recombinant rhamnose-induced production of either TcdE-M1, TcdE-M1_ΔRBS_, TcdE-M1_M25/27L_, TcdE-M25, TcdE-M25_M27L_, and TcdE-M27. As negative control, glucose instead of rhamnose was added to the strain containing the TcdE-M1 construct. Growth was without **(A)** or with **(B)** constitutive production of LysS from pLysS. Induction was with 0.01% rhamnose at the indicated time point. Standard deviations were calculated from three independent cultures. **(C)** BN-PAGE analysis of TcdE associations formed with the TcdE-M1 construct (1) and with the TcdE-M1_M25/27L_ construct (2), showing the presence of large TcdE associations using the *E. coli* strains ER2566 pBW-*tcdE-M1*-H6 and pBW-*tcdE*-M1_M25/27L_-H6, respectively. SDS–PAGE analysis of the TcdE isoforms, showing that the strain with the TcdE-M1 construct contains also highly abundant smaller isoforms, whereas the TcdE-M1_M25/27L_ construct contains very low amounts of smaller isoforms. Western blot detection using His-tag antibodies and ECL reaction. Molecular weight markers (in kDa) are indicated on the left.

We then examined the effect of the above described TcdE constructs on growth (**Figure 2A**). Notably, only TcdE-M25 and TcdE-M27 constructs that allowed a translational initiation at M27 resulted in immediate cell lysis. The construct TcdE-M25_M27L_ showed a very low efficient lysis effect, which may be due to N-terminal degradation to TcdE-M27 over time, indicating that TcdE-M25 is much less lytic than TcdE-M27. Also TcdE-M1, TcdE-M1_M25/27L_ and TcdE-M1_ΔRBS_ had only weak and retarded effects. There was no lysis when expression from the rhamnose promoter was suppressed by glucose in the medium. Taken together, these data clearly show that initiation at M27 triggers cell lysis in the absence of endolysins, whereas initiation at M1 or M25 does not. Already low amounts of TcdE-M1 inhibit lysis in the presence of large amounts of the small TcdE isoforms (**Figure 1**), suggesting that the isoform TcdE-M1 is really inhibitory for TcdE-M27 mediated cell lysis. As lysis is not delayed in a strain producing TcdE-M25 together with TcdE-M27 (construct TcdE-M25), TcdE-M25 appears to be not as inhibitory as TcdE-M1 with respect to endolysin-independent TcdE-triggered lysis, but we cannot differentiate the abundances of TcdE-M25 and TcdE-M27 by these Western blots.

### TcdE-Dependent Lysis Can Be Triggered by the Transport of Endolysins and Requires the TcdE C-Terminus

It has been already demonstrated that TcdE can cause cell lysis in the presence of Lambda endolysin (Govind and Dupuy, 2012; Monot et al., 2015). We thus analyzed the potential effect of an endolysin on TcdE-dependent cell lysis. We constitutively produced T7 lysozyme with pLysS, which is a vector compatible with the rhamnose-induced vector system we used for expression of the *tcdE* variant genes. Importantly, while TcdE-M25 and TcdE-M27 caused the expected lysis that had already been observed in the absence of endolysins, TcdE-M1 showed a much enhanced cell lysis, indicating that this construct was able to transport T7 lysozyme across the cytoplasmic membrane (**Figure 2B**). As negative control, we suppressed rhamnose-dependent gene expression by glucose in the medium. As TcdE-dependent transport of lambda endolysin R has been previously demonstrated (Govind and Dupuy, 2012), which is unrelated to T7 lysozyme in sequence, it can be concluded that TcdE is able to mediate the escape of a wide range of endolysins across the cytoplasmic membrane. As expected for a transport-mediating holin, we found by BN-PAGE analysis that TcdE formed large associations of multiple protomers (**Figure 2C**). Notably, the TcdE-M1 isoform was already able to form large associations, and the smaller isoforms clearly contributed to the formation of associations with masses higher than 500 kDa.

**FIGURE 3:**
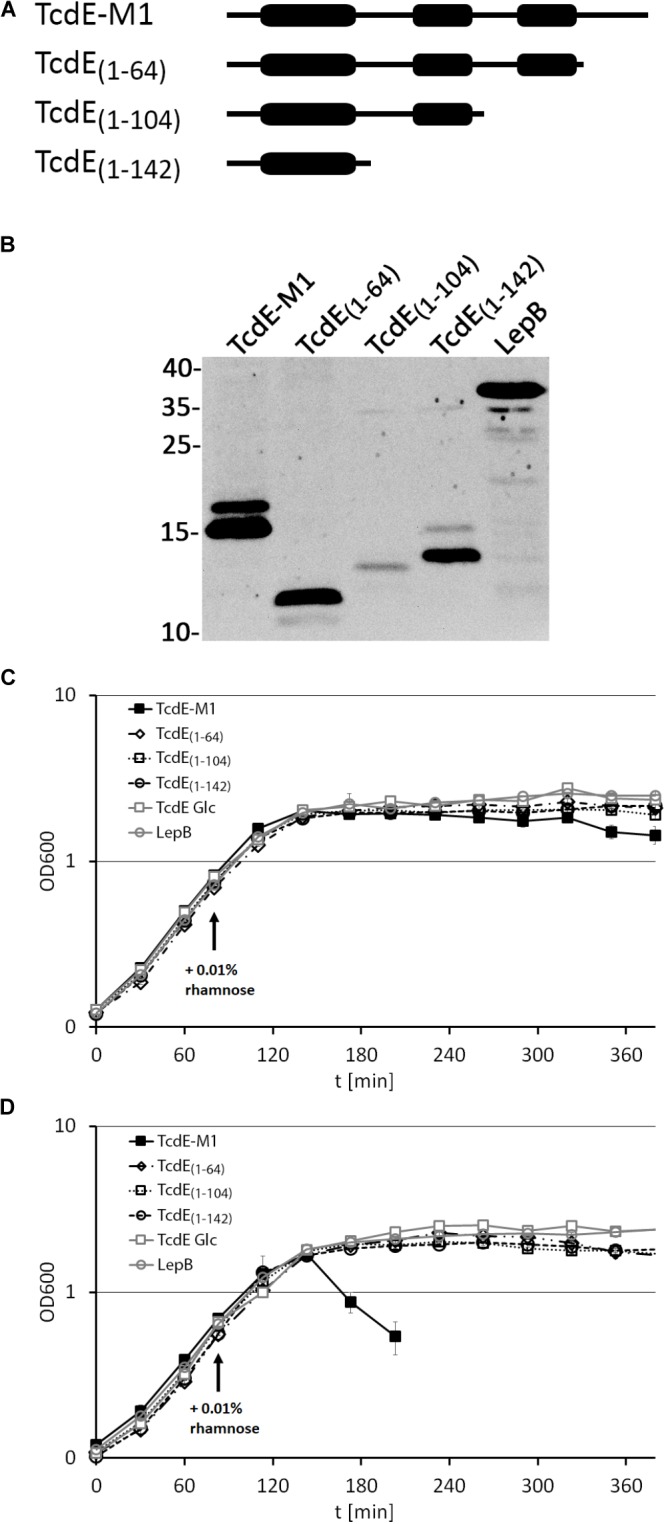
C-terminal truncation abolishes the lytic effect of TcdE. **(A)** Scheme of the analyzed constructs. The thick regions represent potential *trans*-membrane domains. **(B)** Western blot analysis of the constructs produced by the *E. coli* strains ER2566 pBW-*tcdE-M1*-H6, pBW-*tcdE_(1-64)_*-H6, pBW-*tcdE_(1-104)_*-H6, pBW-*tcdE_(1-142)_*-H6, and pBW-*lepB*-H6. Western blot detection using His-tag antibodies and ECL reaction. Molecular weight markers (in kDa) are indicated on the left. The unrelated membrane protein LepB was used as negative control. **(C)** Effect of the indicated constructs on growth. “Glc” refers to a culture in which rhamnose-dependent expression of the full-length *tcdE* construct (TcdE-M1) was suppressed by glucose in the medium. **(D)** The same experiment as in **(C)** but with strains producing constitutively LysS from pLysS. Note that only the full-length TcdE-M1 construct is capable of mediating lysis, indicating LysS transport into the periplasm.

We then examined whether the C-terminal region of TcdE is important for the lytic effect and analyzed TcdE variants truncated behind position P142, I104, or S64 (numbering starting at M1), which correspond to the C-terminal ends of the predicted three *trans*-membrane domains (**Figure 3A**). All constructs were produced to comparable levels as judged by SDS–PAGE/Western blot, with the exception of TcdE_1-104_ that was significantly lower abundant (**Figure 3B**). In the absence of the endolysin LysS, none of these TcdE variants caused significant cell lysis, which agrees with the previous conclusion that the N-terminal extension renders TcdE lytically inactive (**Figure 3C**). In the presence of the endolysin, the TcdE-M1 construct was lytically active as described above, but none of the truncated variants could cause cellular lysis, suggesting that the C-terminal domain that follows the third trans-membrane domain is functionally important (**Figure 3D**). As negative control, the production of the unrelated membrane protein LepB had no lytic effect.

### The N-Terminal Region of TcdE Evolved Within the Clostridia

**FIGURE 4:**
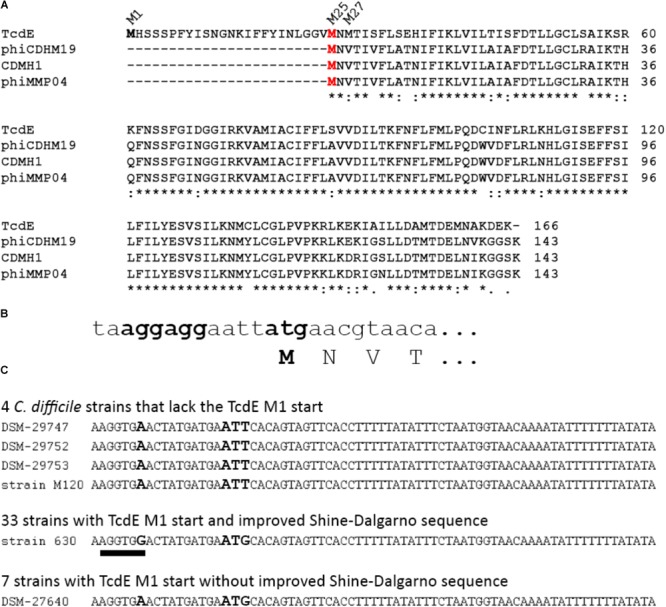
The N-terminal extension of TcdE evolved within clostridia. **(A)** Comparison of the N-terminal sequence of TcdE with that of the three most closely related TcdE homologs from *C. difficile* phages. Note that the M1 and M27 isoforms are lacking in the phages, and only M25 (red) is conserved in all cases. “^∗^” indicates identical positions, “^:^” positions with highly similar exchanges, “^.^” positions with weakly similar exchanges; **(B)** DNA sequence of the translational start region in all three phage *tcdE* homologs. Note that the Val codon that corresponds to the M27 in TcdE is not the rare initiation codon GTG, but rather the valine codon GTA. **(C)** DNA sequence comparison that shows the lack of the standard M1 translational start for TcdE in four of the analyzed *C. difficile* strains, and a less efficient ribosomal binding site in these four cases and in further seven strains, with DSM 27640 as example. The corresponding sequence of strain 630 is given as example for the 33 analyzed strains that possess a canonical M1 translational start with a good Shine-Dalgarno sequence. The improved Shine-Dalgarno sequence is underlined. Bold letters indicate the variable position in the Shine-Dalgarno sequence and the position of the M1 start codon.

As initiation at M1 was low efficient, and as the stability of the M25 and M27 isoforms suggested that these shorter isoforms constitute some protease-resistant core, we examined the existence of the differential translational starts in the most closely related phage holins, which are the holins of phiCDHM19, CDMH1, and phiMMP04, all of which are phages of *C. difficile*. These phage holins are highly similar among each other (96% identity) and are 79% identical to TcdE-M25. Importantly, all three phage holins clearly lack the N-terminal extension found in TcdE-M1, and all of them are most likely produced from only one translational start, which corresponds to M25 in TcdE, as a Val_GTA_ codon is in the phage holins at the position of the M27_ATG_ codon (**Figures 4A,B**). However, we cannot exclude that this GUA codon can be used by *C. difficile* as rare translational start. All known natural non-AUG start codons differ from AUG only by single bases (Diaz de Arce et al., 2018). For *E. coli recJ* it has been reported that a translational start mutated to GUA could be used with low efficiency, which to our knowledge is the only demonstrated case of a functional translational start at GUA (Haggerty and Lovett, 1997). Having clarified that the translational start at M1 in TcdE is not found in the most closely related phages, we addressed the question whether TcdE proteins lacking this extension exist in *C. difficile* strains. For that purpose, *tcdE* genes of 44 distinct *C. difficile* isolates were analyzed (**Figure 4C**). Interestingly, in four of these strains, an ATT codon was at the position of the commonly found M1 ATG start codon. ATT is not known as translational start in *C. difficile*, but in *E. coli*, ATT has been found as rare non-canonical translational start codon in three out of >4200 protein-coding genes (*ymcF, ymfQ, infC*). It thus cannot be excluded that some translation can initiate at this codon also in *C. difficile*. The *tcdE* genes of all 44 strains possess the canonical M25 and M27 translational starts (see **Supplementary Data**). In 11 out of the 44 strains, the Shine-Dalgarno sequence was rather bad due an A/C mismatch, and among these were the four strains that encoded no M1 translational start (**Figure 4C**). Together, the sequence data indicate that an introduction and optimization of the M1 translational start codon was followed by an improvement of the corresponding Shine-Dalgarno sequence. Most importantly, an initiation at M1 evolved in *C. difficile*, and not in phages, and therefore the N-terminal 24 residues extension likely relates to the function that TcdE has in *C. difficile*.

### A Remnant of a Phage Endolysin, TcdL, May Mediate Toxin Interactions With the System

TcdE is known to be a holin that originated from lytic phage holin/endolysin systems (Monot et al., 2015), and our above analyses have indicated the closest phage homologs of TcdE. We wondered, whether a remnant of the endolysin could play a role. Such a remnant has been identified (gene ID CD630_06620) and regarded as pseudogene (Monot et al., 2015). However, a fragment of the endolysin could in principle be produced that does not need to have hydrolytic activity if it serves to mediate toxin transport functions rather than cell lysis in *C. difficile*. The endolysin fragment is unrelated to the endolysins from the three phages that contain holins closely related to TcdE and corresponds to a fragment of an endolysin found in the *C. difficile* phage phiCD481-1. The original endolysin gene is heavily mutated in the second half, whereas there are still very high sequence identities on DNA level in the first half (**Supplementary Figure S2**). A one basepair deletion has generated an early stop codon, and the CD630_06620 open reading frame starts with an ATG codon that is not in frame of the orginal endolysin. The sequence of TcdL is brought into the correct frame by a frameshift after five codons, which already argues for some potential functionality of this gene. TcdL is a peptide of 53 amino acids (6.3 kDa). A Shine-Dalgarno sequence is conserved in all 44 isolate sequences that we have analyzed. In fact, there was only one A>G point mutation in 13 out of 44 *tcdL* sequences, and this point mutation included the four strains that also had not the M1 translational start in TcdE. The Shine-Dalgarno sequence differs from the phage endolysin-encoding sequence at one position, which potentially improves ribosome binding (AAGG>GAGG, see **Supplementary Figure S2**).

**FIGURE 5:**
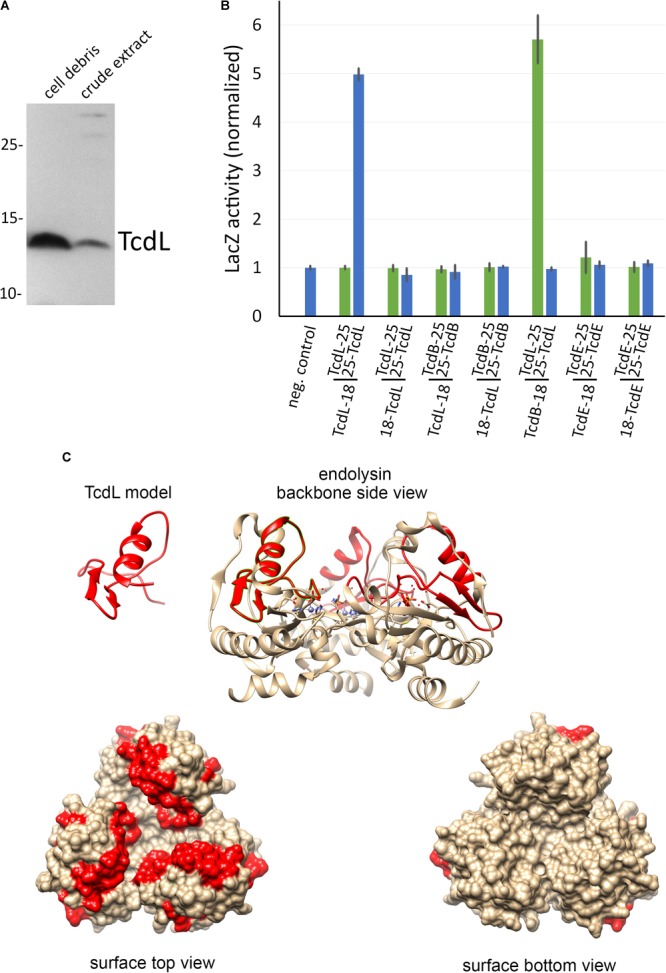
Analyses of TcdL. **(A)** SDS–PAGE/Western blot detection of recombinantly produced Strep-tagged TcdL in cell debris and crude extract from the *E. coli* strain ER2566 pBW-*tcdL*-strep using Strep-Tactin alkaline phosphatase conjugate and NBT/BCIP. **(B)** Bacterial-2-hybrid analysis of TcdL, TcdB, and TcdE interactions, using LacZ activity assays. Data were normalized to the negative control values. Adenylate cyclase domains T18 or T25 were fused N-terminally or C-terminally to the proteins as indicated below the diagram. Note that the N-terminal TcdL-fusion to T18 (= TcdL-18) interacted with the C-terminal TcdL-fusion to T25 (= 25-TcdL), and the N-terminal TcdB-fusion to T18 (= TcdB-18) interacted with the N-terminal TcdL-fusion to T25 (= TcdL-25). **(C)** Homology-modeled TcdL structure and the corresponding region in the trimeric prophage endolysin (PDB 1YB0). Homology modeling was done using Swiss-Model (Waterhouse et al., 2018), and the images were created using CHIMERA (Pettersen et al., 2004).

To analyze whether the peptide can be stably produced, we cloned it into the rhamnose-inducible expression system and detected it by Western blot in subcellular fractions. The peptide was detectable but largely formed inclusion bodies (**Figure 5A**). We term this gene product TcdL, which relates to its lysis protein origin. We then examined potential interactions of TcdL with TcdL, TcdE, and TcdB by use of a bacterial two hybrid system that is based on the reconstitution of adenylate cyclase activity from enzyme fragments that are brought in close proximity by interactions of fused proteins (Karimova et al., 1998). Notably, TcdL interacted with itself and with TcdB (**Figure 5B**). We did not detect any TcdE interaction. The TcdB interaction for the first time related TcdL directly to the toxins and possibly toxin transport.

## Discussion

This study reveals two main aspects with relevance for the field: (1) TcdE-M25 and TcdE-M27 behave similar to the well-studied Lambda S holin that has two translational starts, the first resulting in an antiholin and the second resulting in the lytically active holin (Chang et al., 1995). In contrast to phage holins, an additional isoform with an N-terminal extension evolved in *C. difficile* which inhibits lysis in the absence of transported substrates, i.e., in the absence of an endolysin (**Figure 4**). Interestingly, the extended isoform did either not inhibit transport of LysS, or the presence of LysS abrogated the inhibition of the lytic effect of TcdE (**Figure 2**). The finding that the N-terminal extension evolved most likely in PaLoc-encoded TcdE suggests that it is somehow related to toxins and their secretion. (2) There exists a remnant of a phage endolysin, TcdL, that might play a role in toxin transport as it is definitively inactive in its hydrolytic function but still may mediate interactions. The small protein *per se* is not soluble, which is expected for a fragment of a globular protein. However, interaction studies support an interaction of TcdL with the toxin TcdB (**Figure 5B**). It therefore is possible that the conserved fragment mediates transport processes that holins usually carry out with endolysins. Based on the known structure of the closely related *Bacillus anthracis* prophage endolysin PlyL (PDB 1YB0), we modeled the TcdL structure and analyzed the position of the corresponding region in the trimeric endolysin (**Figure 5C**). Interestingly, TcdL is exposed to one face of the trimer and likely mediates the interaction of the endolysin with the associated phage holin. As the catalytic endolysin domain is absent in TcdL, it can be speculated that the TcdL-interacting toxin substitutes this domain, which could be the basis for a holin interaction that results in transport. However, such aspects are difficult to address and need thorough further analyses, including cross-linking approaches and biochemical transport analyses that so far could not be established for any holin. Attempts to transport TcdB into the periplasm of *E. coli* failed so far but the direct detection of selective transport would be a key to clarify these points.

Interesting is the aspect of the differential initiation at M1, M25, or M27 in *C. difficile* in comparison to *E. coli* as heterologous host. Some strains are reported to produce exclusively TcdE-M1, although they have unaltered initiation sites at M25 and M27. This is physiologically relevant, as TcdE-M1 has been shown not to cause cell lysis in several tested strains (Olling et al., 2012), and our data confirm this. However, we also observed that TcdE-M1 can in principle mediate cell lysis in strains that produce endolysins, which can explain results obtained by another study (Govind et al., 2015). The fact that, in *E. coli*, translation initiation at M1 was strongly inhibited, and the observation that point mutations within the second Shine-Dalgarno sequence could partially release this inhibition (**Figure 1B**) argues for the involvement of RNA secondary structures. Such structures have been shown to be important for the differential initiation at the two translational starts of the lambda holin S (Chang et al., 1995), and similarly have been suggested to play a role for the initiation sites at M25 and M27 of TcdE (Govind et al., 2015). However, secondary structures have so far not been considered for the initiation of TcdE at M1. Importantly, in the natural system, we found that there exists a RBS-protecting hairpin at M1 that is predicted to be very stable, implying that without additional regulatory components there would not be significant initiation at this translational start site (**Supplementary Figure S1**). The fact that it has been observed that *C. difficile* initiates in several tested strains only at this M1 site suggests that most likely a sRNA is involved that promotes initiation at M1, thereby suppressing toxin-substrate-independent lysis. This fits to the finding that TcdE is not responsible for a lytic release of the toxins in strains that release only low amounts of toxins (Govind et al., 2015). In agreement with these and our data, it has been recently demonstrated that a TcdE-independent lytic toxin release exists as an alternative pathway in parallel to the TcdE-dependent non-lytic pathway (Wydau-Dematteis et al., 2018). The lytic pathway depends on a novel transglycosylase that is active on specific media, especially in the stationary growth phase (Wydau-Dematteis et al., 2018). In principle, TcdE could also contribute to lysis when initiation at M1 would be suppressed by the secondary structure, which would induce a switch to lysis. However, initiation at M1 appears to be the rule, and this could be achieved by a regulatory sRNA. A single potential regulatory sRNA has been identified in the PaLoc, which is encoded at position 785940–786181 of the *C. difficile* 630 reference genome (Soutourina et al., 2013). We tested potential influences of this sRNA on secondary structures around M1 using the bimolecular interaction analysis tool of RNAstructure (Reuter and Mathews, 2010) and found no evidence for an influence of this sRNA on the M1 hairpin, which could base-pair only at distant positions. It thus remains unknown why initiation at M1 is so efficient in several *C. difficile* strains (Olling et al., 2012), albeit a very stable hairpin can be formed that is expected to efficiently suppress initiation. We propose that additional factors must be involved in the regulated use of this translational start.

## Author Contributions

DM-B and CR carried out all experimental work except genomic sequencing. TR and JO carried out genomic analyses. IJ and RG provided non-toxic *tcdB* variants and *tcdE*. TB and DM-B designed the experiments. TB supervised the study and wrote the initial manuscript. All authors contributed to the final manuscript.

## Conflict of Interest Statement

The authors declare that the research was conducted in the absence of any commercial or financial relationships that could be construed as a potential conflict of interest.

## Footnotes

1 https://www.ncbi.nlm.nih.gov/

## References

[B1] Barenboim M. Chang C. Y. dib Hajj F. Young R. (1999). Characterization of the dual start motif of a class II holin gene. *Mol. Microbiol.* 32 715–727. 10.1046/j.1365-2958.1999.01385.x 10361276

[B2] Behrendt J. Brüser T. (2014). The TatBC complex of the Tat protein translocase in *Escherichia coli* and its transition to the substrate-bound TatABC complex. *Biochemistry* 53 2344–2354. 10.1021/bi500169s 24654648

[B3] Berthelmann F. Mehner D. Richter S. Lindenstrauss U. Lünsdorf H. Hause G. (2008). Recombinant expression of tatABC and tatAC results in the formation of interacting cytoplasmic TatA tubes in *Escherichia coli*. *J. Biol. Chem.* 283 25281–25289. 10.1074/jbc.M707757200 18644791

[B4] Bläsi U. Chang C. Y. Zagotta M. T. Nam K. B. Young R. (1990). The lethal lambda S gene encodes its own inhibitor. *EMBO J.* 9 981–989. 10.1002/j.1460-2075.1990.tb08200.x 2138979 PMC551767

[B5] Catalão M. J. Gil F. Moniz-Pereira J. São-José C. Pimentel M. (2013). Diversity in bacterial lysis systems: Bacteriophages show the way. *FEMS Microbiol. Rev.* 37 554–571. 10.1111/1574-6976.12006 23043507

[B6] Chang C. Y. Nam K. Young R. (1995). S gene expression and the timing of lysis by bacteriophage lambda. *J. Bacteriol.* 177 3283–3294. 10.1128/jb.177.11.3283-3294.1995 7768829 PMC177022

[B7] Dannheim H. Riedel T. Neumann-Schaal M. Bunk B. Schober I. Spröer C. (2017). Manual curation and reannotation of the genomes of *Clostridium difficile* 630Δerm and *C. difficile* 630. *J. Med. Microbiol.* 66 286–293. 10.1099/jmm.0.000427 28357980

[B8] Diaz de Arce A. J. Noderer W. L. Wang C. L. (2018). Complete motif analysis of sequence requirements for translation initiation at non-AUG start codons. *Nucleic Acids Res.* 46 985–994. 10.1093/nar/gkx1114 29228265 PMC5778536

[B9] Govind R. Dupuy B. (2012). Secretion of *Clostridium difficile* toxins A and B requires the holin-like protein TcdE. *PLoS Pathog.* 8:e1002727. 10.1371/journal.ppat.1002727 22685398 PMC3369941

[B10] Govind R. Fitzwater L. Nichols R. (2015). Observations on the role of TcdE isoforms in *Clostridium difficile* toxin secretion. *J. Bacteriol.* 197 2600–2609. 10.1128/JB.00224-15 26013487 PMC4518823

[B11] Haggerty T. J. Lovett S. T. (1997). IF3-mediated suppression of a GUA initiation codon mutation in the recJ gene of *Escherichia coli*. *J. Bacteriol.* 179 6705–6713. 10.1128/jb.179.21.6705-6713.1997 9352920 PMC179599

[B12] Heidrich E. S. Brüser T. (2018). Evidence for a second regulatory binding site on PspF that is occupied by the C-terminal domain of PspA. *PLoS One* 13:e0198564. 10.1371/journal.pone.0198564 29906279 PMC6003685

[B13] Karimova G. Pidoux J. Ullmann A. Ladant D. (1998). A bacterial two-hybrid system based on a reconstituted signal transduction pathway. *Proc. Natl. Acad. Sci. U.S.A.* 95 5752–5756. 10.1073/pnas.95.10.5752 9576956 PMC20451

[B14] Laemmli U. K. (1970). Cleavage of structural proteins during the assembly of the head of bacteriophage T4. *Nature* 227 680–685. 10.1038/227680a0 5432063

[B15] Mehner D. Osadnik H. Lünsdorf H. Brüser T. (2012). The Tat system for membrane translocation of folded proteins recruits the membrane-stabilizing Psp machinery in *Escherichia coli*. *J. Biol. Chem.* 287 27834–27842. 10.1074/jbc.M112.374983 22689583 PMC3431631

[B16] Miller J. H. (1972). *Experiments in Molecular Genetics.* Cold Spring Harbor, NY: Cold Spring Harbor Laboratory.

[B17] Monot M. Eckert C. Lemire A. Hamiot A. Dubois T. Tessier C. (2015). *Clostridium difficile*: new insights into the evolution of the pathogenicity locus. *Sci. Rep.* 5:15023. 10.1038/srep15023 26446480 PMC4597214

[B18] Mukherjee K. Karlsson S. Burman L. G. Akerlund T. (2002). Proteins released during high toxin production in *Clostridium difficile*. *Microbiology* 148 2245–2253. 10.1099/00221287-148-7-2245 12101311

[B19] Olling A. Seehase S. Minton N. P. Tatge H. Schröter S. Kohlscheen S. (2012). Release of TcdA and TcdB from *Clostridium difficile* cdi 630 is not affected by functional inactivation of the tcdE gene. *Microb. Pathog.* 52 92–100. 10.1016/j.micpath.2011.10.009 22107906

[B20] Pettersen E. F. Goddard T. D. Huang C. C. Couch G. S. Greenblatt D. M. Meng E. C. (2004). UCSF Chimera–a visualization system for exploratory research and analysis. *J. Comput. Chem.* 25 1605–1612. 10.1002/jcc.20084 15264254

[B21] Reuter J. S. Mathews D. H. (2010). RNAstructure: software for RNA secondary structure prediction and analysis. *BMC Bioinformatics* 11:129. 10.1186/1471-2105-11-129 20230624 PMC2984261

[B22] Riedel T. Bunk B. Thürmer A. Spröer C. Brzuszkiewicz E. Abt B. (2015a). Genome resequencing of the virulent and multidrug-resistant reference strain *Clostridium difficile* 630. *Genome Announc.* 3:e00276-15. 10.1128/genomeA.00276-15 25858846 PMC4392158

[B23] Riedel T. Bunk B. Wittmann J. Thürmer A. Spröer C. Gronow S. (2015b). Complete genome sequence of the *Clostridium difficile* type strain DSM 1296T. *Genome Announc.* 3:e01186-15. 10.1128/genomeA.01186-15 26450746 PMC4599105

[B24] Riedel T. Wetzel D. Hofmann J. D. Plorin S. P. E. O. Dannheim H. Berges M. (2017). High metabolic versatility of different toxigenic and non-toxigenic *Clostridioides difficile* isolates. *Int. J. Med. Microbiol.* 307 311–320. 10.1016/j.ijmm.2017.05.007 28619474

[B25] Saier M. H. Reddy B. L. (2015). Holins in bacteria, eukaryotes, and archaea: multifunctional xenologues with potential biotechnological and biomedical applications. *J. Bacteriol.* 197 7–17. 10.1128/JB.02046-14 25157079 PMC4288690

[B26] Schäffler H. Breitrück A. (2018). *Clostridium difficile* - from colonization to infection. *Front. Microbiol.* 9:646 10.3389/fmicb.2018.00646 PMC5902504 29692762

[B27] Schägger H. (2006). Tricine-SDS-PAGE. *Nat. Protoc.* 1 16–22. 10.1038/nprot.2006.4 17406207

[B28] Shen A. (2012). *Clostridium difficile* toxins: mediators of inflammation. *J. Innate Immun.* 4 149–158. 10.1159/000332946 22237401 PMC3388264

[B29] Soutourina O. A. Monot M. Boudry P. Saujet L. Pichon C. Sismeiro O. (2013). Genome-wide identification of regulatory RNAs in the human pathogen *Clostridium difficile*. *PLoS Genet.* 9:e1003493. 10.1371/journal.pgen.1003493 23675309 PMC3649979

[B30] Tan K. S. Wee B. Y. Song K. P. (2001). Evidence for holin function of tcdE gene in the pathogenicity of *Clostridium difficile*. *J. Med. Microbiol.* 50 613–619. 10.1099/0022-1317-50-7-613 11444771

[B31] Towbin H. Staehelin T. Gordon J. (1979). Electrophoretic transfer of proteins from polyacrylamide gels to nitrocellulose sheets: procedure and some applications. *Proc. Natl. Acad. Sci. U.S.A.* 76 4350–4354. 10.1073/pnas.76.9.4350 388439 PMC411572

[B32] Waterhouse A. Bertoni M. Bienert S. Studer G. Tauriello G. Gumienny R. (2018). SWISS-MODEL: homology modelling of protein structures and complexes. *Nucleic Acids Res.* 46 W296–W303. 10.1093/nar/gky427 29788355 PMC6030848

[B33] Wilms B. Hauck A. Reuss M. Syldatk C. Mattes R. Siemann M. (2001). High-cell-density fermentation for production of L-N-carbamoylase using an expression system based on the *Escherichia coli* rhaBAD promoter. *Biotechnol. Bioeng.* 73 95–103. 10.1002/bit.1041 11255157

[B34] Wohlan K. Goy S. Olling A. Srivaratharajan S. Tatge H. Genth H. (2014). Pyknotic cell death induced by *Clostridium difficile* TcdB: chromatin condensation and nuclear blister are induced independently of the glucosyltransferase activity. *Cell. Microbiol.* 16 1678–1692. 10.1111/cmi.12317 24898616

[B35] Wydau-Dematteis S. El Meouche I. Courtin P. Hamiot A. Lai-Kuen R. Saubaméa B. (2018). Cwp19 is a novel lytic transglycosylase involved in stationary-phase autolysis resulting in toxin release in *Clostridium difficile*. *mBio* 9 e00648-18. 10.1128/mBio.00648-18 29895635 PMC6016235

