# Supplementary material for: Evidence for an Adaptation of a Phage-Derived Holin/Endolysin System to Toxin Transport in Clostridioides difficile
Source: Front Microbiol. 2018 Oct 18;9:2446. doi: 10.3389/fmicb.2018.02446 (PMC6200909; doi:10.3389/fmicb.2018.02446)
Supplement: Supplementary file 1 [file Presentation_1.pdf]

Supplementary Material

Evidence for an adaptation of a phage-derived holin/endolysin system to toxin transport in *Clostridioides difficile*

Denise Mehner-Breitfeld[1], Claudia Rathmann[1], Thomas Riedel[2,3], Ingo Just[4], Ralf Gerhard[4], Jörg Overmann[2,3], and Thomas Brüser[1*]

[1]Institute of Microbiology, Leibniz Universität Hannover, Hannover, Germany

[2]Department of Microbial Ecology and Diversity Research, Leibniz Institute DSMZ-German Collection of Microorganisms and Cell Cultures, Braunschweig, Germany

[3]German Center for Infection Research (DZIF), Partner Site Hannover-Braunschweig, Braunschweig, Germany

[4]Institute of Toxicology, Hannover Medical School, Hannover, Germany

*** Correspondence:** Thomas Brüser; brueser@ifmb.uni-hannover.de

1 Supplementary Figures

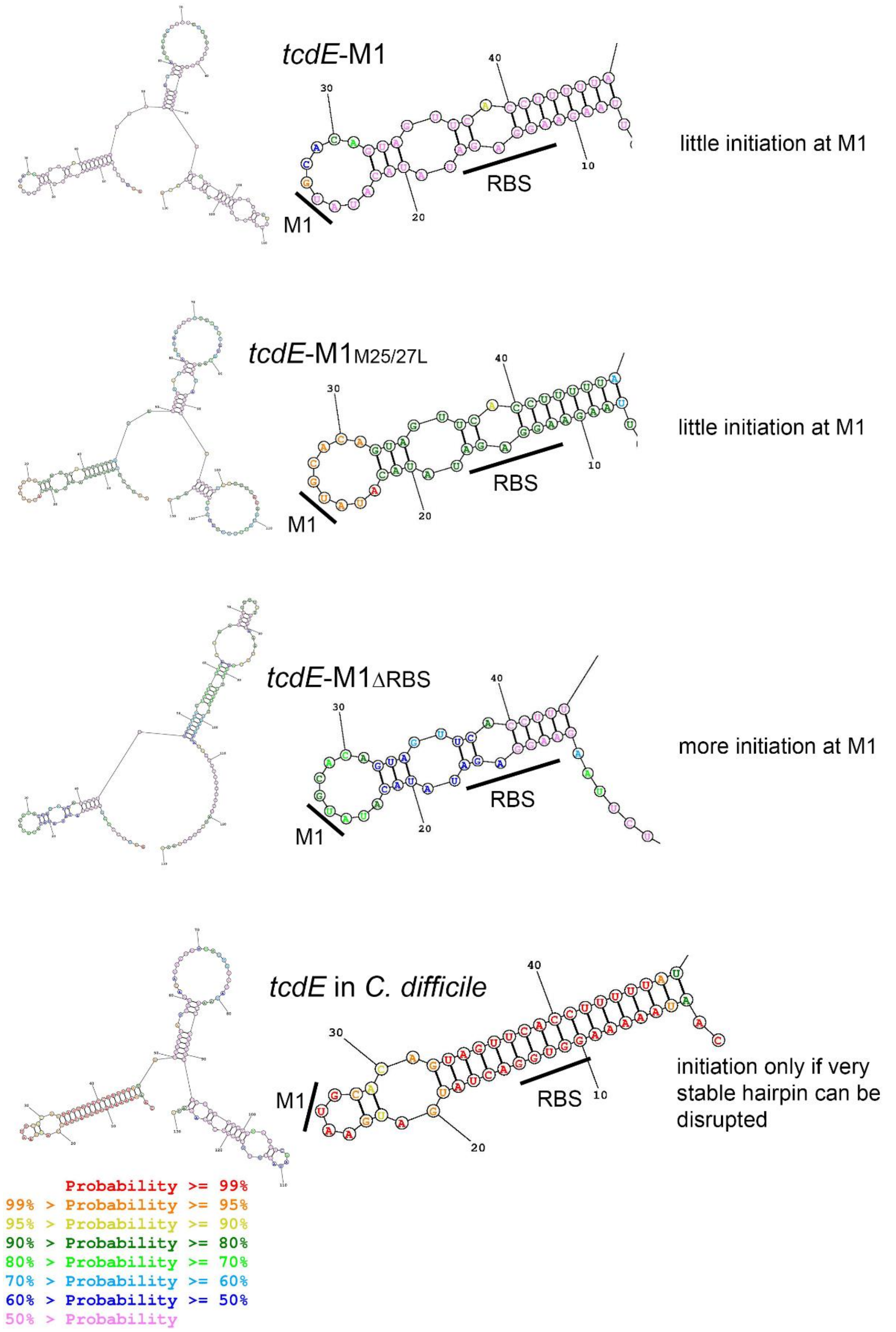

Supplementary Figure S1. Secondary structures around the M1 translational start codon in the analyzed constructs and in genomic *tcdE*. Analyses were carried out using the software RNAstructure (Reuter und Mathews 2010). Probabilities are color-coded as indicated at the bottom.

```
phage-endolysin   ATATCAATAGGTATAAATCAGACTTACAAACAGTTGAGGGAGGAGAATGAATAATGGTAG      264
PaLoc             TTTACTAGATGC-AATGACAGATGAAATGAATGCTAAGGATGAAAAGTAAGTAATGGTAG      811
                  :*::*:* * *  :*:.:****  :*.:.*.:* *.***.:*.*.*.*.*.*********

phage-endolysin   ATATAATAAAGATGTTAACAAAGAAAAAGTGTTATCCAAACAAGAATAGTCCTAAATTCA      324
PaLoc             ATATAATAAAGATATTAACAAATAAAAAGTGTTATCCAAATAAGAATAGCT-GAAAGTTA      870
                  *************.******** ***************** ********    *** * *

phage-endolysin   TAGTGATTCATGAGACTGATAATGAGGACAAGGGAGCAGATGCTAAGAGACATGCACAAG      384
PaLoc             TCATAATTCATGAAACTAATAATGAAAACGAGGGAGCAGATGCCAAGAGACACA--CAAG      928
                  *..*.********.***.*******..**.************* ******** .  ****

phage-endolysin   CACTTAATAATGGTAATTTAGAAGCAAGTGTTCATTACTATGTTGATGATAAAGTAATAT      444
PaLoc             TATTAAATACATATAATTTCGAAGCAAGTGTTCATTACTATATAGATGACAAGGTAGTAT      988
                   * *:****.: .******.*********************.*:***** **.***.***

phage-endolysin   ATCAAACATTAGACCATAAAGATGGAGCGTGGGCAGTTGGTAAAAGCTATGGAACTGCAT      504
PaLoc             ATCAAACATTGGTTCACAAAGATGGTGCATGGTCAGTTGGTAAAATCTATTAAGCTACAT      1048
                  **********.*: ** ********:**.*** ************ **** .*.**.***

phage-endolysin   TAGTTGCAGGAGTTACAAATTACAATAGTATTAATATAGAAATATGTGTAAATCGTGATG      564
PaLoc             TAGTTACAGATATCACAAACTATAATAGTT-AAACATAGAAATATGTGTAAATTGTGATG      1107
                  *****.***.:.* ***** ** ******: :** ****************** ******

phage-endolysin   GTAATTACACAAAAGCAAGA-----------------CAAAATGCAATAGATTTAACTAG      607
PaLoc             GAAATTATTCAAAAACACAAAAATACGTGATGAAGGACAAAATGATATAGAAAATAAGTA      1167
                  *:***** :*****.**..*                 *******.:*****::::*. :.

phage-endolysin   -AAA-ACTCA-TGAAAGATTTAAATATTAGTAC---AGATAAAG---------------T      646
PaLoc             TCAAACCTTAATAAATGATTTAATTGATAGTTTAAAAGTTATAGGAAAAATATATAAAGA      1227
                   .** .** * *.**:*******:*.:****:    **:**:**               :

phage-endolysin   AAT-AAGACACTATGATGCTAAGAGAAAATATTGTCCTCGTA--AGATGCTT-GATAATC      702
PaLoc             AATAAAAACATTAAAAAAATATAAGAT----ATGTTTACAAATTACTATCAGACAATCTC      1283
                  *** **.*** **:.*:..**:.***:    :***  :*.:*  * :: *:   *::.**

phage-endolysin   CAAAATTATGGGTAGA-----TTTTAAAGATAAGA---TAA-----AAAATGATGGGGGT      749
PaLoc             CTTATCTAATAGAAGAGTCAATTAACTAATTGAGTATCTTTAAATTGAAATGTT-AGGAA      1342
                  *::*: **: .*:***     **::.:*.:*.**:   *::     .*****:* .**.:

phage-endolysin   GTGAAT--AGTATGATAAAATA----CACAATAGTCTATGATGGAGAGGTTGACAGAATA      803
PaLoc             GTGATTTAAATATGAAAACTTAAATTATAAAAAATCAAT---------------------      1381
                  ****:*  *.*****:**.:**    .:.**:*.**:**

phage-endolysin   CCAGCCGAGGTTATTGGTTGGAATTATAAGAAAGAAGAATGTAGAGTATGTGATAT----      859
PaLoc             ---------ATTAA---TT--TATTTTTAAAAAATAGAAAGGAGTGTATAAGATTTATTT      1427
                           .***:   **  :***:*:*.***.:****:* **:****.:***:*

phage-endolysin   -AAAAGAATATATACCTGGTCAGACGC----AAAATCTGTATAT-TGTAGGTGGGGGAGC      913
PaLoc             TCAAAGTTTAAAAACA-AGAAAATCAATTTAAATTTCAGAAGGAATAAATGTGG------      1480
                   .****::**:*:**. .*:.*.:*..    **::**:*:* .: *.:* ****

phage-endolysin   ATGTAATAAGATAGGTTC-TATAACAAAAGAACATTATACAATGATAAAGGGTAATGATA      972
PaLoc             ---TTATAGAAGTGGATTTATTATCAAAA-ATAATAATACTAGGA-----GGTTT-----      1526
                     *:***..* :**:*  ::**:***** *:.**:****:* **     ***::

phage-endolysin   GGTTTGAAACACTTTACAAAGCATTAGATTTTATTAAGAAATAA      1016
PaLoc             ---------------------------------TTATG------      1531
                                                   ***:*
```

Supplementary Figure S2. TcdL, an endolysin fragment encoded in PaLoc. Clustal Omega based comparison of the PaLoc sequence with the endolysin-encoding sequence of phage phiCD481-1. The *tcdL*-encoding region is highlighted in blue, the phage endolysin-encoding sequence is highlighted in grey. Translational starts and stops are highlighted in yellow, ribosomal binding sites in green. Note that the Shine-Dalgarno sequence of the phage endolysine is mutated in PaLoc, a frame shift (pink) generated the translational start for *tcdL*, and the Shine-Dalgarno sequence for *tcdL* is optimized by a A>G transition.

2 Supplementary Data

(A) Sequence alignment for all 44 analyzed sequences of the PaLoc region, ranging from the translational stop of *tcdB* to the translational start of *tcdA*. The alignment was generated using Clustal Omega (Sievers and Higgins, 2014). Mutations that are found in minorities of sequences are highlighted in blue. The positions of translational starts and stops are highlighted in yellow and indicated at the bottom of the sequences. (B) All analyzed sequences in FASTA format. Strain names are given as DSM numbers, except for strains of previously deposited sequences.

(A)

```
M68                     ATAAAAATATGTTAAATATATCCTCTTATACTTAAATATATAAAAATAAACAAAATGA      58
CF5                     ATAAAAATATGTTAAATATATCCTCTTATACTTAAATATATAAAAATAAACAAAATGA      58
DSM 29095               ATAAAAATATGTTAAATATATCCTCTTATACTTAAATATATAAAAATAAACAAAATGA      58
DSM 29090               ATAAAAATATGTTAAATATATCCTCTTATACTTAAATATATAAAAATAAACAAAATGA      58
DSM 28194               ATAAAAATATGTTAAATATATCCTCTTATACTTAAATATATAAAAATAAACAAAATGA      58
DSM 28197               ATAAAAATATGTTAAATATATCCTCTTATACTTAAATATATAAAAATAAACAAAATGA      58
DSM 1296T               ATAAAAATATGTTAAATATATCCTCTTATACTTAAATATATAAAAATAAACAAAATGA      58
DSM 27639               ATAAAAATATGTTAAATATATCCTCTTATACTTAAATATATAAAAATAAACAAAATGA      58
DSM 29675               ATAAAAATATGTTAAATATATCCTCTTATACTTAAATATATAAAAATAAACAAAATGA      58
DSM 29676               ATAAAAATATGTTAAATATATCCTCTTATACTTAAATATATAAAAATAAACAAAATGA      58
DSM 29677               ATAAAAATATGTTAAATATATCCTCTTATACTTAAATATATAAAAATAAACAAAATGA      58
DSM 29678               ATAAAAATATGTTAAATATATCCTCTTATACTTAAATATATAAAAATAAACAAAATGA      58
DSM 29679               ATAAAAATATGTTAAATATATCCTCTTATACTTAAATATATAAAAATAAACAAAATGA      58
DSM 29683               ATAAAAATATGTTAAATATATCCTCTTATACTTAAATATATAAAAATAAACAAAATGA      58
DSM 29684               ATAAAAATATGTTAAATATATCCTCTTATACTTAAATATATAAAAATAAACAAAATGA      58
DSM 29685               ATAAAAATATGTTAAATATATCCTCTTATACTTAAATATATAAAAATAAACAAAATGA      58
DSM 29686               ATAAAAATATGTTAAATATATCCTCTTATACTTAAATATATAAAAATAAACAAAATGA      58
DSM 29089               ATAAAAATATGTTAAATATATCCTCTTATACTTAAATATATAAAAATAAACAAAATGA      58
DSM 29689               ATAAAAATATGTTAAATATATCCTCTTATACTTAAATATATAAAAATAAACAAAATGA      58
DSM 29692               ATAAAAATATGTTAAATATATCCTCTTATACTTAAATATATAAAAATAAACAAAATGA      58
DSM 29091               ATAAAAATATGTTAAATATATCCTCTTATACTTAAATATATAAAAATAAACAAAATGA      58
630                     ATAAAAATATGTTAAATATATCCTCTTATACTTAAATATATAAAAATAAACAAAATGA      58
630 delta erm           ATAAAAATATGTTAAATATATCCTCTTATACTTAAATATATAAAAATAAACAAAATGA      58
DSM 29744               ATAAAAATATGTTAAATATATCCTCTTATACTTAAATATATAAAAATAAACAAAATGA      58
DSM 29745               ATAAAAATATGTTAAATATATCCTCTTATACTTAAATATATAAAAATAAACAAAATGA      58
DSM 29754               ATAAAAATATGTTAAATATATCCTCTTATACTTAAATATATAAAAATAAACAAAATGA      58
DSM 29687               ATAAAAATATGTTAAATATATCCTCTTATACTTAAATATATAAAAATAAACAAAATGA      58
DSM 29690               ATAAAAATATGTTAAATATATCCTCTTATACTTAAATATATAAAAATAAACAAAATGA      58
DSM 29691               ATAAAAATATGTTAAATATATCCTCTTATACTTAAATATATAAAAATAAACAAAATGA      58
DSM 29092               ATAAAAATATGTTAAATATATCCTCTTATACTTAAATATATAAAAATAAACAAAATGA      58
DSM 29093               ATAAAAATATGTTAAATATATCCTCTTATACTTAAATATATAAAAATAAACAAAATGA      58
DSM 29756               ATAAAAATATGTTAAATATATCCTCTTATACTTAAATATATAAAAATAAACAAAATGA      58
DSM 29094               ATAAAAATATGTTAAATATATCCTCTTATACTTAAATATATAAAAATAAACAAAATGA      58
2007855                 ATAAAAATACGTTAAATATATCCTCTTATACTTAAATATATAAAAATAAACAAAATGA      58
BI1                     ATAAAAATACGTTAAATATATCCTCTTATACTTAAATATATAAAAATAAACAAAATGA      58
DSM 28196               ATAAAAATACGTTAAATATATCCTCTTATACTTAAATATATAAAAATAAACAAAATGA      58
DSM 27638               ATAAAAATACGTTAAATATATCCTCTTATACTTAAATATATAAAAATAAACAAAATGA      58
DSM 27640               ATAAAAATACGTTAAATATATCCTCTTATACTTAAATATATAAAAATAAACAAAATGA      58
DSM 29680               ATAAAAATACGTTAAATATATCCTCTTATACTTAAATATATAAAAATAAACAAAATGA      58
R20291                  ATAAAAATACGTTAAATATATCCTCTTATACTTAAATATATAAAAATAAACAAAATGA      58
DSM 29747               ATAAAAATATGTTAAATATATCCTCTTATACTTAAATATATAAAAATAAACAAAATGA      58
DSM 29752               ATAAAAATATGTTAAATATATCCTCTTATACTTAAATATATAAAAATAAACAAAATGA      58
DSM 29753               ATAAAAATATGTTAAATATATCCTCTTATACTTAAATATATAAAAATAAACAAAATGA      58
M120                    ATAAAAATATGTTAAATATATCCTCTTATACTTAAATATATAAAAATAAACAAAATGA      58
                        ********* ************************************************
```

```
M68              TACACTACATAAAGTGTTCTATCTAATATGAAGATTTACCAATAAAAAGGTGGACTATGA     118
CF5              TACACTACATAAAGTGTTCTATCTAATATGAAGATTTACCAATAAAAAGGTGGACTATGA     118
DSM-29095        TACACTACATAAAGTGTTCTATCTAATATGAAGATTTACCAATAAAAAGGTGGACTATGA     118
DSM-29090        TACACTACATAAAGTGTTCTATCTAATATGAAGATTTACCAATAAAAAGGTGGACTATGA     118
DSM-28194        TACACTACATAAAGTGTTCTATCTAATATGAAGATTTACCAATAAAAAGGTGGACTATGA     118
DSM-28197        TACACTACATAAAGTGTTCTATCTAATATGAAGATTTACCAATAAAAAGGTGGACTATGA     118
DSM-1296T        TACACTACATAAAGTGTTCTATCTAATATGAAGATTTACCAATAAAAAGGTGGACTATGA     118
DSM-27639        TACACTACATAAAGTGTTCTATCTAATATGAAGATTTACCAATAAAAAGGTGGACTATGA     118
DSM-29675        TACACTACATAAAGTGTTCTATCTAATATGAAGATTTACCAATAAAAAGGTGGACTATGA     118
DSM-29676        TACACTACATAAAGTGTTCTATCTAATATGAAGATTTACCAATAAAAAGGTGGACTATGA     118
DSM-29677        TACACTACATAAAGTGTTCTATCTAATATGAAGATTTACCAATAAAAAGGTGGACTATGA     118
DSM-29678        TACACTACATAAAGTGTTCTATCTAATATGAAGATTTACCAATAAAAAGGTGGACTATGA     118
DSM-29679        TACACTACATAAAGTGTTCTATCTAATATGAAGATTTACCAATAAAAAGGTGGACTATGA     118
DSM-29683        TACACTACATAAAGTGTTCTATCTAATATGAAGATTTACCAATAAAAAGGTGGACTATGA     118
DSM-29684        TACACTACATAAAGTGTTCTATCTAATATGAAGATTTACCAATAAAAAGGTGGACTATGA     118
DSM-29685        TACACTACATAAAGTGTTCTATCTAATATGAAGATTTACCAATAAAAAGGTGGACTATGA     118
DSM-29686        TACACTACATAAAGTGTTCTATCTAATATGAAGATTTACCAATAAAAAGGTGGACTATGA     118
DSM-29089        TACACTACATAAAGTGTTCTATCTAATATGAAGATTTACCAATAAAAAGGTGGACTATGA     118
DSM-29689        TACACTACATAAAGTGTTCTATCTAATATGAAGATTTACCAATAAAAAGGTGGACTATGA     118
DSM-29692        TACACTACATAAAGTGTTCTATCTAATATGAAGATTTACCAATAAAAAGGTGGACTATGA     118
DSM-29091        TACACTACATAAAGTGTTCTATCTAATATGAAGATTTACCAATAAAAAGGTGGACTATGA     118
630              TACACTACATAAAGTGTTCTATCTAATATGAAGATTTACCAATAAAAAGGTGGACTATGA     118
630-delta-erm    TACACTACATAAAGTGTTCTATCTAATATGAAGATTTACCAATAAAAAGGTGGACTATGA     118
DSM-29744        TACACTACATAAAGTGTTCTATCTAATATGAAGATTTACCAATAAAAAGGTGGACTATGA     118
DSM-29745        TACACTACATAAAGTGTTCTATCTAATATGAAGATTTACCAATAAAAAGGTGGACTATGA     118
DSM-29754        TACACTACATAAAGTGTTCTATCTAATATGAAGATTTACCAATAAAAAGGTGGACTATGA     118
DSM-29687        TACACTACATAAAGTGTTCTATCTAATATGAAGATTTACCAATAAAAAGGTGGACTATGA     118
DSM-29690        TACACTACATAAAGTGTTCTATCTAATATGAAGATTTACCAATAAAAAGGTGGACTATGA     118
DSM-29691        TACACTACATAAAGTGTTCTATCTAATATGAAGATTTACCAATAAAAAGGTGGACTATGA     118
DSM-29092        TACACTACATAAAGTGTTCTATCTAATATGAAGATTTACCAATAAAAAGGTGGACTATGA     118
DSM-29093        TACACTACATAAAGTGTTCTATCTAATATGAAGATTTACCAATAAAAAGGTGGACTATGA     118
DSM-29756        TACACTACATAAAGTGTTCTATCTAATATGAAGATTTACCAATAAAAAGGTGGACTATGA     118
DSM-29094        TACACTACATAAAGTGTTCTATCTAATATGAAGATTTACCAATAAAAAGGTGGACTATGA     118
2007855          TACACTACATAAAGTGTTCTATCTAATATGAAGATTTACCAATAAAAAGGTGAACTATGA     118
BI1              TACACTACATAAAGTGTTCTATCTAATATGAAGATTTACCAATAAAAAGGTGAACTATGA     118
DSM-28196        TACACTACATAAAGTGTTCTATCTAATATGAAGATTTACCAATAAAAAGGTGAACTATGA     118
DSM-27638        TACACTACATAAAGTGTTCTATCTAATATGAAGATTTACCAATAAAAAGGTGAACTATGA     118
DSM-27640        TACACTACATAAAGTGTTCTATCTAATATGAAGATTTACCAATAAAAAGGTGAACTATGA     118
DSM-29680        TACACTACATAAAGTGTTCTATCTAATATGAAGATTTACCAATAAAAAGGTGAACTATGA     118
R20291           TACACTACATAAAGTGTTCTATCTAATATGAAGATTTACCAATAAAAAGGTGAACTATGA     118
DSM-29747        TACACTACATAAAGTGTTCTATCTAATATGAAGATTTACCAATAAAAAGGTGAACTATGA     118
DSM-29752        TACACTACATAAAGTGTTCTATCTAATATGAAGATTTACCAATAAAAAGGTGAACTATGA     118
DSM-29753        TACACTACATAAAGTGTTCTATCTAATATGAAGATTTACCAATAAAAAGGTGAACTATGA     118
M120             TACACTACATAAAGTGTTCTATCTAATATGAAGATTTACCAATAAAAAGGTGAACTATGA     118
                 **************************************************** *******
```

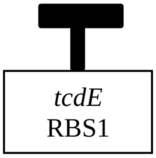

```
M68                 TGAATGCACAGTAGTTCACCTTTTTATATTTCTAATGGTAACAAAATATTTTTTTATATA       178
CF5                 TGAATGCACAGTAGTTCACCTTTTTATATTTCTAATGGTAACAAAATATTTTTTTATATA       178
DSM-29095           TGAATGCACAGTAGTTCACCTTTTTATATTTCTAATGGTAACAAAATATTTTTTTATATA       178
DSM-29090           TGAATGCACAGTAGTTCACCTTTTTATATTTCTAATGGTAACAAAATATTTTTTTATATA       178
DSM-28194           TGAATGCACAGTAGTTCACCTTTTTATATTTCTAATGGTAACAAAATATTTTTTTATATA       178
DSM-28197           TGAATGCACAGTAGTTCACCTTTTTATATTTCTAATGGTAACAAAATATTTTTTTATATA       178
DSM-1296T           TGAATGCACAGTAGTTCACCTTTTTATATTTCTAATGGTAACAAAATATTTTTTTATATA       178
DSM-27639           TGAATGCACAGTAGTTCACCTTTTTATATTTCTAATGGTAACAAAATATTTTTTTATATA       178
DSM-29675           TGAATGCACAGTAGTTCACCTTTTTATATTTCTAATGGTAACAAAATATTTTTTTATATA       178
DSM-29676           TGAATGCACAGTAGTTCACCTTTTTATATTTCTAATGGTAACAAAATATTTTTTTATATA       178
DSM-29677           TGAATGCACAGTAGTTCACCTTTTTATATTTCTAATGGTAACAAAATATTTTTTTATATA       178
DSM-29678           TGAATGCACAGTAGTTCACCTTTTTATATTTCTAATGGTAACAAAATATTTTTTTATATA       178
DSM-29679           TGAATGCACAGTAGTTCACCTTTTTATATTTCTAATGGTAACAAAATATTTTTTTATATA       178
DSM-29683           TGAATGCACAGTAGTTCACCTTTTTATATTTCTAATGGTAACAAAATATTTTTTTATATA       178
DSM-29684           TGAATGCACAGTAGTTCACCTTTTTATATTTCTAATGGTAACAAAATATTTTTTTATATA       178
DSM-29685           TGAATGCACAGTAGTTCACCTTTTTATATTTCTAATGGTAACAAAATATTTTTTTATATA       178
DSM-29686           TGAATGCACAGTAGTTCACCTTTTTATATTTCTAATGGTAACAAAATATTTTTTTATATA       178
DSM-29089           TGAATGCACAGTAGTTCACCTTTTTATATTTCTAATGGTAACAAAATATTTTTTTATATA       178
DSM-29689           TGAATGCACAGTAGTTCACCTTTTTATATTTCTAATGGTAACAAAATATTTTTTTATATA       178
DSM-29692           TGAATGCACAGTAGTTCACCTTTTTATATTTCTAATGGTAACAAAATATTTTTTTATATA       178
DSM-29091           TGAATGCACAGTAGTTCACCTTTTTATATTTCTAATGGTAACAAAATATTTTTTTATATA       178
630                 TGAATGCACAGTAGTTCACCTTTTTATATTTCTAATGGTAACAAAATATTTTTTTATATA       178
630-delta-erm       TGAATGCACAGTAGTTCACCTTTTTATATTTCTAATGGTAACAAAATATTTTTTTATATA       178
DSM-29744           TGAATGCACAGTAGTTCACCTTTTTATATTTCTAATGGTAACAAAATATTTTTTTATATA       178
DSM-29745           TGAATGCACAGTAGTTCACCTTTTTATATTTCTAATGGTAACAAAATATTTTTTTATATA       178
DSM-29754           TGAATGCACAGTAGTTCACCTTTTTATATTTCTAATGGTAACAAAATATTTTTTTATATA       178
DSM-29687           TGAATGCACAGTAGTTCACCTTTTTATATTTCTAATGGTAACAAAATATTTTTTTATATA       178
DSM-29690           TGAATGCACAGTAGTTCACCTTTTTATATTTCTAATGGTAACAAAATATTTTTTTATATA       178
DSM-29691           TGAATGCACAGTAGTTCACCTTTTTATATTTCTAATGGTAACAAAATATTTTTTTATATA       178
DSM-29092           TGAATGCACAGTAGTTCACCTTTTTATATTTCTAATGGTAACAAAATATTTTTTTATATA       178
DSM-29093           TGAATGCACAGTAGTTCACCTTTTTATATTTCTAATGGTAACAAAATATTTTTTTATATA       178
DSM-29756           TGAATGCACAGTAGTTCACCTTTTTATATTTCTAATGGTAACAAAATATTTTTTTATATA       178
DSM-29094           TGAATGCACAGTAGTTCACCTTTTTATATTTCTAATGGTAACAAAATATTTTTTTATATA       178
2007855             TGAATGCACAGTAGTTCACCTTTTTATATTTCTAATGGTAACAAAATATTTTTTTATATA       178
BI1                 TGAATGCACAGTAGTTCACCTTTTTATATTTCTAATGGTAACAAAATATTTTTTTATATA       178
DSM-28196           TGAATGCACAGTAGTTCACCTTTTTATATTTCTAATGGTAACAAAATATTTTTTTATATA       178
DSM-27638           TGAATGCACAGTAGTTCACCTTTTTATATTTCTAATGGTAACAAAATATTTTTTTATATA       178
DSM-27640           TGAATGCACAGTAGTTCACCTTTTTATATTTCTAATGGTAACAAAATATTTTTTTATATA       178
DSM-29680           TGAATGCACAGTAGTTCACCTTTTTATATTTCTAATGGTAACAAAATATTTTTTTATATA       178
R20291              TGAATGCACAGTAGTTCACCTTTTTATATTTCTAATGGTAACAAAATATTTTTTTATATA       178
DSM-29747           TGAATTCACAGTAGTTCACCTTTTTATATTTCTAATGGTAACAAAATATTTTTTTATATA       178
DSM-29752           TGAATTCACAGTAGTTCACCTTTTTATATTTCTAATGGTAACAAAATATTTTTTTATATA       178
DSM-29753           TGAATTCACAGTAGTTCACCTTTTTATATTTCTAATGGTAACAAAATATTTTTTTATATA       178
M120                TGAATTCACAGTAGTTCACCTTTTTATATTTCTAATGGTAACAAAATATTTTTTTATATA       178
                    ***** ******************************************************
```

tcdE translational start M1

```
M68              AACCTAGGAGGCGTTATGAATATGACAATATCTTTTTTATCAGAGCATATATTTATAAAG   238
CF5              AACCTAGGAGGCGTTATGAATATGACAATATCTTTTTTATCAGAGCATATATTTATAAAG   238
DSM-29095        AACCTAGGAGGCGTTATGAATATGACAATATCTTTTTTATCAGAGCATATATTTATAAAG   238
DSM-29090        AACCTAGGAGGCGTTATGAATATGACAATATCTTTTTTATCAGAGCATATATTTATAAAG   238
DSM-28194        AACCTAGGAGGCGTTATGAATATGACAATATCTTTTTTATCAGAGCATATATTTATAAAG   238
DSM-28197        AACCTAGGAGGCGTTATGAATATGACAATATCTTTTTTATCAGAGCATATATTTATAAAG   238
DSM-1296T        AACCTAGGAGGCGTTATGAATATGACAATATCTTTTTTATCAGAGCATATATTTATAAAG   238
DSM-27639        AACCTAGGAGGCGTTATGAATATGACAATATCTTTTTTATCAGAGCATATATTTATAAAG   238
DSM-29675        AACCTAGGAGGCGTTATGAATATGACAATATCTTTTTTATCAGAGCATATATTTATAAAG   238
DSM-29676        AACCTAGGAGGCGTTATGAATATGACAATATCTTTTTTATCAGAGCATATATTTATAAAG   238
DSM-29677        AACCTAGGAGGCGTTATGAATATGACAATATCTTTTTTATCAGAGCATATATTTATAAAG   238
DSM-29678        AACCTAGGAGGCGTTATGAATATGACAATATCTTTTTTATCAGAGCATATATTTATAAAG   238
DSM-29679        AACCTAGGAGGCGTTATGAATATGACAATATCTTTTTTATCAGAGCATATATTTATAAAG   238
DSM-29683        AACCTAGGAGGCGTTATGAATATGACAATATCTTTTTTATCAGAGCATATATTTATAAAG   238
DSM-29684        AACCTAGGAGGCGTTATGAATATGACAATATCTTTTTTATCAGAGCATATATTTATAAAG   238
DSM-29685        AACCTAGGAGGCGTTATGAATATGACAATATCTTTTTTATCAGAGCATATATTTATAAAG   238
DSM-29686        AACCTAGGAGGCGTTATGAATATGACAATATCTTTTTTATCAGAGCATATATTTATAAAG   238
DSM-29089        AACCTAGGAGGCGTTATGAATATGACAATATCTTTTTTATCAGAGCATATATTTATAAAG   238
DSM-29689        AACCTAGGAGGCGTTATGAATATGACAATATCTTTTTTATCAGAGCATATATTTATAAAG   238
DSM-29692        AACCTAGGAGGCGTTATGAATATGACAATATCTTTTTTATCAGAGCATATATTTATAAAG   238
DSM-29091        AACCTAGGAGGCGTTATGAATATGACAATATCTTTTTTATCAGAGCATATATTTATAAAG   238
630              AACCTAGGAGGCGTTATGAATATGACAATATCTTTTTTATCAGAGCATATATTTATAAAG   238
630-delta-erm    AACCTAGGAGGCGTTATGAATATGACAATATCTTTTTTATCAGAGCATATATTTATAAAG   238
DSM-29744        AACCTAGGAGGCGTTATGAATATGACAATATCTTTTTTATCAGAGCATATATTTATAAAG   238
DSM-29745        AACCTAGGAGGCGTTATGAATATGACAATATCTTTTTTATCAGAGCATATATTTATAAAG   238
DSM-29754        AACCTAGGAGGCGTTATGAATATGACAATATCTTTTTTATCAGAGCATATATTTATAAAG   238
DSM-29687        AACCTAGGAGGCGTTATGAATATGACAATATCTTTTTTATCAGAGCATATATTTATAAAG   238
DSM-29690        AACCTAGGAGGCGTTATGAATATGACAATATCTTTTTTATCAGAGCATATATTTATAAAG   238
DSM-29691        AACCTAGGAGGCGTTATGAATATGACAATATCTTTTTTATCAGAGCATATATTTATAAAG   238
DSM-29092        AACCTAGGAGGCGTTATGAATATGACAATATCTTTTTTATCAGAGCATATATTTATAAAG   238
DSM-29093        AACCTAGGAGGCGTTATGAATATGACAATATCTTTTTTATCAGAGCATATATTTATAAAG   238
DSM-29756        AACCTAGGAGGCGTTATGAATATGACAATATCTTTTTTATCAGAGCATATATTTATAAAG   238
DSM-29094        AACCTAGGAGGCGTTATGAATATGACAATATCTTTTTTATCAGAGCATATATTTATAAAG   238
2007855          AACCTAGGAGGCGTTATGAATATGACAATATCTTTTTTATCAGAGCATATATTTATAAAG   238
BI1              AACCTAGGAGGCGTTATGAATATGACAATATCTTTTTTATCAGAGCATATATTTATAAAG   238
DSM-28196        AACCTAGGAGGCGTTATGAATATGACAATATCTTTTTTATCAGAGCATATATTTATAAAG   238
DSM-27638        AACCTAGGAGGCGTTATGAATATGACAATATCTTTTTTATCAGAGCATATATTTATAAAG   238
DSM-27640        AACCTAGGAGGCGTTATGAATATGACAATATCTTTTTTATCAGAGCATATATTTATAAAG   238
DSM-29680        AACCTAGGAGGCGTTATGAATATGACAATATCTTTTTTATCAGAGCATATATTTATAAAG   238
R20291           AACCTAGGAGGCGTTATGAATATGACAATATCTTTTTTATCAGAGCATATATTTATAAAG   238
DSM-29747        AACCTAGGAGGCGTTATGAATATGACAATATCTTTTTTATCAGAGCATATATTTATAAAG   238
DSM-29752        AACCTAGGAGGCGTTATGAATATGACAATATCTTTTTTATCAGAGCATATATTTATAAAG   238
DSM-29753        AACCTAGGAGGCGTTATGAATATGACAATATCTTTTTTATCAGAGCATATATTTATAAAG   238
M120             AACCTAGGAGGCGTTATGAATATGACAATATCTTTTTTATCAGAGCATATATTTATAAAG   238
                 ************************************************************
```

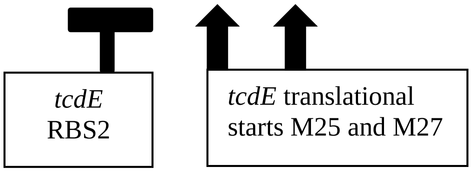

```
M68                 TTAGTAATTTTAACTATATCATTTGATACATTATTAGGATGTTTAAGTGCAATAAAAAGT       298
CF5                 TTAGTAATTTTAACTATATCATTTGATACATTATTAGGATGTTTAAGTGCAATAAAAAGT       298
DSM-29095           TTAGTAATTTTAACTATATCATTTGATACATTATTAGGATGTTTAAGTGCAATAAAAAGT       298
DSM-29090           TTAGTAATTTTAACTATATCATTTGATACATTATTAGGATGTTTAAGTGCAATAAAAAGT       298
DSM-28194           TTAGTAATTTTAACTATATCATTTGATACATTATTAGGATGTTTAAGTGCAATAAAAAGT       298
DSM-28197           TTAGTAATTTTAACTATATCATTTGATACATTATTAGGATGTTTAAGTGCAATAAAAAGT       298
DSM-1296T           TTAGTAATTTTAACTATATCATTTGATACATTATTAGGATGTTTAAGTGCAATAAAAAGT       298
DSM-27639           TTAGTAATTTTAACTATATCATTTGATACATTATTAGGATGTTTAAGTGCAATAAAAAGT       298
DSM-29675           TTAGTAATTTTAACTATATCATTTGATACATTATTAGGATGTTTAAGTGCAATAAAAAGT       298
DSM-29676           TTAGTAATTTTAACTATATCATTTGATACATTATTAGGATGTTTAAGTGCAATAAAAAGT       298
DSM-29677           TTAGTAATTTTAACTATATCATTTGATACATTATTAGGATGTTTAAGTGCAATAAAAAGT       298
DSM-29678           TTAGTAATTTTAACTATATCATTTGATACATTATTAGGATGTTTAAGTGCAATAAAAAGT       298
DSM-29679           TTAGTAATTTTAACTATATCATTTGATACATTATTAGGATGTTTAAGTGCAATAAAAAGT       298
DSM-29683           TTAGTAATTTTAACTATATCATTTGATACATTATTAGGATGTTTAAGTGCAATAAAAAGT       298
DSM-29684           TTAGTAATTTTAACTATATCATTTGATACATTATTAGGATGTTTAAGTGCAATAAAAAGT       298
DSM-29685           TTAGTAATTTTAACTATATCATTTGATACATTATTAGGATGTTTAAGTGCAATAAAAAGT       298
DSM-29686           TTAGTAATTTTAACTATATCATTTGATACATTATTAGGATGTTTAAGTGCAATAAAAAGT       298
DSM-29089           TTAGTAATTTTAACTATATCATTTGATACATTATTAGGATGTTTAAGTGCAATAAAAAGT       298
DSM-29689           TTAGTAATTTTAACTATATCATTTGATACATTATTAGGATGTTTAAGTGCAATAAAAAGT       298
DSM-29692           TTAGTAATTTTAACTATATCATTTGATACATTATTAGGATGTTTAAGTGCAATAAAAAGT       298
DSM-29091           TTAGTAATTTTAACTATATCATTTGATACATTATTAGGATGTTTAAGTGCAATAAAAAGT       298
630                 TTAGTAATTTTAACTATATCATTTGATACATTATTAGGATGTTTAAGTGCAATAAAAAGT       298
630-delta-erm       TTAGTAATTTTAACTATATCATTTGATACATTATTAGGATGTTTAAGTGCAATAAAAAGT       298
DSM-29744           TTAGTAATTTTAACTATATCATTTGATACATTATTAGGATGTTTAAGTGCAATAAAAAGT       298
DSM-29745           TTAGTAATTTTAACTATATCATTTGATACATTATTAGGATGTTTAAGTGCAATAAAAAGT       298
DSM-29754           TTAGTAATTTTAACTATATCATTTGATACATTATTAGGATGTTTAAGTGCAATAAAAAGT       298
DSM-29687           TTAGTAATTTTAACTATATCATTTGATACATTATTAGGATGTTTAAGTGCAATAAAAAGT       298
DSM-29690           TTAGTAATTTTAACTATATCATTTGATACATTATTAGGATGTTTAAGTGCAATAAAAAGT       298
DSM-29691           TTAGTAATTTTAACTATATCATTTGATACATTATTAGGATGTTTAAGTGCAATAAAAAGT       298
DSM-29092           TTAGTAATTTTAACTATATCATTTGATACATTATTAGGATGTTTAAGTGCAATAAAAAGT       298
DSM-29093           TTAGTAATTTTAACTATATCATTTGATACATTATTAGGATGTTTAAGTGCAATAAAAAGT       298
DSM-29756           TTAGTAATTTTAACTATATCATTTGATACATTATTAGGATGTTTAAGTGCAATAAAAAGT       298
DSM-29094           TTAGTAATTTTAACTATATCATTTGATACATTATTAGGATGTTTAAGTGCAATAAAAAGT       298
2007855             TTAGTAATTTTAACTATATCATTTGATACATTATTAGGATGTTTAAGTGCAATAAAAAGT       298
BI1                 TTAGTAATTTTAACTATATCATTTGATACATTATTAGGATGTTTAAGTGCAATAAAAAGT       298
DSM-28196           TTAGTAATTTTAACTATATCATTTGATACATTATTAGGATGTTTAAGTGCAATAAAAAGT       298
DSM-27638           TTAGTAATTTTAACTATATCATTTGATACATTATTAGGATGTTTAAGTGCAATAAAAAGT       298
DSM-27640           TTAGTAATTTTAACTATATCATTTGATACATTATTAGGATGTTTAAGTGCAATAAAAAGT       298
DSM-29680           TTAGTAATTTTAACTATATCATTTGATACATTATTAGGATGTTTAAGTGCAATAAAAAGT       298
R20291              TTAGTAATTTTAACTATATCATTTGATACATTATTAGGATGTTTAAGTGCAATAAAAAGT       298
DSM-29747           TTAGTAATTTTAACTATATCATTTGATACATTATTAGGATGTTTAAGTGCAATAAAAAGT       298
DSM-29752           TTAGTAATTTTAACTATATCATTTGATACATTATTAGGATGTTTAAGTGCAATAAAAAGT       298
DSM-29753           TTAGTAATTTTAACTATATCATTTGATACATTATTAGGATGTTTAAGTGCAATAAAAAGT       298
M120                TTAGTAATTTTAACTATATCATTTGATACATTATTAGGATGTTTAAGTGCAATAAAAAGT       298
                    ************************************************************
```

```
M68               CGTAAATTTAATTCTAGTTTTGGAATAGATGGAGGAATCAGAAAAGTAGCAATGATAGCA     358
CF5               CGTAAATTTAATTCTAGTTTTGGAATAGATGGAGGAATCAGAAAAGTAGCAATGATAGCA     358
DSM-29095         CGTAAATTTAATTCTAGTTTTGGAATAGATGGAGGAATCAGAAAAGTAGCAATGATAGCA     358
DSM-29090         CGTAAATTTAATTCTAGTTTTGGAATAGATGGAGGAATCAGAAAAGTAGCAATGATAGCA     358
DSM-28194         CGTAAATTTAATTCTAGTTTTGGAATAGATGGAGGAATCAGAAAAGTAGCAATGATAGCA     358
DSM-28197         CGTAAATTTAATTCTAGTTTTGGAATAGATGGAGGAATCAGAAAAGTAGCAATGATAGCA     358
DSM-1296T         CGTAAATTTAATTCTAGTTTTGGAATAGATGGAGGAATCAGAAAAGTAGCAATGATAGCA     358
DSM-27639         CGTAAATTTAATTCTAGTTTTGGAATAGATGGAGGAATCAGAAAAGTAGCAATGATAGCA     358
DSM-29675         CGTAAATTTAATTCTAGTTTTGGAATAGATGGAGGAATCAGAAAAGTAGCAATGATAGCA     358
DSM-29676         CGTAAATTTAATTCTAGTTTTGGAATAGATGGAGGAATCAGAAAAGTAGCAATGATAGCA     358
DSM-29677         CGTAAATTTAATTCTAGTTTTGGAATAGATGGAGGAATCAGAAAAGTAGCAATGATAGCA     358
DSM-29678         CGTAAATTTAATTCTAGTTTTGGAATAGATGGAGGAATCAGAAAAGTAGCAATGATAGCA     358
DSM-29679         CGTAAATTTAATTCTAGTTTTGGAATAGATGGAGGAATCAGAAAAGTAGCAATGATAGCA     358
DSM-29683         CGTAAATTTAATTCTAGTTTTGGAATAGATGGAGGAATCAGAAAAGTAGCAATGATAGCA     358
DSM-29684         CGTAAATTTAATTCTAGTTTTGGAATAGATGGAGGAATCAGAAAAGTAGCAATGATAGCA     358
DSM-29685         CGTAAATTTAATTCTAGTTTTGGAATAGATGGAGGAATCAGAAAAGTAGCAATGATAGCA     358
DSM-29686         CGTAAATTTAATTCTAGTTTTGGAATAGATGGAGGAATCAGAAAAGTAGCAATGATAGCA     358
DSM-29089         CGTAAATTTAATTCTAGTTTTGGAATAGATGGAGGAATCAGAAAAGTAGCAATGATAGCA     358
DSM-29689         CGTAAATTTAATTCTAGTTTTGGAATAGATGGAGGAATCAGAAAAGTAGCAATGATAGCA     358
DSM-29692         CGTAAATTTAATTCTAGTTTTGGAATAGATGGAGGAATCAGAAAAGTAGCAATGATAGCA     358
DSM-29091         CGTAAATTTAATTCTAGTTTTGGAATAGATGGAGGAATCAGAAAAGTAGCAATGATAGCA     358
630               CGTAAATTTAATTCTAGTTTTGGAATAGATGGAGGAATCAGAAAAGTAGCAATGATAGCA     358
630-delta-erm     CGTAAATTTAATTCTAGTTTTGGAATAGATGGAGGAATCAGAAAAGTAGCAATGATAGCA     358
DSM-29744         CGTAAATTTAATTCTAGTTTTGGAATAGATGGAGGAATCAGAAAAGTAGCAATGATAGCA     358
DSM-29745         CGTAAATTTAATTCTAGTTTTGGAATAGATGGAGGAATCAGAAAAGTAGCAATGATAGCA     358
DSM-29754         CGTAAATTTAATTCTAGTTTTGGAATAGATGGAGGAATCAGAAAAGTAGCAATGATAGCA     358
DSM-29687         CGTAAATTTAATTCTAGTTTTGGAATAGATGGAGGAATCAGAAAAGTAGCAATGATAGCA     358
DSM-29690         CGTAAATTTAATTCTAGTTTTGGAATAGATGGAGGAATCAGAAAAGTAGCAATGATAGCA     358
DSM-29691         CGTAAATTTAATTCTAGTTTTGGAATAGATGGAGGAATCAGAAAAGTAGCAATGATAGCA     358
DSM-29092         CGTAAATTTAATTCTAGTTTTGGAATAGATGGAGGAATCAGAAAAGTAGCAATGATAGCA     358
DSM-29093         CGTAAATTTAATTCTAGTTTTGGAATAGATGGAGGAATCAGAAAAGTAGCAATGATAGCA     358
DSM-29756         CGTAAATTTAATTCTAGTTTTGGAATAGATGGAGGAATCAGAAAAGTAGCAATGATAGCA     358
DSM-29094         CGTAAATTTAATTCTAGTTTTGGAATAGATGGAGGAATCAGAAAAGTAGCAATGATAGCA     358
2007855           CGTAAATTTAATTCTAGTTTTGGAATAGATGGAGGAATCAGAAAAGTAGCAATGATAGCA     358
BI1               CGTAAATTTAATTCTAGTTTTGGAATAGATGGAGGAATCAGAAAAGTAGCAATGATAGCA     358
DSM-28196         CGTAAATTTAATTCTAGTTTTGGAATAGATGGAGGAATCAGAAAAGTAGCAATGATAGCA     358
DSM-27638         CGTAAATTTAATTCTAGTTTTGGAATAGATGGAGGAATCAGAAAAGTAGCAATGATAGCA     358
DSM-27640         CGTAAATTTAATTCTAGTTTTGGAATAGATGGAGGAATCAGAAAAGTAGCAATGATAGCA     358
DSM-29680         CGTAAATTTAATTCTAGTTTTGGAATAGATGGAGGAATCAGAAAAGTAGCAATGATAGCA     358
R20291            CGTAAATTTAATTCTAGTTTTGGAATAGATGGAGGAATCAGAAAAGTAGCAATGATAGCA     358
DSM-29747         CGTAAATTTAATTCTAGTTTTGGAATAGATGGAGGAATCAGAAAAGTAGCAATGATAGCA     358
DSM-29752         CGTAAATTTAATTCTAGTTTTGGAATAGATGGAGGAATCAGAAAAGTAGCAATGATAGCA     358
DSM-29753         CGTAAATTTAATTCTAGTTTTGGAATAGATGGAGGAATCAGAAAAGTAGCAATGATAGCA     358
M120              CGTAAATTTAATTCTAGTTTTGGAATAGATGGAGGAATCAGAAAAGTAGCAATGATAGCA     358
                  ************************************************************
```

```
M68              TGTATATTTTTTTTATCAGTAGTTGACATTCTTACAAAGTTTAACTTTTTATTTATGTTA       418
CF5              TGTATATTTTTTTTATCAGTAGTTGACATTCTTACAAAGTTTAACTTTTTATTTATGTTA       418
DSM-29095        TGTATATTTTTTTTATCAGTAGTTGACATTCTTACAAAGTTTAACTTTTTATTTATGTTA       418
DSM-29090        TGTATATTTTTTTTATCAGTAGTTGACATTCTTACAAAGTTTAACTTTTTATTTATGTTA       418
DSM-28194        TGTATATTTTTTTTATCAGTAGTTGACATTCTTACAAAGTTTAACTTTTTATTTATGTTA       418
DSM-28197        TGTATATTTTTTTTATCAGTAGTTGACATTCTTACAAAGTTTAACTTTTTATTTATGTTA       418
DSM-1296T        TGTATATTTTTTTTATCAGTAGTTGACATTCTTACAAAGTTTAACTTTTTATTTATGTTA       418
DSM-27639        TGTATATTTTTTTTATCAGTAGTTGACATTCTTACAAAGTTTAACTTTTTATTTATGTTA       418
DSM-29675        TGTATATTTTTTTTATCAGTAGTTGACATTCTTACAAAGTTTAACTTTTTATTTATGTTA       418
DSM-29676        TGTATATTTTTTTTATCAGTAGTTGACATTCTTACAAAGTTTAACTTTTTATTTATGTTA       418
DSM-29677        TGTATATTTTTTTTATCAGTAGTTGACATTCTTACAAAGTTTAACTTTTTATTTATGTTA       418
DSM-29678        TGTATATTTTTTTTATCAGTAGTTGACATTCTTACAAAGTTTAACTTTTTATTTATGTTA       418
DSM-29679        TGTATATTTTTTTTATCAGTAGTTGACATTCTTACAAAGTTTAACTTTTTATTTATGTTA       418
DSM-29683        TGTATATTTTTTTTATCAGTAGTTGACATTCTTACAAAGTTTAACTTTTTATTTATGTTA       418
DSM-29684        TGTATATTTTTTTTATCAGTAGTTGACATTCTTACAAAGTTTAACTTTTTATTTATGTTA       418
DSM-29685        TGTATATTTTTTTTATCAGTAGTTGACATTCTTACAAAGTTTAACTTTTTATTTATGTTA       418
DSM-29686        TGTATATTTTTTTTATCAGTAGTTGACATTCTTACAAAGTTTAACTTTTTATTTATGTTA       418
DSM-29089        TGTATATTTTTTTTATCAGTAGTTGACATTCTTACAAAGTTTAACTTTTTATTTATGTTA       418
DSM-29689        TGTATATTTTTTTTATCAGTAGTTGACATTCTTACAAAGTTTAACTTTTTATTTATGTTA       418
DSM-29692        TGTATATTTTTTTTATCAGTAGTTGACATTCTTACAAAGTTTAACTTTTTATTTATGTTA       418
DSM-29091        TGTATATTTTTTTTATCAGTAGTTGACATTCTTACAAAGTTTAACTTTTTATTTATGTTA       418
630              TGTATATTTTTTTTATCAGTAGTTGACATTCTTACAAAGTTTAACTTTTTATTTATGTTA       418
630-delta-erm    TGTATATTTTTTTTATCAGTAGTTGACATTCTTACAAAGTTTAACTTTTTATTTATGTTA       418
DSM-29744        TGTATATTTTTTTTATCAGTAGTTGACATTCTTACAAAGTTTAACTTTTTATTTATGTTA       418
DSM-29745        TGTATATTTTTTTTATCAGTAGTTGACATTCTTACAAAGTTTAACTTTTTATTTATGTTA       418
DSM-29754        TGTATATTTTTTTTATCAGTAGTTGACATTCTTACAAAGTTTAACTTTTTATTTATGTTA       418
DSM-29687        TGTATATTTTTTTTATCAGTAGTTGACATTCTTACAAAGTTTAACTTTTTATTTATGTTA       418
DSM-29690        TGTATATTTTTTTTATCAGTAGTTGACATTCTTACAAAGTTTAACTTTTTATTTATGTTA       418
DSM-29691        TGTATATTTTTTTTATCAGTAGTTGACATTCTTACAAAGTTTAACTTTTTATTTATGTTA       418
DSM-29092        TGTATATTTTTTTTATCAGTAGTTGACATTCTTACAAAGTTTAACTTTTTATTTATGTTA       418
DSM-29093        TGTATATTTTTTTTATCAGTAGTTGACATTCTTACAAAGTTTAACTTTTTATTTATGTTA       418
DSM-29756        TGTATATTTTTTTTATCAGTAGTTGACATTCTTACAAAGTTTAACTTTTTATTTATGTTA       418
DSM-29094        TGTATATTTTTTTTATCAGTAGTTGACATTCTTACAAAGTTTAACTTTTTATTTATGTTA       418
2007855          TGTATATTTTTTTTATCAGTAGTTGACATTCTTACAAAGTTTAACTTTTTATTTATGTTA       418
BI1              TGTATATTTTTTTTATCAGTAGTTGACATTCTTACAAAGTTTAACTTTTTATTTATGTTA       418
DSM-28196        TGTATATTTTTTTTATCAGTAGTTGACATTCTTACAAAGTTTAACTTTTTATTTATGTTA       418
DSM-27638        TGTATATTTTTTTTATCAGTAGTTGACATTCTTACAAAGTTTAACTTTTTATTTATGTTA       418
DSM-27640        TGTATATTTTTTTTATCAGTAGTTGACATTCTTACAAAGTTTAACTTTTTATTTATGTTA       418
DSM-29680        TGTATATTTTTTTTATCAGTAGTTGACATTCTTACAAAGTTTAACTTTTTATTTATGTTA       418
R20291           TGTATATTTTTTTTATCAGTAGTTGACATTCTTACAAAGTTTAACTTTTTATTTATGTTA       418
DSM-29747        TGTATATTTTTTTTATCAGTAGTTGACATTCTTACAAAGTTTAACTTTTTATTTATGTTA       418
DSM-29752        TGTATATTTTTTTTATCAGTAGTTGACATTCTTACAAAGTTTAACTTTTTATTTATGTTA       418
DSM-29753        TGTATATTTTTTTTATCAGTAGTTGACATTCTTACAAAGTTTAACTTTTTATTTATGTTA       418
M120             TGTATATTTTTTTTATCAGTAGTTGACATTCTTACAAAGTTTAACTTTTTATTTATGTTA       418
                 ************************************************************
```

M68	CCACAAGATTGTATCAATTTTTTAAGACTAAAACATCTTGGAATATCTGAATTTTTCTCT	478
CF5	CCACAAGATTGTATCAATTTTTTAAGACTAAAACATCTTGGAATATCTGAATTTTTCTCT	478
DSM-29095	CCACAAGATTGTATCAATTTTTTAAGACTAAAACATCTTGGAATATCTGAATTTTTCTCT	478
DSM-29090	CCACAAGATTGTATCAATTTTTTAAGACTAAAACATCTTGGAATATCTGAATTTTTCTCT	478
DSM-28194	CCACAAGATTGTATCAATTTTTTAAGACTAAAACATCTTGGAATATCTGAATTTTTCTCT	478
DSM-28197	CCACAAGATTGTATCAATTTTTTAAGACTAAAACATCTTGGAATATCTGAATTTTTCTCT	478
DSM-1296T	CCACAAGATTGTATCAATTTTTTAAGACTAAAACATCTTGGAATATCTGAATTTTTCTCT	478
DSM-27639	CCACAAGATTGTATCAATTTTTTAAGACTAAAACATCTTGGAATATCTGAATTTTTCTCT	478
DSM-29675	CCACAAGATTGTATCAATTTTTTAAGACTAAAACATCTTGGAATATCTGAATTTTTCTCT	478
DSM-29676	CCACAAGATTGTATCAATTTTTTAAGACTAAAACATCTTGGAATATCTGAATTTTTCTCT	478
DSM-29677	CCACAAGATTGTATCAATTTTTTAAGACTAAAACATCTTGGAATATCTGAATTTTTCTCT	478
DSM-29678	CCACAAGATTGTATCAATTTTTTAAGACTAAAACATCTTGGAATATCTGAATTTTTCTCT	478
DSM-29679	CCACAAGATTGTATCAATTTTTTAAGACTAAAACATCTTGGAATATCTGAATTTTTCTCT	478
DSM-29683	CCACAAGATTGTATCAATTTTTTAAGACTAAAACATCTTGGAATATCTGAATTTTTCTCT	478
DSM-29684	CCACAAGATTGTATCAATTTTTTAAGACTAAAACATCTTGGAATATCTGAATTTTTCTCT	478
DSM-29685	CCACAAGATTGTATCAATTTTTTAAGACTAAAACATCTTGGAATATCTGAATTTTTCTCT	478
DSM-29686	CCACAAGATTGTATCAATTTTTTAAGACTAAAACATCTTGGAATATCTGAATTTTTCTCT	478
DSM-29089	CCACAAGATTGTATCAATTTTTTAAGACTAAAACATCTTGGAATATCTGAATTTTTCTCT	478
DSM-29689	CCACAAGATTGTATCAATTTTTTAAGACTAAAACATCTTGGAATATCTGAATTTTTCTCT	478
DSM-29692	CCACAAGATTGTATCAATTTTTTAAGACTAAAACATCTTGGAATATCTGAATTTTTCTCT	478
DSM-29091	CCACAAGATTGTATCAATTTTTTAAGACTAAAACATCTTGGAATATCTGAATTTTTCTCT	478
630	CCACAAGATTGTATCAATTTTTTAAGACTAAAACATCTTGGAATATCTGAATTTTTCTCT	478
630-delta-erm	CCACAAGATTGTATCAATTTTTTAAGACTAAAACATCTTGGAATATCTGAATTTTTCTCT	478
DSM-29744	CCACAAGATTGTATCAATTTTTTAAGACTAAAACATCTTGGAATATCTGAATTTTTCTCT	478
DSM-29745	CCACAAGATTGTATCAATTTTTTAAGACTAAAACATCTTGGAATATCTGAATTTTTCTCT	478
DSM-29754	CCACAAGATTGTATCAATTTTTTAAGACTAAAACATCTTGGAATATCTGAATTTTTCTCT	478
DSM-29687	CCACAAGATTGTATCAATTTTTTAAGACTAAAACATCTTGGAATATCTGAATTTTTCTCT	478
DSM-29690	CCACAAGATTGTATCAATTTTTTAAGACTAAAACATCTTGGAATATCTGAATTTTTCTCT	478
DSM-29691	CCACAAGATTGTATCAATTTTTTAAGACTAAAACATCTTGGAATATCTGAATTTTTCTCT	478
DSM-29092	CCACAAGATTGTATCAATTTTTTAAGACTAAAACATCTTGGAATATCTGAATTTTTCTCT	478
DSM-29093	CCACAAGATTGTATCAATTTTTTAAGACTAAAACATCTTGGAATATCTGAATTTTTCTCT	478
DSM-29756	CCACAAGATTGTATCAATTTTTTAAGACTAAAACATCTTGGAATATCTGAATTTTTCTCT	478
DSM-29094	CCACAAGATTGTATCAATTTTTTAAGACTAAAACATCTTGGAATATCTGAATTTTTCTCT	478
2007855	CCACAAGATTGTATCAATTTTTTAAGACTAAAACATCTTGGAATATCTGAATTTTTCTCT	478
BI1	CCACAAGATTGTATCAATTTTTTAAGACTAAAACATCTTGGAATATCTGAATTTTTCTCT	478
DSM-28196	CCACAAGATTGTATCAATTTTTTAAGACTAAAACATCTTGGAATATCTGAATTTTTCTCT	478
DSM-27638	CCACAAGATTGTATCAATTTTTTAAGACTAAAACATCTTGGAATATCTGAATTTTTCTCT	478
DSM-27640	CCACAAGATTGTATCAATTTTTTAAGACTAAAACATCTTGGAATATCTGAATTTTTCTCT	478
DSM-29680	CCACAAGATTGTATCAATTTTTTAAGACTAAAACATCTTGGAATATCTGAATTTTTCTCT	478
R20291	CCACAAGATTGTATCAATTTTTTAAGACTAAAACATCTTGGAATATCTGAATTTTTCTCT	478
DSM-29747	CCACAAGATTGTATCAATTTTTTAAGACTAAAACATCTTGGAATATCTGAATTTTTCTCT	478
DSM-29752	CCACAAGATTGTATCAATTTTTTAAGACTAAAACATCTTGGAATATCTGAATTTTTCTCT	478
DSM-29753	CCACAAGATTGTATCAATTTTTTAAGACTAAAACATCTTGGAATATCTGAATTTTTCTCT	478
M120	CCACAAGATTGTATCAATTTTTTAAGACTAAAACATCTTGGAATATCTGAATTTTTCTCT	478
	**	

```
M68              ATTTTATTTATTTTATATGAAAGTGTAAGTATATTAAAAAATATGTGCTTATGTGGATTA    538
CF5              ATTTTATTTATTTTATATGAAAGTGTAAGTATATTAAAAAATATGTGCTTATGTGGATTA    538
DSM-29095        ATTTTATTTATTTTATATGAAAGTGTAAGTATATTAAAAAATATGTGCTTATGTGGATTA    538
DSM-29090        ATTTTATTTATTTTATATGAAAGTGTAAGTATATTAAAAAATATGTGCTTATGTGGATTA    538
DSM-28194        ATTTTATTTATTTTATATGAAAGTGTAAGTATATTAAAAAATATGTGCTTATGTGGATTA    538
DSM-28197        ATTTTATTTATTTTATATGAAAGTGTAAGTATATTAAAAAATATGTGCTTATGTGGATTA    538
DSM-1296T        ATTTTATTTATTTTATATGAAAGTGTAAGTATATTAAAAAATATGTGCTTATGTGGATTA    538
DSM-27639        ATTTTATTTATTTTATATGAAAGTGTAAGTATATTAAAAAATATGTGCTTATGTGGATTA    538
DSM-29675        ATTTTATTTATTTTATATGAAAGTGTAAGTATATTAAAAAATATGTGCTTATGTGGATTA    538
DSM-29676        ATTTTATTTATTTTATATGAAAGTGTAAGTATATTAAAAAATATGTGCTTATGTGGATTA    538
DSM-29677        ATTTTATTTATTTTATATGAAAGTGTAAGTATATTAAAAAATATGTGCTTATGTGGATTA    538
DSM-29678        ATTTTATTTATTTTATATGAAAGTGTAAGTATATTAAAAAATATGTGCTTATGTGGATTA    538
DSM-29679        ATTTTATTTATTTTATATGAAAGTGTAAGTATATTAAAAAATATGTGCTTATGTGGATTA    538
DSM-29683        ATTTTATTTATTTTATATGAAAGTGTAAGTATATTAAAAAATATGTGCTTATGTGGATTA    538
DSM-29684        ATTTTATTTATTTTATATGAAAGTGTAAGTATATTAAAAAATATGTGCTTATGTGGATTA    538
DSM-29685        ATTTTATTTATTTTATATGAAAGTGTAAGTATATTAAAAAATATGTGCTTATGTGGATTA    538
DSM-29686        ATTTTATTTATTTTATATGAAAGTGTAAGTATATTAAAAAATATGTGCTTATGTGGATTA    538
DSM-29089        ATTTTATTTATTTTATATGAAAGTGTAAGTATATTAAAAAATATGTGCTTATGTGGATTA    538
DSM-29689        ATTTTATTTATTTTATATGAAAGTGTAAGTATATTAAAAAATATGTGCTTATGTGGATTA    538
DSM-29692        ATTTTATTTATTTTATATGAAAGTGTAAGTATATTAAAAAATATGTGCTTATGTGGATTA    538
DSM-29091        ATTTTATTTATTTTATATGAAAGTGTAAGTATATTAAAAAATATGTGCTTATGTGGATTA    538
630              ATTTTATTTATTTTATATGAAAGTGTAAGTATATTAAAAAATATGTGCTTATGTGGATTA    538
630-delta-erm    ATTTTATTTATTTTATATGAAAGTGTAAGTATATTAAAAAATATGTGCTTATGTGGATTA    538
DSM-29744        ATTTTATTTATTTTATATGAAAGTGTAAGTATATTAAAAAATATGTGCTTATGTGGATTA    538
DSM-29745        ATTTTATTTATTTTATATGAAAGTGTAAGTATATTAAAAAATATGTGCTTATGTGGATTA    538
DSM-29754        ATTTTATTTATTTTATATGAAAGTGTAAGTATATTAAAAAATATGTGCTTATGTGGATTA    538
DSM-29687        ATTTTATTTATTTTATATGAAAGTGTAAGTATATTAAAAAATATGTGCTTATGTGGATTA    538
DSM-29690        ATTTTATTTATTTTATATGAAAGTGTAAGTATATTAAAAAATATGTGCTTATGTGGATTA    538
DSM-29691        ATTTTATTTATTTTATATGAAAGTGTAAGTATATTAAAAAATATGTGCTTATGTGGATTA    538
DSM-29092        ATTTTATTTATTTTATATGAAAGTGTAAGTATATTAAAAAATATGTGCTTATGTGGATTA    538
DSM-29093        ATTTTATTTATTTTATATGAAAGTGTAAGTATATTAAAAAATATGTGCTTATGTGGATTA    538
DSM-29756        ATTTTATTTATTTTATATGAAAGTGTAAGTATATTAAAAAATATGTGCTTATGTGGATTA    538
DSM-29094        ATTTTATTTATTTTATATGAAAGTGTAAGTATATTAAAAAATATGTGCTTATGTGGATTA    538
2007855          ATTTTATTTATTTTATATGAAAGTGTAAGCATATTAAAAAATATGTGCTTATGTGGATTA    538
BI1              ATTTTATTTATTTTATATGAAAGTGTAAGCATATTAAAAAATATGTGCTTATGTGGATTA    538
DSM-28196        ATTTTATTTATTTTATATGAAAGTGTAAGCATATTAAAAAATATGTGCTTATGTGGATTA    538
DSM-27638        ATTTTATTTATTTTATATGAAAGTGTAAGCATATTAAAAAATATGTGCTTATGTGGATTA    538
DSM-27640        ATTTTATTTATTTTATATGAAAGTGTAAGCATATTAAAAAATATGTGCTTATGTGGATTA    538
DSM-29680        ATTTTATTTATTTTATATGAAAGTGTAAGCATATTAAAAAATATGTGCTTATGTGGATTA    538
R20291           ATTTTATTTATTTTATATGAAAGTGTAAGCATATTAAAAAATATGTGCTTATGTGGATTA    538
DSM-29747        ATTTTATTTATTTTATATGAAAGTGTAAGTATATTAAAAAATATGTGCTTATGTGGATTA    538
DSM-29752        ATTTTATTTATTTTATATGAAAGTGTAAGTATATTAAAAAATATGTGCTTATGTGGATTA    538
DSM-29753        ATTTTATTTATTTTATATGAAAGTGTAAGTATATTAAAAAATATGTGCTTATGTGGATTA    538
M120             ATTTTATTTATTTTATATGAAAGTGTAAGTATATTAAAAAATATGTGCTTATGTGGATTA    538
                 ***************************** ******************************
```

```
M68                CCAGTACCTAAGAGATTAAAGGAAAAAATAGCAGTTTTACTAGATGCAATGACAGATGAA     598
CF5                CCAGTACCTAAGAGATTAAAGGAAAAAATAGCAGTTTTACTAGATGCAATGACAGATGAA     598
DSM-29095          CCAGTACCTAAGAGATTAAAGGAAAAAATAGCAATTTTACTAGATGCAATGACAGATGAA     598
DSM-29090          CCAGTACCTAAGAGATTAAAGGAAAAAATAGCAATTTTACTAGATGCAATGACAGATGAA     598
DSM-28194          CCAGTACCTAAGAGATTAAAGGAAAAAATAGCAATTTTACTAGATGCAATGACAGATGAA     598
DSM-28197          CCAGTACCTAAGAGATTAAAGGAAAAAATAGCAATTTTACTAGATGCAATGACAGATGAA     598
DSM-1296T          CCAGTACCTAAGAGATTAAAGGAAAAAATAGCAATTTTACTAGATGCAATGACAGATGAA     598
DSM-27639          CCAGTACCTAAGAGATTAAAGGAAAAAATAGCAATTTTACTAGATGCAATGACAGATGAA     598
DSM-29675          CCAGTACCTAAGAGATTAAAGGAAAAAATAGCAATTTTACTAGATGCAATGACAGATGAA     598
DSM-29676          CCAGTACCTAAGAGATTAAAGGAAAAAATAGCAATTTTACTAGATGCAATGACAGATGAA     598
DSM-29677          CCAGTACCTAAGAGATTAAAGGAAAAAATAGCAATTTTACTAGATGCAATGACAGATGAA     598
DSM-29678          CCAGTACCTAAGAGATTAAAGGAAAAAATAGCAATTTTACTAGATGCAATGACAGATGAA     598
DSM-29679          CCAGTACCTAAGAGATTAAAGGAAAAAATAGCAATTTTACTAGATGCAATGACAGATGAA     598
DSM-29683          CCAGTACCTAAGAGATTAAAGGAAAAAATAGCAATTTTACTAGATGCAATGACAGATGAA     598
DSM-29684          CCAGTACCTAAGAGATTAAAGGAAAAAATAGCAATTTTACTAGATGCAATGACAGATGAA     598
DSM-29685          CCAGTACCTAAGAGATTAAAGGAAAAAATAGCAATTTTACTAGATGCAATGACAGATGAA     598
DSM-29686          CCAGTACCTAAGAGATTAAAGGAAAAAATAGCAATTTTACTAGATGCAATGACAGATGAA     598
DSM-29089          CCAGTACCTAAGAGATTAAAGGAAAAAATAGCAATTTTACTAGATGCAATGACAGATGAA     598
DSM-29689          CCAGTACCTAAGAGATTAAAGGAAAAAATAGCAATTTTACTAGATGCAATGACAGATGAA     598
DSM-29692          CCAGTACCTAAGAGATTAAAGGAAAAAATAGCAATTTTACTAGATGCAATGACAGATGAA     598
DSM-29091          CCAGTACCTAAGAGATTAAAGGAAAAAATAGCAATTTTACTAGATGCAATGACAGATGAA     598
630                CCAGTACCTAAGAGATTAAAGGAAAAAATAGCAATTTTACTAGATGCAATGACAGATGAA     598
630-delta-erm      CCAGTACCTAAGAGATTAAAGGAAAAAATAGCAATTTTACTAGATGCAATGACAGATGAA     598
DSM-29744          CCAGTACCTAAGAGATTAAAGGAAAAAATAGCAATTTTACTAGATGCAATGACAGATGAA     598
DSM-29745          CCAGTACCTAAGAGATTAAAGGAAAAAATAGCAATTTTACTAGATGCAATGACAGATGAA     598
DSM-29754          CCAGTACCTAAGAGATTAAAGGAAAAAATAGCAATTTTACTAGATGCAATGACAGATGAA     598
DSM-29687          CCAGTACCTAAGAGATTAAAGGAAAAAATAGCAATTTTACTAGATGCAATGACAGATGAA     598
DSM-29690          CCAGTACCTAAGAGATTAAAGGAAAAAATAGCAATTTTACTAGATGCAATGACAGATGAA     598
DSM-29691          CCAGTACCTAAGAGATTAAAGGAAAAAATAGCAATTTTACTAGATGCAATGACAGATGAA     598
DSM-29092          CCAGTACCTAAGAGATTAAAGGAAAAAATAGCAATTTTACTAGATGCAATGACAGATGAA     598
DSM-29093          CCAGTACCTAAGAGATTAAAGGAAAAAATAGCAATTTTACTAGATGCAATGACAGATGAA     598
DSM-29756          CCAGTACCTAAGAGATTAAAGGAAAAAATAGCAATTTTACTAGATGCAATGACAGATGAA     598
DSM-29094          CCAGTACCTAAGAGATTAAAGGAAAAAATAGCAATTTTACTAGATGCAATGACAGATGAA     598
2007855            CCAGTACCTAAGAGATTAAAGGAAAAAATAGCAGTTTTACTAGATGCAATGACAGATGAA     598
BI1                CCAGTACCTAAGAGATTAAAGGAAAAAATAGCAGTTTTACTAGATGCAATGACAGATGAA     598
DSM-28196          CCAGTACCTAAGAGATTAAAGGAAAAAATAGCAGTTTTACTAGATGCAATGACAGATGAA     598
DSM-27638          CCAGTACCTAAGAGATTAAAGGAAAAAATAGCAGTTTTACTAGATGCAATGACAGATGAA     598
DSM-27640          CCAGTACCTAAGAGATTAAAGGAAAAAATAGCAGTTTTACTAGATGCAATGACAGATGAA     598
DSM-29680          CCAGTACCTAAGAGATTAAAGGAAAAAATAGCAGTTTTACTAGATGCAATGACAGATGAA     598
R20291             CCAGTACCTAAGAGATTAAAGGAAAAAATAGCAGTTTTACTAGATGCAATGACAGATGAA     598
DSM-29747          CCAGTACCTAAGAGATTAAAGGAAAAAATAGCAGTTTTACTAGATGCAATGACAGATGAA     598
DSM-29752          CCAGTACCTAAGAGATTAAAGGAAAAAATAGCAGTTTTACTAGATGCAATGACAGATGAA     598
DSM-29753          CCAGTACCTAAGAGATTAAAGGAAAAAATAGCAGTTTTACTAGATGCAATGACAGATGAA     598
M120               CCAGTACCTAAGAGATTAAAGGAAAAAATAGCAGTTTTACTAGATGCAATGACAGATGAA     598
                   ********************************* **************************
```

```
M68             ATGAATGCTAAGGATGAAAAGTAAGTAATGGTAGATATAATAAAGATATTAACAAATAAA    658
CF5             ATGAATGCTAAGGATGAAAAGTAAGTAATGGTAGATATAATAAAGATATTAACAAATAAA    658
DSM-29095       ATGAATGCTAAGGATGAAAAGTAAGTAATGGTAGATATAATAAAGATATTAACAAATAAA    658
DSM-29090       ATGAATGCTAAGGATGAAAAGTAAGTAATGGTAGATATAATAAAGATATTAACAAATAAA    658
DSM-28194       ATGAATGCTAAGGATGAAAAGTAAGTAATGGTAGATATAATAAAGATATTAACAAATAAA    658
DSM-28197       ATGAATGCTAAGGATGAAAAGTAAGTAATGGTAGATATAATAAAGATATTAACAAATAAA    658
DSM-1296T       ATGAATGCTAAGGATGAAAAGTAAGTAATGGTAGATATAATAAAGATATTAACAAATAAA    658
DSM-27639       ATGAATGCTAAGGATGAAAAGTAAGTAATGGTAGATATAATAAAGATATTAACAAATAAA    658
DSM-29675       ATGAATGCTAAGGATGAAAAGTAAGTAATGGTAGATATAATAAAGATATTAACAAATAAA    658
DSM-29676       ATGAATGCTAAGGATGAAAAGTAAGTAATGGTAGATATAATAAAGATATTAACAAATAAA    658
DSM-29677       ATGAATGCTAAGGATGAAAAGTAAGTAATGGTAGATATAATAAAGATATTAACAAATAAA    658
DSM-29678       ATGAATGCTAAGGATGAAAAGTAAGTAATGGTAGATATAATAAAGATATTAACAAATAAA    658
DSM-29679       ATGAATGCTAAGGATGAAAAGTAAGTAATGGTAGATATAATAAAGATATTAACAAATAAA    658
DSM-29683       ATGAATGCTAAGGATGAAAAGTAAGTAATGGTAGATATAATAAAGATATTAACAAATAAA    658
DSM-29684       ATGAATGCTAAGGATGAAAAGTAAGTAATGGTAGATATAATAAAGATATTAACAAATAAA    658
DSM-29685       ATGAATGCTAAGGATGAAAAGTAAGTAATGGTAGATATAATAAAGATATTAACAAATAAA    658
DSM-29686       ATGAATGCTAAGGATGAAAAGTAAGTAATGGTAGATATAATAAAGATATTAACAAATAAA    658
DSM-29089       ATGAATGCTAAGGATGAAAAGTAAGTAATGGTAGATATAATAAAGATATTAACAAATAAA    658
DSM-29689       ATGAATGCTAAGGATGAAAAGTAAGTAATGGTAGATATAATAAAGATATTAACAAATAAA    658
DSM-29692       ATGAATGCTAAGGATGAAAAGTAAGTAATGGTAGATATAATAAAGATATTAACAAATAAA    658
DSM-29091       ATGAATGCTAAGGATGAAAAGTAAGTAATGGTAGATATAATAAAGATATTAACAAATAAA    658
630             ATGAATGCTAAGGATGAAAAGTAAGTAATGGTAGATATAATAAAGATATTAACAAATAAA    658
630-delta-erm   ATGAATGCTAAGGATGAAAAGTAAGTAATGGTAGATATAATAAAGATATTAACAAATAAA    658
DSM-29744       ATGAATGCTAAGGATGAAAAGTAAGTAATGGTAGATATAATAAAGATATTAACAAATAAA    658
DSM-29745       ATGAATGCTAAGGATGAAAAGTAAGTAATGGTAGATATAATAAAGATATTAACAAATAAA    658
DSM-29754       ATGAATGCTAAGGATGAAAAGTAAGTAATGGTAGATATAATAAAGATATTAACAAATAAA    658
DSM-29687       ATGAATGCTAAGGATGAAAAGTAAGTAATGGTAGATATAATAAAGATATTAACAAATAAA    658
DSM-29690       ATGAATGCTAAGGATGAAAAGTAAGTAATGGTAGATATAATAAAGATATTAACAAATAAA    658
DSM-29691       ATGAATGCTAAGGATGAAAAGTAAGTAATGGTAGATATAATAAAGATATTAACAAATAAA    658
DSM-29092       ATGAATGCTAAGGATGAAAAGTAAGTAATGGTAGATATAATAAAGATATTAACAAATAAA    658
DSM-29093       ATGAATGCTAAGGATGAAAAGTAAGTAATGGTAGATATAATAAAGATATTAACAAATAAA    658
DSM-29756       ATGAATGCTAAGGATGAAAAGTAAGTAATGGTAGATATAATAAAGATATTAACAAATAAA    658
DSM-29094       ATGAATGCTAAGGATGAAAAGTAAGTAATGGTAGATATAATAAAGATATTAACAAATAAA    658
2007855         ATGAATGCTAAGGATGAAAAGTAAGTAATGGTAGATATAATAAAAATATTAACAAATAAA    658
BI1             ATGAATGCTAAGGATGAAAAGTAAGTAATGGTAGATATAATAAAAATATTAACAAATAAA    658
DSM-28196       ATGAATGCTAAGGATGAAAAGTAAGTAATGGTAGATATAATAAAAATATTAACAAATAAA    658
DSM-27638       ATGAATGCTAAGGATGAAAAGTAAGTAATGGTAGATATAATAAAAATATTAACAAATAAA    658
DSM-27640       ATGAATGCTAAGGATGAAAAGTAAGTAATGGTAGATATAATAAAAATATTAACAAATAAA    658
DSM-29680       ATGAATGCTAAGGATGAAAAGTAAGTAATGGTAGATATAATAAAAATATTAACAAATAAA    658
R20291          ATGAATGCTAAGGATGAAAAGTAAGTAATGGTAGATATAATAAAAATATTAACAAATAAA    658
DSM-29747       ATGAATGCTAAGGATGAAAAGTAAGTACTGGTAGATATAATAAAGATATTAACAAATAAA    658
DSM-29752       ATGAATGCTAAGGATGAAAAGTAAGTACTGGTAGATATAATAAAGATATTAACAAATAAA    658
DSM-29753       ATGAATGCTAAGGATGAAAAGTAAGTACTGGTAGATATAATAAAGATATTAACAAATAAA    658
M120            ATGAATGCTAAGGATGAAAAGTAAGTACTGGTAGATATAATAAAGATATTAACAAATAAA    658
                *************************** **************** ***************
```

tcdE translational stop

M68	AAGTGTTATCCAAATAAGAATAGCTGAAAGTTATCATAATTCATGAAACTAATAATGAAA	718
CF5	AAGTGTTATCCAAATAAGAATAGCTGAAAGTTATCATAATTCATGAAACTAATAATGAAA	718
DSM-29095	AAGTGTTATCCAAATAAGAATAGCTGAAAGTTATCATAATTCATGAAACTAATAATGAAA	718
DSM-29090	AAGTGTTATCCAAATAAGAATAGCTGAAAGTTATCATAATTCATGAAACTAATAATGAAA	718
DSM-28194	AAGTGTTATCCAAATAAGAATAGCTGAAAGTTATCATAATTCATGAAACTAATAATGAAA	718
DSM-28197	AAGTGTTATCCAAATAAGAATAGCTGAAAGTTATCATAATTCATGAAACTAATAATGAAA	718
DSM-1296T	AAGTGTTATCCAAATAAGAATAGCTGAAAGTTATCATAATTCATGAAACTAATAATGAAA	718
DSM-27639	AAGTGTTATCCAAATAAGAATAGCTGAAAGTTATCATAATTCATGAAACTAATAATGAAA	718
DSM-29675	AAGTGTTATCCAAATAAGAATAGCTGAAAGTTATCATAATTCATGAAACTAATAATGAAA	718
DSM-29676	AAGTGTTATCCAAATAAGAATAGCTGAAAGTTATCATAATTCATGAAACTAATAATGAAA	718
DSM-29677	AAGTGTTATCCAAATAAGAATAGCTGAAAGTTATCATAATTCATGAAACTAATAATGAAA	718
DSM-29678	AAGTGTTATCCAAATAAGAATAGCTGAAAGTTATCATAATTCATGAAACTAATAATGAAA	718
DSM-29679	AAGTGTTATCCAAATAAGAATAGCTGAAAGTTATCATAATTCATGAAACTAATAATGAAA	718
DSM-29683	AAGTGTTATCCAAATAAGAATAGCTGAAAGTTATCATAATTCATGAAACTAATAATGAAA	718
DSM-29684	AAGTGTTATCCAAATAAGAATAGCTGAAAGTTATCATAATTCATGAAACTAATAATGAAA	718
DSM-29685	AAGTGTTATCCAAATAAGAATAGCTGAAAGTTATCATAATTCATGAAACTAATAATGAAA	718
DSM-29686	AAGTGTTATCCAAATAAGAATAGCTGAAAGTTATCATAATTCATGAAACTAATAATGAAA	718
DSM-29089	AAGTGTTATCCAAATAAGAATAGCTGAAAGTTATCATAATTCATGAAACTAATAATGAAA	718
DSM-29689	AAGTGTTATCCAAATAAGAATAGCTGAAAGTTATCATAATTCATGAAACTAATAATGAAA	718
DSM-29692	AAGTGTTATCCAAATAAGAATAGCTGAAAGTTATCATAATTCATGAAACTAATAATGAAA	718
DSM-29091	AAGTGTTATCCAAATAAGAATAGCTGAAAGTTATCATAATTCATGAAACTAATAATGAAA	718
630	AAGTGTTATCCAAATAAGAATAGCTGAAAGTTATCATAATTCATGAAACTAATAATGAAA	718
630-delta-erm	AAGTGTTATCCAAATAAGAATAGCTGAAAGTTATCATAATTCATGAAACTAATAATGAAA	718
DSM-29744	AAGTGTTATCCAAATAAGAATAGCTGAAAGTTATCATAATTCATGAAACTAATAATGAAA	718
DSM-29745	AAGTGTTATCCAAATAAGAATAGCTGAAAGTTATCATAATTCATGAAACTAATAATGAAA	718
DSM-29754	AAGTGTTATCCAAATAAGAATAGCTGAAAGTTATCATAATTCATGAAACTAATAATGAAA	718
DSM-29687	AAGTGTTATCCAAATAAGAATAGCTGAAAGTTATCATAATTCATGAAACTAATAATGAAA	718
DSM-29690	AAGTGTTATCCAAATAAGAATAGCTGAAAGTTATCATAATTCATGAAACTAATAATGAAA	718
DSM-29691	AAGTGTTATCCAAATAAGAATAGCTGAAAGTTATCATAATTCATGAAACTAATAATGAAA	718
DSM-29092	AAGTGTTATCCAAATAAGAATAGCTGAAAGTTATCATAATTCATGAAACTAATAATGAAA	718
DSM-29093	AAGTGTTATCCAAATAAGAATAGCTGAAAGTTATCATAATTCATGAAACTAATAATGAAA	718
DSM-29756	AAGTGTTATCCAAATAAGAATAGCTGAAAGTTATCATAATTCATGAAACTAATAATGAAA	718
DSM-29094	AAGTGTTATCCAAATAAGAATAGCTGAAAGTTATCATAATTCATGAAACTAATAATGAAA	718
2007855	AAGTGTTATCCAAATAAGAATAGCTGAAAGTTATCATAATTCATGAAACT**G**ATAATGAAA	718
BI1	AAGTGTTATCCAAATAAGAATAGCTGAAAGTTATCATAATTCATGAAACT**G**ATAATGAAA	718
DSM-28196	AAGTGTTATCCAAATAAGAATAGCTGAAAGTTATCATAATTCATGAAACT**G**ATAATGAAA	718
DSM-27638	AAGTGTTATCCAAATAAGAATAGCTGAAAGTTATCATAATTCATGAAACT**G**ATAATGAAA	718
DSM-27640	AAGTGTTATCCAAATAAGAATAGCTGAAAGTTATCATAATTCATGAAACT**G**ATAATGAAA	718
DSM-29680	AAGTGTTATCCAAATAAGAATAGCTGAAAGTTATCATAATTCATGAAACT**G**ATAATGAAA	718
R20291	AAGTGTTATCCAAATAAGAATAGCTGAAAGTTATCATAATTCATGAAACT**G**ATAATGAAA	718
DSM-29747	AAGTGTTATCCAAATAAGAATAGCTGAAAGTTATCATAATTCATGAAACT**G**ATAATGAAA	718
DSM-29752	AAGTGTTATCCAAATAAGAATAGCTGAAAGTTATCATAATTCATGAAACT**G**ATAATGAAA	718
DSM-29753	AAGTGTTATCCAAATAAGAATAGCTGAAAGTTATCATAATTCATGAAACT**G**ATAATGAAA	718
M120	AAGTGTTATCCAAATAAGAATAGCTGAAAGTTATCATAATTCATGAAACT**G**ATAATGAAA	718
	** *********	

```
M68             ACGAGGGAGCAGATGCCAAGAGACACACAAGTATTAAATACATATAATTTCGAAGCAAGT    778
CF5             ACGAGGGAGCAGATGCCAAGAGACACACAAGTATTAAATACATATAATTTCGAAGCAAGT    778
DSM-29095       ACGAGGGAGCAGATGCCAAGAGACACACAAGTATTAAATACATATAATTTCGAAGCAAGT    778
DSM-29090       ACGAGGGAGCAGATGCCAAGAGACACACAAGTATTAAATACATATAATTTCGAAGCAAGT    778
DSM-28194       ACGAGGGAGCAGATGCCAAGAGACACACAAGTATTAAATACATATAATTTCGAAGCAAGT    778
DSM-28197       ACGAGGGAGCAGATGCCAAGAGACACACAAGTATTAAATACATATAATTTCGAAGCAAGT    778
DSM-1296T       ACGAGGGAGCAGATGCCAAGAGACACACAAGTATTAAATACATATAATTTCGAAGCAAGT    778
DSM-27639       ACGAGGGAGCAGATGCCAAGAGACACACAAGTATTAAATACATATAATTTCGAAGCAAGT    778
DSM-29675       ACGAGGGAGCAGATGCCAAGAGACACACAAGTATTAAATACATATAATTTCGAAGCAAGT    778
DSM-29676       ACGAGGGAGCAGATGCCAAGAGACACACAAGTATTAAATACATATAATTTCGAAGCAAGT    778
DSM-29677       ACGAGGGAGCAGATGCCAAGAGACACACAAGTATTAAATACATATAATTTCGAAGCAAGT    778
DSM-29678       ACGAGGGAGCAGATGCCAAGAGACACACAAGTATTAAATACATATAATTTCGAAGCAAGT    778
DSM-29679       ACGAGGGAGCAGATGCCAAGAGACACACAAGTATTAAATACATATAATTTCGAAGCAAGT    778
DSM-29683       ACGAGGGAGCAGATGCCAAGAGACACACAAGTATTAAATACATATAATTTCGAAGCAAGT    778
DSM-29684       ACGAGGGAGCAGATGCCAAGAGACACACAAGTATTAAATACATATAATTTCGAAGCAAGT    778
DSM-29685       ACGAGGGAGCAGATGCCAAGAGACACACAAGTATTAAATACATATAATTTCGAAGCAAGT    778
DSM-29686       ACGAGGGAGCAGATGCCAAGAGACACACAAGTATTAAATACATATAATTTCGAAGCAAGT    778
DSM-29089       ACGAGGGAGCAGATGCCAAGAGACACACAAGTATTAAATACATATAATTTCGAAGCAAGT    778
DSM-29689       ACGAGGGAGCAGATGCCAAGAGACACACAAGTATTAAATACATATAATTTCGAAGCAAGT    778
DSM-29692       ACGAGGGAGCAGATGCCAAGAGACACACAAGTATTAAATACATATAATTTCGAAGCAAGT    778
DSM-29091       ACGAGGGAGCAGATGCCAAGAGACACACAAGTATTAAATACATATAATTTCGAAGCAAGT    778
630             ACGAGGGAGCAGATGCCAAGAGACACACAAGTATTAAATACATATAATTTCGAAGCAAGT    778
630-delta-erm   ACGAGGGAGCAGATGCCAAGAGACACACAAGTATTAAATACATATAATTTCGAAGCAAGT    778
DSM-29744       ACGAGGGAGCAGATGCCAAGAGACACACAAGTATTAAATACATATAATTTCGAAGCAAGT    778
DSM-29745       ACGAGGGAGCAGATGCCAAGAGACACACAAGTATTAAATACATATAATTTCGAAGCAAGT    778
DSM-29754       ACGAGGGAGCAGATGCCAAGAGACACACAAGTATTAAATACATATAATTTCGAAGCAAGT    778
DSM-29687       ACGAGGGAGCAGATGCCAAGAGACACACAAGTATTAAATACATATAATTTCGAAGCAAGT    778
DSM-29690       ACGAGGGAGCAGATGCCAAGAGACACACAAGTATTAAATACATATAATTTCGAAGCAAGT    778
DSM-29691       ACGAGGGAGCAGATGCCAAGAGACACACAAGTATTAAATACATATAATTTCGAAGCAAGT    778
DSM-29092       ACGAGGGAGCAGATGCCAAGAGACACACAAGTATTAAATACATATAATTTCGAAGCAAGT    778
DSM-29093       ACGAGGGAGCAGATGCCAAGAGACACACAAGTATTAAATACATATAATTTCGAAGCAAGT    778
DSM-29756       ACGAGGGAGCAGATGCCAAGAGACACACAAGTATTAAATACATATAATTTCGAAGCAAGT    778
DSM-29094       ACGAGGGAGCAGATGCCAAGAGACACACAAGTATTAAATACATATAATTTCGAAGCAAGT    778
2007855         ACGAGGGAGCAGATGCCAAGAGACACACAAGTATTAAATACATATAATTTCGAAGCAAGT    778
BI1             ACGAGGGAGCAGATGCCAAGAGACACACAAGTATTAAATACATATAATTTCGAAGCAAGT    778
DSM-28196       ACGAGGGAGCAGATGCCAAGAGACACACAAGTATTAAATACATATAATTTCGAAGCAAGT    778
DSM-27638       ACGAGGGAGCAGATGCCAAGAGACACACAAGTATTAAATACATATAATTTCGAAGCAAGT    778
DSM-27640       ACGAGGGAGCAGATGCCAAGAGACACACAAGTATTAAATACATATAATTTCGAAGCAAGT    778
DSM-29680       ACGAGGGAGCAGATGCCAAGAGACACACAAGTATTAAATACATATAATTTCGAAGCAAGT    778
R20291          ACGAGGGAGCAGATGCCAAGAGACACACAAGTATTAAATACATATAATTTCGAAGCAAGT    778
DSM-29747       ACGAGGGAGCAGATGCCAAGAGACACACAAGTATTAAATACATATAATTTCGAAGCAAGT    778
DSM-29752       ACGAGGGAGCAGATGCCAAGAGACACACAAGTATTAAATACATATAATTTCGAAGCAAGT    778
DSM-29753       ACGAGGGAGCAGATGCCAAGAGACACACAAGTATTAAATACATATAATTTCGAAGCAAGT    778
M120            ACGAGGGAGCAGATGCCAAGAGACACACAAGTATTAAATACATATAATTTCGAAGCAAGT    778
                ************************************************************
```

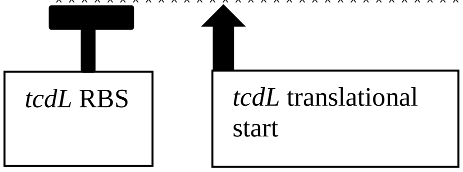

```
M68               GTTCATTACTATATGGATGACAAGGTAGTATATCAAACATTGGTTCACAAAGATGGTGCA   838
CF5               GTTCATTACTATATGGATGACAAGGTAGTATATCAAACATTGGTTCACAAAGATGGTGCA   838
DSM-29095         GTTCATTACTATATAGATGACAAGGTAGTATATCAAACATTGGTTCACAAAGATGGTGCA   838
DSM-29090         GTTCATTACTATATAGATGACAAGGTAGTATATCAAACATTGGTTCACAAAGATGGTGCA   838
DSM-28194         GTTCATTACTATATAGATGACAAGGTAGTATATCAAACATTGGTTCACAAAGATGGTGCA   838
DSM-28197         GTTCATTACTATATAGATGACAAGGTAGTATATCAAACATTGGTTCACAAAGATGGTGCA   838
DSM-1296T         GTTCATTACTATATAGATGACAAGGTAGTATATCAAACATTGGTTCACAAAGATGGTGCA   838
DSM-27639         GTTCATTACTATATAGATGACAAGGTAGTATATCAAACATTGGTTCACAAAGATGGTGCA   838
DSM-29675         GTTCATTACTATATAGATGACAAGGTAGTATATCAAACATTGGTTCACAAAGATGGTGCA   838
DSM-29676         GTTCATTACTATATAGATGACAAGGTAGTATATCAAACATTGGTTCACAAAGATGGTGCA   838
DSM-29677         GTTCATTACTATATAGATGACAAGGTAGTATATCAAACATTGGTTCACAAAGATGGTGCA   838
DSM-29678         GTTCATTACTATATAGATGACAAGGTAGTATATCAAACATTGGTTCACAAAGATGGTGCA   838
DSM-29679         GTTCATTACTATATAGATGACAAGGTAGTATATCAAACATTGGTTCACAAAGATGGTGCA   838
DSM-29683         GTTCATTACTATATAGATGACAAGGTAGTATATCAAACATTGGTTCACAAAGATGGTGCA   838
DSM-29684         GTTCATTACTATATAGATGACAAGGTAGTATATCAAACATTGGTTCACAAAGATGGTGCA   838
DSM-29685         GTTCATTACTATATAGATGACAAGGTAGTATATCAAACATTGGTTCACAAAGATGGTGCA   838
DSM-29686         GTTCATTACTATATAGATGACAAGGTAGTATATCAAACATTGGTTCACAAAGATGGTGCA   838
DSM-29089         GTTCATTACTATATAGATGACAAGGTAGTATATCAAACATTGGTTCACAAAGATGGTGCA   838
DSM-29689         GTTCATTACTATATAGATGACAAGGTAGTATATCAAACATTGGTTCACAAAGATGGTGCA   838
DSM-29692         GTTCATTACTATATAGATGACAAGGTAGTATATCAAACATTGGTTCACAAAGATGGTGCA   838
DSM-29091         GTTCATTACTATATAGATGACAAGGTAGTATATCAAACATTGGTTCACAAAGATGGTGCA   838
630               GTTCATTACTATATAGATGACAAGGTAGTATATCAAACATTGGTTCACAAAGATGGTGCA   838
630-delta-erm     GTTCATTACTATATAGATGACAAGGTAGTATATCAAACATTGGTTCACAAAGATGGTGCA   838
DSM-29744         GTTCATTACTATATAGATGACAAGGTAGTATATCAAACATTGGTTCACAAAGATGGTGCA   838
DSM-29745         GTTCATTACTATATAGATGACAAGGTAGTATATCAAACATTGGTTCACAAAGATGGTGCA   838
DSM-29754         GTTCATTACTATATAGATGACAAGGTAGTATATCAAACATTGGTTCACAAAGATGGTGCA   838
DSM-29687         GTTCATTACTATATAGATGACAAGGTAGTATATCAAACATTGGTTCACAAAGATGGTGCA   838
DSM-29690         GTTCATTACTATATAGATGACAAGGTAGTATATCAAACATTGGTTCACAAAGATGGTGCA   838
DSM-29691         GTTCATTACTATATAGATGACAAGGTAGTATATCAAACATTGGTTCACAAAGATGGTGCA   838
DSM-29092         GTTCATTACTATATAGATGACAAGGTAGTATATCAAACATTGGTTCACAAAGATGGTGCA   838
DSM-29093         GTTCATTACTATATAGATGACAAGGTAGTATATCAAACATTGGTTCACAAAGATGGTGCA   838
DSM-29756         GTTCATTACTATATAGATGACAAGGTAGTATATCAAACATTGGTTCACAAAGATGGTGCA   838
DSM-29094         GTTCATTACTATATAGATGACAAGGTAGTATATCAAACATTGGTTCACAAAGATGGTGCA   838
2007855           GTTCATTACTATATGGATGACAAGGTAGTATATCAAACATTGGTTCACAAAGATGGTGCA   838
BI1               GTTCATTACTATATGGATGACAAGGTAGTATATCAAACATTGGTTCACAAAGATGGTGCA   838
DSM-28196         GTTCATTACTATATGGATGACAAGGTAGTATATCAAACATTGGTTCACAAAGATGGTGCA   838
DSM-27638         GTTCATTACTATATGGATGACAAGGTAGTATATCAAACATTGGTTCACAAAGATGGTGCA   838
DSM-27640         GTTCATTACTATATGGATGACAAGGTAGTATATCAAACATTGGTTCACAAAGATGGTGCA   838
DSM-29680         GTTCATTACTATATGGATGACAAGGTAGTATATCAAACATTGGTTCACAAAGATGGTGCA   838
R20291            GTTCATTACTATATGGATGACAAGGTAGTATATCAAACATTGGTTCACAAAGATGGTGCA   838
DSM-29747         GTTCATTACTATATGGATGACAAGGTAGTATATCAAACATTGGTTCACAAAGATGGTGCA   838
DSM-29752         GTTCATTACTATATGGATGACAAGGTAGTATATCAAACATTGGTTCACAAAGATGGTGCA   838
DSM-29753         GTTCATTACTATATGGATGACAAGGTAGTATATCAAACATTGGTTCACAAAGATGGTGCA   838
M120              GTTCATTACTATATGGATGACAAGGTAGTATATCAAACATTGGTTCACAAAGATGGTGCA   838
                  ************** *********************************************
```

```
M68               TGGTCAGTTGGTAAAATCTATTAAGCTACATTAGTTACAGATATCACAAACTATAATAGT   898
CF5               TGGTCAGTTGGTAAAATCTATTAAGCTACATTAGTTACAGATATCACAAACTATAATAGT   898
DSM-29095         TGGTCAGTTGGTAAAATCTATTAAGCTACATTAGTTACAGATATCACAAACTATAATAGT   898
DSM-29090         TGGTCAGTTGGTAAAATCTATTAAGCTACATTAGTTACAGATATCACAAACTATAATAGT   898
DSM-28194         TGGTCAGTTGGTAAAATCTATTAAGCTACATTAGTTACAGATATCACAAACTATAATAGT   898
DSM-28197         TGGTCAGTTGGTAAAATCTATTAAGCTACATTAGTTACAGATATCACAAACTATAATAGT   898
DSM-1296T         TGGTCAGTTGGTAAAATCTATTAAGCTACATTAGTTACAGATATCACAAACTATAATAGT   898
DSM-27639         TGGTCAGTTGGTAAAATCTATTAAGCTACATTAGTTACAGATATCACAAACTATAATAGT   898
DSM-29675         TGGTCAGTTGGTAAAATCTATTAAGCTACATTAGTTACAGATATCACAAACTATAATAGT   898
DSM-29676         TGGTCAGTTGGTAAAATCTATTAAGCTACATTAGTTACAGATATCACAAACTATAATAGT   898
DSM-29677         TGGTCAGTTGGTAAAATCTATTAAGCTACATTAGTTACAGATATCACAAACTATAATAGT   898
DSM-29678         TGGTCAGTTGGTAAAATCTATTAAGCTACATTAGTTACAGATATCACAAACTATAATAGT   898
DSM-29679         TGGTCAGTTGGTAAAATCTATTAAGCTACATTAGTTACAGATATCACAAACTATAATAGT   898
DSM-29683         TGGTCAGTTGGTAAAATCTATTAAGCTACATTAGTTACAGATATCACAAACTATAATAGT   898
DSM-29684         TGGTCAGTTGGTAAAATCTATTAAGCTACATTAGTTACAGATATCACAAACTATAATAGT   898
DSM-29685         TGGTCAGTTGGTAAAATCTATTAAGCTACATTAGTTACAGATATCACAAACTATAATAGT   898
DSM-29686         TGGTCAGTTGGTAAAATCTATTAAGCTACATTAGTTACAGATATCACAAACTATAATAGT   898
DSM-29089         TGGTCAGTTGGTAAAATCTATTAAGCTACATTAGTTACAGATATCACAAACTATAATAGT   898
DSM-29689         TGGTCAGTTGGTAAAATCTATTAAGCTACATTAGTTACAGATATCACAAACTATAATAGT   898
DSM-29692         TGGTCAGTTGGTAAAATCTATTAAGCTACATTAGTTACAGATATCACAAACTATAATAGT   898
DSM-29091         TGGTCAGTTGGTAAAATCTATTAAGCTACATTAGTTACAGATATCACAAACTATAATAGT   898
630               TGGTCAGTTGGTAAAATCTATTAAGCTACATTAGTTACAGATATCACAAACTATAATAGT   898
630-delta-erm     TGGTCAGTTGGTAAAATCTATTAAGCTACATTAGTTACAGATATCACAAACTATAATAGT   898
DSM-29744         TGGTCAGTTGGTAAAATCTATTAAGCTACATTAGTTACAGATATCACAAACTATAATAGT   898
DSM-29745         TGGTCAGTTGGTAAAATCTATTAAGCTACATTAGTTACAGATATCACAAACTATAATAGT   898
DSM-29754         TGGTCAGTTGGTAAAATCTATTAAGCTACATTAGTTACAGATATCACAAACTATAATAGT   898
DSM-29687         TGGTCAGTTGGTAAAATCTATTAAGCTACATTAGTTACAGATATCACAAACTATAATAGT   898
DSM-29690         TGGTCAGTTGGTAAAATCTATTAAGCTACATTAGTTACAGATATCACAAACTATAATAGT   898
DSM-29691         TGGTCAGTTGGTAAAATCTATTAAGCTACATTAGTTACAGATATCACAAACTATAATAGT   898
DSM-29092         TGGTCAGTTGGTAAAATCTATTAAGCTACATTAGTTACAGATATCACAAACTATAATAGT   898
DSM-29093         TGGTCAGTTGGTAAAATCTATTAAGCTACATTAGTTACAGATATCACAAACTATAATAGT   898
DSM-29756         TGGTCAGTTGGTAAAATCTATTAAGCTACATTAGTTACAGATATCACAAACTATAATAGT   898
DSM-29094         TGGTCAGTTGGTAAAATCTATTAAGCTACATTAGTTACAGATATCACAAACTATAATAGT   898
2007855           TGGTCAGTTGGTAAAATCTATTAAGCTACATTAGTTACAGATATCACAAACTATAATAGT   898
BI1               TGGTCAGTTGGTAAAATCTATTAAGCTACATTAGTTACAGATATCACAAACTATAATAGT   898
DSM-28196         TGGTCAGTTGGTAAAATCTATTAAGCTACATTAGTTACAGATATCACAAACTATAATAGT   898
DSM-27638         TGGTCAGTTGGTAAAATCTATTAAGCTACATTAGTTACAGATATCACAAACTATAATAGT   898
DSM-27640         TGGTCAGTTGGTAAAATCTATTAAGCTACATTAGTTACAGATATCACAAACTATAATAGT   898
DSM-29680         TGGTCAGTTGGTAAAATCTATTAAGCTACATTAGTTACAGATATCACAAACTATAATAGT   898
R20291            TGGTCAGTTGGTAAAATCTATTAAGCTACATTAGTTACAGATATCACAAACTATAATAGT   898
DSM-29747         TGGTCAGTTGGTAAAATCTATTAAGCTACATTAGTTACAGATATCACAAACTATAATAGT   898
DSM-29752         TGGTCAGTTGGTAAAATCTATTAAGCTACATTAGTTACAGATATCACAAACTATAATAGT   898
DSM-29753         TGGTCAGTTGGTAAAATCTATTAAGCTACATTAGTTACAGATATCACAAACTATAATAGT   898
M120              TGGTCAGTTGGTAAAATCTATTAAGCTACATTAGTTACAGATATCACAAACTATAATAGT   898
                  ************************************************************
```

tcdL translational stop

```
M68             TAAACATAGAAATATGTGTAAATTGTGATGGAAATTATTCAAAAACACAAAAATACGTGA   958
CF5             TAAACATAGAAATATGTGTAAATTGTGATGGAAATTATTCAAAAACACAAAAATACGTGA   958
DSM-29095       TAAACATAGAAATATGTGTAAATTGTGATGGAAATTATTCAAAAACACAAAAATACGTGA   958
DSM-29090       TAAACATAGAAATATGTGTAAATTGTGATGGAAATTATTCAAAAACACAAAAATACGTGA   958
DSM-28194       TAAACATAGAAATATGTGTAAATTGTGATGGAAATTATTCAAAAACACAAAAATACGTGA   958
DSM-28197       TAAACATAGAAATATGTGTAAATTGTGATGGAAATTATTCAAAAACACAAAAATACGTGA   958
DSM-1296T       TAAACATAGAAATATGTGTAAATTGTGATGGAAATTATTCAAAAACACAAAAATACGTGA   958
DSM-27639       TAAACATAGAAATATGTGTAAATTGTGATGGAAATTATTCAAAAACACAAAAATACGTGA   958
DSM-29675       TAAACATAGAAATATGTGTAAATTGTGATGGAAATTATTCAAAAACACAAAAATACGTGA   958
DSM-29676       TAAACATAGAAATATGTGTAAATTGTGATGGAAATTATTCAAAAACACAAAAATACGTGA   958
DSM-29677       TAAACATAGAAATATGTGTAAATTGTGATGGAAATTATTCAAAAACACAAAAATACGTGA   958
DSM-29678       TAAACATAGAAATATGTGTAAATTGTGATGGAAATTATTCAAAAACACAAAAATACGTGA   958
DSM-29679       TAAACATAGAAATATGTGTAAATTGTGATGGAAATTATTCAAAAACACAAAAATACGTGA   958
DSM-29683       TAAACATAGAAATATGTGTAAATTGTGATGGAAATTATTCAAAAACACAAAAATACGTGA   958
DSM-29684       TAAACATAGAAATATGTGTAAATTGTGATGGAAATTATTCAAAAACACAAAAATACGTGA   958
DSM-29685       TAAACATAGAAATATGTGTAAATTGTGATGGAAATTATTCAAAAACACAAAAATACGTGA   958
DSM-29686       TAAACATAGAAATATGTGTAAATTGTGATGGAAATTATTCAAAAACACAAAAATACGTGA   958
DSM-29089       TAAACATAGAAATATGTGTAAATTGTGATGGAAATTATTCAAAAACACAAAAATACGTGA   958
DSM-29689       TAAACATAGAAATATGTGTAAATTGTGATGGAAATTATTCAAAAACACAAAAATACGTGA   958
DSM-29692       TAAACATAGAAATATGTGTAAATTGTGATGGAAATTATTCAAAAACACAAAAATACGTGA   958
DSM-29091       TAAACATAGAAATATGTGTAAATTGTGATGGAAATTATTCAAAAACACAAAAATACGTGA   958
630             TAAACATAGAAATATGTGTAAATTGTGATGGAAATTATTCAAAAACACAAAAATACGTGA   958
630-delta-erm   TAAACATAGAAATATGTGTAAATTGTGATGGAAATTATTCAAAAACACAAAAATACGTGA   958
DSM-29744       TAAACATAGAAATATGTGTAAATTGTGATGGAAATTATTCAAAAACACAAAAATACGTGA   958
DSM-29745       TAAACATAGAAATATGTGTAAATTGTGATGGAAATTATTCAAAAACACAAAAATACGTGA   958
DSM-29754       TAAACATAGAAATATGTGTAAATTGTGATGGAAATTATTCAAAAACACAAAAATACGTGA   958
DSM-29687       TAAACATAGAAATATGTGTAAATTGTGATTGAAATTATTCAAAAACACAAAAATACGTGA   958
DSM-29690       TAAACATAGAAATATGTGTAAATTGTGATTGAAATTATTCAAAAACACAAAAATACGTGA   958
DSM-29691       TAAACATAGAAATATGTGTAAATTGTGATTGAAATTATTCAAAAACACAAAAATACGTGA   958
DSM-29092       TAAACATAGAAATATGTGTAAATTGTGATTGAAATTATTCAAAAACACAAAAATACGTGA   958
DSM-29093       TAAACATAGAAATATGTGTAAATTGTGATTGAAATTATTCAAAAACACAAAAATACGTGA   958
DSM-29756       TAAACATAGAAATATGTGTAAATTGTGATTGAAATTATTCAAAAACACAAAAATACGTGA   958
DSM-29094       TAAACATAGAAATATGTGTAAATTGTGATTGAAATTATTCAAAAACACAAAAATACGTGA   958
2007855         TAAACATAGAAATATGTGTAAATTGTGATGGAAATTATTCAAAAACACAAAAATACGTGA   958
BI1             TAAACATAGAAATATGTGTAAATTGTGATGGAAATTATTCAAAAACACAAAAATACGTGA   958
DSM-28196       TAAACATAGAAATATGTGTAAATTGTGATGGAAATTATTCAAAAACACAAAAATACGTGA   958
DSM-27638       TAAACATAGAAATATGTGTAAATTGTGATGGAAATTATTCAAAAACACAAAAATACGTGA   958
DSM-27640       TAAACATAGAAATATGTGTAAATTGTGATGGAAATTATTCAAAAACACAAAAATACGTGA   958
DSM-29680       TAAACATAGAAATATGTGTAAATTGTGATGGAAATTATTCAAAAACACAAAAATACGTGA   958
R20291          TAAACATAGAAATATGTGTAAATTGTGATGGAAATTATTCAAAAACACAAAAATACGTGA   958
DSM-29747       TAAACATAGAAATATGTGTAAATTGTGATGGAAATTATTCAAAAACACAAAAATACGTGA   958
DSM-29752       TAAACATAGAAATATGTGTAAATTGTGATGGAAATTATTCAAAAACACAAAAATACGTGA   958
DSM-29753       TAAACATAGAAATATGTGTAAATTGTGATGGAAATTATTCAAAAACACAAAAATACGTGA   958
M120            TAAACATAGAAATATGTGTAAATTGTGATGGAAATTATTCAAAAACACAAAAATACGTGA   958
                ***************************** ******************************
```

```
M68                 TGAAGGACAAAATGATAGAGAAAATAAGTATCAAACCTTAATAAATGATTTAATTGATAG        1018
CF5                 TGAAGGACAAAATGATAGAGAAAATAAGTATCAAACCTTAATAATTGATTTAATTGATAG        1018
DSM-29095           TGAAGGACAAAATGATATAGAAAATAAGTATCAAACCTTAATAAATGATTTAATTGATAG        1018
DSM-29090           TGAAGGACAAAATGATATAGAAAATAAGTATCAAACCTTAATAAATGATTTAATTGATAG        1018
DSM-28194           TGAAGGACAAAATGATATAGAAAATAAGTATCAAACCTTAATAAATGATTTAATTGATAG        1018
DSM-28197           TGAAGGACAAAATGATATAGAAAATAAGTATCAAACCTTAATAAATGATTTAATTGATAG        1018
DSM-1296T           TGAAGGACAAAATGATATAGAAAATAAGTATCAAACCTTAATAAATGATTTAATTGATAG        1018
DSM-27639           TGAAGGACAAAATGATATAGAAAATAAGTATCAAACCTTAATAAATGATTTAATTGATAG        1018
DSM-29675           TGAAGGACAAAATGATATAGAAAATAAGTATCAAACCTTAATAAATGATTTAATTGATAG        1018
DSM-29676           TGAAGGACAAAATGATATAGAAAATAAGTATCAAACCTTAATAAATGATTTAATTGATAG        1018
DSM-29677           TGAAGGACAAAATGATATAGAAAATAAGTATCAAACCTTAATAAATGATTTAATTGATAG        1018
DSM-29678           TGAAGGACAAAATGATATAGAAAATAAGTATCAAACCTTAATAAATGATTTAATTGATAG        1018
DSM-29679           TGAAGGACAAAATGATATAGAAAATAAGTATCAAACCTTAATAAATGATTTAATTGATAG        1018
DSM-29683           TGAAGGACAAAATGATATAGAAAATAAGTATCAAACCTTAATAAATGATTTAATTGATAG        1018
DSM-29684           TGAAGGACAAAATGATATAGAAAATAAGTATCAAACCTTAATAAATGATTTAATTGATAG        1018
DSM-29685           TGAAGGACAAAATGATATAGAAAATAAGTATCAAACCTTAATAAATGATTTAATTGATAG        1018
DSM-29686           TGAAGGACAAAATGATATAGAAAATAAGTATCAAACCTTAATAAATGATTTAATTGATAG        1018
DSM-29089           TGAAGGACAAAATGATATAGAAAATAAGTATCAAACCTTAATAAATGATTTAATTGATAG        1018
DSM-29689           TGAAGGACAAAATGATATAGAAAATAAGTATCAAACCTTAATAAATGATTTAATTGATAG        1018
DSM-29692           TGAAGGACAAAATGATATAGAAAATAAGTATCAAACCTTAATAAATGATTTAATTGATAG        1018
DSM-29091           TGAAGGACAAAATGATATAGAAAATAAGTATCAAACCTTAATAAATGATTTAATTGATAG        1018
630                 TGAAGGACAAAATGATATAGAAAATAAGTATCAAACCTTAATAAATGATTTAATTGATAG        1018
630-delta-erm       TGAAGGACAAAATGATATAGAAAATAAGTATCAAACCTTAATAAATGATTTAATTGATAG        1018
DSM-29744           TGAAGGACAAAATGATATAGAAAATAAGTATCAAACCTTAATAAATGATTTAATTGATAG        1018
DSM-29745           TGAAGGACAAAATGATATAGAAAATAAGTATCAAACCTTAATAAATGATTTAATTGATAG        1018
DSM-29754           TGAAGGACAAAATGATATAGAAAATAAGTATCAAACCTTAATAAATGATTTAATTGATAG        1018
DSM-29687           TGAAGGACAAAATGATATAGAAAATAAGTATCAAACCTTAATAAATGATTTAATTGATAG        1018
DSM-29690           TGAAGGACAAAATGATATAGAAAATAAGTATCAAACCTTAATAAATGATTTAATTGATAG        1018
DSM-29691           TGAAGGACAAAATGATATAGAAAATAAGTATCAAACCTTAATAAATGATTTAATTGATAG        1018
DSM-29092           TGAAGGACAAAATGATATAGAAAATAAGTATCAAACCTTAATAAATGATTTAATTGATAG        1018
DSM-29093           TGAAGGACAAAATGATATAGAAAATAAGTATCAAACCTTAATAAATGATTTAATTGATAG        1018
DSM-29756           TGAAGGACAAAATGATATAGAAAATAAGTATCAAACCTTAATAAATGATTTAATTGATAG        1018
DSM-29094           TGAAGGACAAAATGATATAGAAAATAAGTATCAAACCTTAATAAATGATTTAATTGATAG        1018
2007855             TGAAGGACAAAATGATAGAGAAAATAAGTATCAAACCTTAATAAATGATTTAATTGATAG        1018
BI1                 TGAAGGACAAAATGATAGAGAAAATAAGTATCAAACCTTAATAAATGATTTAATTGATAG        1018
DSM-28196           TGAAGGACAAAATGATAGAGAAAATAAGTATCAAACCTTAATAAATGATTTAATTGATAG        1018
DSM-27638           TGAAGGACAAAATGATAGAGAAAATAAGTATCAAACCTTAATAAATGATTTAATTGATAG        1018
DSM-27640           TGAAGGACAAAATGATAGAGAAAATAAGTATCAAACCTTAATAAATGATTTAATTGATAG        1018
DSM-29680           TGAAGGACAAAATGATAGAGAAAATAAGTATCAAACCTTAATAAATGATTTAATTGATAG        1018
R20291              TGAAGGACAAAATGATAGAGAAAATAAGTATCAAACCTTAATAAATGATTTAATTGATAG        1018
DSM-29747           TGAAGGACAAAATGATAGAGAAAATAAGTATCAAACCTTAATAAATGATTTAATTGATAG        1018
DSM-29752           TGAAGGACAAAATGATAGAGAAAATAAGTATCAAACCTTAATAAATGATTTAATTGATAG        1018
DSM-29753           TGAAGGACAAAATGATAGAGAAAATAAGTATCAAACCTTAATAAATGATTTAATTGATAG        1018
M120                TGAAGGACAAAATGATAGAGAAAATAAGTATCAAACCTTAATAAATGATTTAATTGATAG        1018
                    ***************** ************************** ***************
```

```
M68              TTTAAAAGTTATAGGAAAAATATATATAAAGAAATAAAAACATTAAAAAAAATATAAGATATG       1078
CF5              TTTAAAAGTTATAGGAAAAATATATATAAAGAAATAAAAACATTAAAAAAAATATAAGATATG       1078
DSM-29095        TTTAAAAGTTATAGGAAAAATATATATAAAGAAATAAAAACATTAAAAAAAATATAAGATATG       1078
DSM-29090        TTTAAAAGTTATAGGAAAAATATATATAAAGAAATAAAAACATTAAAAAAAATATAAGATATG       1078
DSM-28194        TTTAAAAGTTATAGGAAAAATATATATAAAGAAATAAAAACATTAAAAAAAATATAAGATATG       1078
DSM-28197        TTTAAAAGTTATAGGAAAAATATATATAAAGAAATAAAAACATTAAAAAAAATATAAGATATG       1078
DSM-1296T        TTTAAAAGTTATAGGAAAAATATATATAAAGAAATAAAAACATTAAAAAAAATATAAGATATG       1078
DSM-27639        TTTAAAAGTTATAGGAAAAATATATATAAAGAAATAAAAACATTAAAAAAAATATAAGATATG       1078
DSM-29675        TTTAAAAGTTATAGGAAAAATATATATAAAGAAATAAAAACATTAAAAAAAATATAAGATATG       1078
DSM-29676        TTTAAAAGTTATAGGAAAAATATATATAAAGAAATAAAAACATTAAAAAAAATATAAGATATG       1078
DSM-29677        TTTAAAAGTTATAGGAAAAATATATATAAAGAAATAAAAACATTAAAAAAAATATAAGATATG       1078
DSM-29678        TTTAAAAGTTATAGGAAAAATATATATAAAGAAATAAAAACATTAAAAAAAATATAAGATATG       1078
DSM-29679        TTTAAAAGTTATAGGAAAAATATATATAAAGAAATAAAAACATTAAAAAAAATATAAGATATG       1078
DSM-29683        TTTAAAAGTTATAGGAAAAATATATATAAAGAAATAAAAACATTAAAAAAAATATAAGATATG       1078
DSM-29684        TTTAAAAGTTATAGGAAAAATATATATAAAGAAATAAAAACATTAAAAAAAATATAAGATATG       1078
DSM-29685        TTTAAAAGTTATAGGAAAAATATATATAAAGAAATAAAAACATTAAAAAAAATATAAGATATG       1078
DSM-29686        TTTAAAAGTTATAGGAAAAATATATATAAAGAAATAAAAACATTAAAAAAAATATAAGATATG       1078
DSM-29089        TTTAAAAGTTATAGGAAAAATATATATAAAGAAATAAAAACATTAAAAAAAATATAAGATATG       1078
DSM-29689        TTTAAAAGTTATAGGAAAAATATATATAAAGAAATAAAAACATTAAAAAAAATATAAGATATG       1078
DSM-29692        TTTAAAAGTTATAGGAAAAATATATATAAAGAAATAAAAACATTAAAAAAAATATAAGATATG       1078
DSM-29091        TTTAAAAGTTATAGGAAAAATATATATAAAGAAATAAAAACATTAAAAAAAATATAAGATATG       1078
630              TTTAAAAGTTATAGGAAAAATATATATAAAGAAATAAAAACATTAAAAAAAATATAAGATATG       1078
630-delta-erm    TTTAAAAGTTATAGGAAAAATATATATAAAGAAATAAAAACATTAAAAAAAATATAAGATATG       1078
DSM-29744        TTTAAAAGTTATAGGAAAAATATATATAAAGAAATAAAAACATTAAAAAAAATATAAGATATG       1078
DSM-29745        TTTAAAAGTTATAGGAAAAATATATATAAAGAAATAAAAACATTAAAAAAAATATAAGATATG       1078
DSM-29754        TTTAAAAGTTATAGGAAAAATATATATAAAGAAATAAAAACATTAAAAAAAATATAAGATATG       1078
DSM-29687        TTTAAAAGTTATAGGAAAAATATATATAAAGAAATAAAAACATTAAAAAAAATATAAGATATG       1078
DSM-29690        TTTAAAAGTTATAGGAAAAATATATATAAAGAAATAAAAACATTAAAAAAAATATAAGATATG       1078
DSM-29691        TTTAAAAGTTATAGGAAAAATATATATAAAGAAATAAAAACATTAAAAAAAATATAAGATATG       1078
DSM-29092        TTTAAAAGTTATAGGAAAAATATATATAAAGAAATAAAAACATTAAAAAAAATATAAGATATG       1078
DSM-29093        TTTAAAAGTTATAGGAAAAATATATATAAAGAAATAAAAACATTAAAAAAAATATAAGATATG       1078
DSM-29756        TTTAAAAGTTATAGGAAAAATATATATAAAGAAATAAAAACATTAAAAAAAATATAAGATATG       1078
DSM-29094        TTTAAAAGTTATAGGAAAAATATATATAAAGAAATAAAAACATTAAAAAAAATATAAGATATG       1078
2007855          TTTAAAAGTTATAGGAAAAATATATATAAAGAAATAAAAACATTAAAAAAAATATAAGATATG       1078
BI1              TTTAAAAGTTATAGGAAAAATATATATAAAGAAATAAAAACATTAAAAAAAATATAAGATATG       1078
DSM-28196        TTTAAAAGTTATAGGAAAAATATATATAAAGAAATAAAAACATTAAAAAAAATATAAGATATG       1078
DSM-27638        TTTAAAAGTTATAGGAAAAATATATATAAAGAAATAAAAACATTAAAAAAAATATAAGATATG       1078
DSM-27640        TTTAAAAGTTATAGGAAAAATATATATAAAGAAATAAAAACATTAAAAAAAATATAAGATATG       1078
DSM-29680        TTTAAAAGTTATAGGAAAAATATATATAAAGAAATAAAAACATTAAAAAAAATATAAGATATG       1078
R20291           TTTAAAAGTTATAGGAAAAATATATATAAAGAAATAAAAACATTAAAAAAAATATAAGATATG       1078
DSM-29747        TTTAAAAGTTATAGGAAAAATATATATAAAGAAATAAAAACATTAAAAAAAATATAAGATATG       1078
DSM-29752        TTTAAAAGTTATAGGAAAAATATATATAAAGAAATAAAAACATTAAAAAAAATATAAGATATG       1078
DSM-29753        TTTAAAAGTTATAGGAAAAATATATATAAAGAAATAAAAACATTAAAAAAAATATAAGATATG       1078
M120             TTTAAAAGTTATAGGAAAAATATATATAAAGAAATAAAAACATTAAAAAAAATATAAGATATG       1078
                 **************************************************************
```

```
M68                 ---------TTCTATCATACAATCTCCTTATCTAATAGAAGAGTCAATTAACTAATTGAG       1129
CF5                 ---------TTCTATCATACAATCTCCTTATCTAATAGAAGAGTCAATTAACTAATTGAG       1129
DSM-29095           TTTACAAATTACTATCAGACAATCTCCTTATCTAATAGAAGAGTCAATTAACTAATTGAG       1138
DSM-29090           TTTACAAATTACTATCAGACAATCTCCTTATCTAATAGAAGAGTCAATTAACTAATTGAG       1138
DSM-28194           TTTACAAATTACTATCAGACAATCTCCTTATCTAATAGAAGAGTCAATTAACTAATTGAG       1138
DSM-28197           TTTACAAATTACTATCAGACAATCTCCTTATCTAATAGAAGAGTCAATTAACTAATTGAG       1138
DSM-1296T           TTTACAAATTACTATCAGACAATCTCCTTATCTAATAGAAGAGTCAATTAACTAATTGAG       1138
DSM-27639           TTTACAAATTACTATCAGACAATCTCCTTATCTAATAGAAGAGTCAATTAACTAATTGAG       1138
DSM-29675           TTTACAAATTACTATCAGACAATCTCCTTATCTAATAGAAGAGTCAATTAACTAATTGAG       1138
DSM-29676           TTTACAAATTACTATCAGACAATCTCCTTATCTAATAGAAGAGTCAATTAACTAATTGAG       1138
DSM-29677           TTTACAAATTACTATCAGACAATCTCCTTATCTAATAGAAGAGTCAATTAACTAATTGAG       1138
DSM-29678           TTTACAAATTACTATCAGACAATCTCCTTATCTAATAGAAGAGTCAATTAACTAATTGAG       1138
DSM-29679           TTTACAAATTACTATCAGACAATCTCCTTATCTAATAGAAGAGTCAATTAACTAATTGAG       1138
DSM-29683           TTTACAAATTACTATCAGACAATCTCCTTATCTAATAGAAGAGTCAATTAACTAATTGAG       1138
DSM-29684           TTTACAAATTACTATCAGACAATCTCCTTATCTAATAGAAGAGTCAATTAACTAATTGAG       1138
DSM-29685           TTTACAAATTACTATCAGACAATCTCCTTATCTAATAGAAGAGTCAATTAACTAATTGAG       1138
DSM-29686           TTTACAAATTACTATCAGACAATCTCCTTATCTAATAGAAGAGTCAATTAACTAATTGAG       1138
DSM-29089           TTTACAAATTACTATCAGACAATCTCCTTATCTAATAGAAGAGTCAATTAACTAATTGAG       1138
DSM-29689           TTTACAAATTACTATCAGACAATCTCCTTATCTAATAGAAGAGTCAATTAACTAATTGAG       1138
DSM-29692           TTTACAAATTACTATCAGACAATCTCCTTATCTAATAGAAGAGTCAATTAACTAATTGAG       1138
DSM-29091           TTTACAAATTACTATCAGACAATCTCCTTATCTAATAGAAGAGTCAATTAACTAATTGAG       1138
630                 TTTACAAATTACTATCAGACAATCTCCTTATCTAATAGAAGAGTCAATTAACTAATTGAG       1138
630-delta-erm       TTTACAAATTACTATCAGACAATCTCCTTATCTAATAGAAGAGTCAATTAACTAATTGAG       1138
DSM-29744           TTTACAAATTACTATCAGACAATCTCCTTATCTAATAGAAGAGTCAATTAACTAATTGAG       1138
DSM-29745           TTTACAAATTACTATCAGACAATCTCCTTATCTAATAGAAGAGTCAATTAACTAATTGAG       1138
DSM-29754           TTTACAAATTACTATCAGACAATCTCCTTATCTAATAGAAGAGTCAATTAACTAATTGAG       1138
DSM-29687           TTTACAAATTACTATCAGACAATCTCCTTATCTAATAGAAGAGTCAATTAACTAATTGAG       1138
DSM-29690           TTTACAAATTACTATCAGACAATCTCCTTATCTAATAGAAGAGTCAATTAACTAATTGAG       1138
DSM-29691           TTTACAAATTACTATCAGACAATCTCCTTATCTAATAGAAGAGTCAATTAACTAATTGAG       1138
DSM-29092           TTTACAAATTACTATCAGACAATCTCCTTATCTAATAGAAGAGTCAATTAACTAATTGAG       1138
DSM-29093           TTTACAAATTACTATCAGACAATCTCCTTATCTAATAGAAGAGTCAATTAACTAATTGAG       1138
DSM-29756           TTTACAAATTACTATCAGACAATCTCCTTATCTAATAGAAGAGTCAATTAACTAATTGAG       1138
DSM-29094           TTTACAAATTACTATCAGACAATCTCCTTATCTAATAGAAGAGTCAATTAACTAATTGAG       1138
2007855             TTTACAAATTACTATCAGACAATCTCCTTATCTAATAGAAGAGTCAATTAACTAATTGAG       1138
BI1                 TTTACAAATTACTATCAGACAATCTCCTTATCTAATAGAAGAGTCAATTAACTAATTGAG       1138
DSM-28196           TTTACAAATTACTATCAGACAATCTCCTTATCTAATAGAAGAGTCAATTAACTAATTGAG       1138
DSM-27638           TTTACAAATTACTATCAGACAATCTCCTTATCTAATAGAAGAGTCAATTAACTAATTGAG       1138
DSM-27640           TTTACAAATTACTATCAGACAATCTCCTTATCTAATAGAAGAGTCAATTAACTAATTGAG       1138
DSM-29680           TTTACAAATTACTATCAGACAATCTCCTTATCTAATAGAAGAGTCAATTAACTAATTGAG       1138
R20291              TTTACAAATTACTATCAGACAATCTCCTTATCTAATAGAAGAGTCAATTAACTAATTGAG       1138
DSM-29747           TTTACAAATTACTATCAGACAATCTCCTTATCTAATAGAAGAGTCAATTAACTAATTGAG       1138
DSM-29752           TTTACAAATTACTATCAGACAATCTCCTTATCTAATAGAAGAGTCAATTAACTAATTGAG       1138
DSM-29753           TTTACAAATTACTATCAGACAATCTCCTTATCTAATAGAAGAGTCAATTAACTAATTGAG       1138
M120                TTTACAAATTACTATCAGACAATCTCCTTATCTAATAGAAGAGTCAATTAACTAATTGAG       1138
                             * ****** ******************************************
```

```
M68               TATCTTTAAATTGAAATGTTAGGAAGTGATTTAAATATGAAAACTTAAATTATAAAAAAT     1189
CF5               TATCTTTAAATTGAAATGTTAGGAAGTGATTTAAATATGAAAACTTAAATTATAAAAAAT     1189
DSM-29095         TATCTTTAAATTGAAATGTTAGGAAGTGATTTAAATATGAAAACTTAAATTATAAAAAAT     1198
DSM-29090         TATCTTTAAATTGAAATGTTAGGAAGTGATTTAAATATGAAAACTTAAATTATAAAAAAT     1198
DSM-28194         TATCTTTAAATTGAAATGTTAGGAAGTGATTTAAATATGAAAACTTAAATTATAAAAAAT     1198
DSM-28197         TATCTTTAAATTGAAATGTTAGGAAGTGATTTAAATATGAAAACTTAAATTATAAAAAAT     1198
DSM-1296T         TATCTTTAAATTGAAATGTTAGGAAGTGATTTAAATATGAAAACTTAAATTATAAAAAAT     1198
DSM-27639         TATCTTTAAATTGAAATGTTAGGAAGTGATTTAAATATGAAAACTTAAATTATAAAAAAT     1198
DSM-29675         TATCTTTAAATTGAAATGTTAGGAAGTGATTTAAATATGAAAACTTAAATTATAAAAAAT     1198
DSM-29676         TATCTTTAAATTGAAATGTTAGGAAGTGATTTAAATATGAAAACTTAAATTATAAAAAAT     1198
DSM-29677         TATCTTTAAATTGAAATGTTAGGAAGTGATTTAAATATGAAAACTTAAATTATAAAAAAT     1198
DSM-29678         TATCTTTAAATTGAAATGTTAGGAAGTGATTTAAATATGAAAACTTAAATTATAAAAAAT     1198
DSM-29679         TATCTTTAAATTGAAATGTTAGGAAGTGATTTAAATATGAAAACTTAAATTATAAAAAAT     1198
DSM-29683         TATCTTTAAATTGAAATGTTAGGAAGTGATTTAAATATGAAAACTTAAATTATAAAAAAT     1198
DSM-29684         TATCTTTAAATTGAAATGTTAGGAAGTGATTTAAATATGAAAACTTAAATTATAAAAAAT     1198
DSM-29685         TATCTTTAAATTGAAATGTTAGGAAGTGATTTAAATATGAAAACTTAAATTATAAAAAAT     1198
DSM-29686         TATCTTTAAATTGAAATGTTAGGAAGTGATTTAAATATGAAAACTTAAATTATAAAAAAT     1198
DSM-29089         TATCTTTAAATTGAAATGTTAGGAAGTGATTTAAATATGAAAACTTAAATTATAAAAAAT     1198
DSM-29689         TATCTTTAAATTGAAATGTTAGGAAGTGATTTAAATATGAAAACTTAAATTATAAAAAAT     1198
DSM-29692         TATCTTTAAATTGAAATGTTAGGAAGTGATTTAAATATGAAAACTTAAATTATAAAAAAT     1198
DSM-29091         TATCTTTAAATTGAAATGTTAGGAAGTGATTTAAATATGAAAACTTAAATTATAAAAAAT     1198
630               TATCTTTAAATTGAAATGTTAGGAAGTGATTTAAATATGAAAACTTAAATTATAAAAAAT     1198
630-delta-erm     TATCTTTAAATTGAAATGTTAGGAAGTGATTTAAATATGAAAACTTAAATTATAAAAAAT     1198
DSM-29744         TATCTTTAAATTGAAATGTTAGGAAGTGATTTAAATATGAAAACTTAAATTATAAAAAAT     1198
DSM-29745         TATCTTTAAATTGAAATGTTAGGAAGTGATTTAAATATGAAAACTTAAATTATAAAAAAT     1198
DSM-29754         TATCTTTAAATTGAAATGTTAGGAAGTGATTTAAATATGAAAACTTAAATTATAAAAAAT     1198
DSM-29687         TATCTTTAAATTGAAATGTTAGGAAGTGATTTAAATATGAAAACTTAAATTATAAAAAAT     1198
DSM-29690         TATCTTTAAATTGAAATGTTAGGAAGTGATTTAAATATGAAAACTTAAATTATAAAAAAT     1198
DSM-29691         TATCTTTAAATTGAAATGTTAGGAAGTGATTTAAATATGAAAACTTAAATTATAAAAAAT     1198
DSM-29092         TATCTTTAAATTGAAATGTTAGGAAGTGATTTAAATATGAAAACTTAAATTATAAAAAAT     1198
DSM-29093         TATCTTTAAATTGAAATGTTAGGAAGTGATTTAAATATGAAAACTTAAATTATAAAAAAT     1198
DSM-29756         TATCTTTAAATTGAAATGTTAGGAAGTGATTTAAATATGAAAACTTAAATTATAAAAAAT     1198
DSM-29094         TATCTTTAAATTGAAATGTTAGGAAGTGATTTAAATATGAAAACTTAAATTATAAAAAAT     1198
2007855           TATCTTTAAATTGAAATGTTAGGAAGTGATTTAAATATGAAAACTTAAATTATAAAAAAT     1198
BI1               TATCTTTAAATTGAAATGTTAGGAAGTGATTTAAATATGAAAACTTAAATTATAAAAAAT     1198
DSM-28196         TATCTTTAAATTGAAATGTTAGGAAGTGATTTAAATATGAAAACTTAAATTATAAAAAAT     1198
DSM-27638         TATCTTTAAATTGAAATGTTAGGAAGTGATTTAAATATGAAAACTTAAATTATAAAAAAT     1198
DSM-27640         TATCTTTAAATTGAAATGTTAGGAAGTGATTTAAATATGAAAACTTAAATTATAAAAAAT     1198
DSM-29680         TATCTTTAAATTGAAATGTTAGGAAGTGATTTAAATATGAAAACTTAAATTATAAAAAAT     1198
R20291            TATCTTTAAATTGAAATGTTAGGAAGTGATTTAAATATGAAAACTTAAATTATAAAAAAT     1198
DSM-29747         TATCTTTAAATTGAAATGTTAGGAAGTGATTTAAATATGAAAACTTAAATTATAAAAAAT     1198
DSM-29752         TATCTTTAAATTGAAATGTTAGGAAGTGATTTAAATATGAAAACTTAAATTATAAAAAAT     1198
DSM-29753         TATCTTTAAATTGAAATGTTAGGAAGTGATTTAAATATGAAAACTTAAATTATAAAAAAT     1198
M120              TATCTTTAAATTGAAATGTTAGGAAGTGATTTAAATATGAAAACTTAAATTATAAAAAAT     1198
                  ************************************************************
```

```
M68                 CAATATTAATTTATTTTAAAAAAATAGAAAGGAGTGTATAAGATTTATTTTCAAAGTTTA       1249
CF5                 CAATATTAATTTATTTTAAAAAAATAGAAAGGAGTGTATAAGATTTATTTTCAAAGTTTA       1249
DSM-29095           CAATATTAATTTATTTTTAAAAAATAGAAAGGAGTGTATAAGATTTATTTTCAAAGTTTA       1258
DSM-29090           CAATATTAATTTATTTTTAAAAAATAGAAAGGAGTGTATAAGATTTATTTTCAAAGTTTA       1258
DSM-28194           CAATATTAATTTATTTTTAAAAAATAGAAAGGAGTGTATAAGATTTATTTTCAAAGTTTA       1258
DSM-28197           CAATATTAATTTATTTTTAAAAAATAGAAAGGAGTGTATAAGATTTATTTTCAAAGTTTA       1258
DSM-1296T           CAATATTAATTTATTTTTAAAAAATAGAAAGGAGTGTATAAGATTTATTTTCAAAGTTTA       1258
DSM-27639           CAATATTAATTTATTTTTAAAAAATAGAAAGGAGTGTATAAGATTTATTTTCAAAGTTTA       1258
DSM-29675           CAATATTAATTTATTTTTAAAAAATAGAAAGGAGTGTATAAGATTTATTTTCAAAGTTTA       1258
DSM-29676           CAATATTAATTTATTTTTAAAAAATAGAAAGGAGTGTATAAGATTTATTTTCAAAGTTTA       1258
DSM-29677           CAATATTAATTTATTTTTAAAAAATAGAAAGGAGTGTATAAGATTTATTTTCAAAGTTTA       1258
DSM-29678           CAATATTAATTTATTTTTAAAAAATAGAAAGGAGTGTATAAGATTTATTTTCAAAGTTTA       1258
DSM-29679           CAATATTAATTTATTTTTAAAAAATAGAAAGGAGTGTATAAGATTTATTTTCAAAGTTTA       1258
DSM-29683           CAATATTAATTTATTTTTAAAAAATAGAAAGGAGTGTATAAGATTTATTTTCAAAGTTTA       1258
DSM-29684           CAATATTAATTTATTTTTAAAAAATAGAAAGGAGTGTATAAGATTTATTTTCAAAGTTTA       1258
DSM-29685           CAATATTAATTTATTTTTAAAAAATAGAAAGGAGTGTATAAGATTTATTTTCAAAGTTTA       1258
DSM-29686           CAATATTAATTTATTTTTAAAAAATAGAAAGGAGTGTATAAGATTTATTTTCAAAGTTTA       1258
DSM-29089           CAATATTAATTTATTTTTAAAAAATAGAAAGGAGTGTATAAGATTTATTTTCAAAGTTTA       1258
DSM-29689           CAATATTAATTTATTTTTAAAAAATAGAAAGGAGTGTATAAGATTTATTTTCAAAGTTTA       1258
DSM-29692           CAATATTAATTTATTTTTAAAAAATAGAAAGGAGTGTATAAGATTTATTTTCAAAGTTTA       1258
DSM-29091           CAATATTAATTTATTTTTAAAAAATAGAAAGGAGTGTATAAGATTTATTTTCAAAGTTTA       1258
630                 CAATATTAATTTATTTTTAAAAAATAGAAAGGAGTGTATAAGATTTATTTTCAAAGTTTA       1258
630-delta-erm       CAATATTAATTTATTTTTAAAAAATAGAAAGGAGTGTATAAGATTTATTTTCAAAGTTTA       1258
DSM-29744           CAATATTAATTTATTTTTAAAAAATAGAAAGGAGTGTATAAGATTTATTTTCAAAGTTTA       1258
DSM-29745           CAATATTAATTTATTTTTAAAAAATAGAAAGGAGTGTATAAGATTTATTTTCAAAGTTTA       1258
DSM-29754           CAATATTAATTTATTTTTAAAAAATAGAAAGGAGTGTATAAGATTTATTTTCAAAGTTTA       1258
DSM-29687           CAATATTAATTTATTTTTAAAAAATAGAAAGGAGTGTATAAGATTTATTTTCAAAGTTTA       1258
DSM-29690           CAATATTAATTTATTTTTAAAAAATAGAAAGGAGTGTATAAGATTTATTTTCAAAGTTTA       1258
DSM-29691           CAATATTAATTTATTTTTAAAAAATAGAAAGGAGTGTATAAGATTTATTTTCAAAGTTTA       1258
DSM-29092           CAATATTAATTTATTTTTAAAAAATAGAAAGGAGTGTATAAGATTTATTTTCAAAGTTTA       1258
DSM-29093           CAATATTAATTTATTTTTAAAAAATAGAAAGGAGTGTATAAGATTTATTTTCAAAGTTTA       1258
DSM-29756           CAATATTAATTTATTTTTAAAAAATAGAAAGGAGTGTATAAGATTTATTTTCAAAGTTTA       1258
DSM-29094           CAATATTAATTTATTTTTAAAAAATAGAAAGGAGTGTATAAGATTTATTTTCAAAGTTTA       1258
2007855             CAATATTAATTTATCTTTAAAAAATAGAAAGGAGTGTATAAGATTTATTTTCAAAGTTTA       1258
BI1                 CAATATTAATTTATCTTTAAAAAATAGAAAGGAGTGTATAAGATTTATTTTCAAAGTTTA       1258
DSM-28196           CAATATTAATTTATCTTTAAAAAATAGAAAGGAGTGTATAAGATTTATTTTCAAAGTTTA       1258
DSM-27638           CAATATTAATTTATCTTTAAAAAATAGAAAGGAGTGTATAAGATTTATTTTCAAAGTTTA       1258
DSM-27640           CAATATTAATTTATCTTTAAAAAATAGAAAGGAGTGTATAAGATTTATTTTCAAAGTTTA       1258
DSM-29680           CAATATTAATTTATCTTTAAAAAATAGAAAGGAGTGTATAAGATTTATTTTCAAAGTTTA       1258
R20291              CAATATTAATTTATCTTTAAAAAATAGAAAGGAGTGTATAAGATTTATTTTCAAAGTTTA       1258
DSM-29747           CAATATTAATTTATTTTTAAAAAATAGAAAGGAGTGTATAAGATTTATTTTCAAAGTTTA       1258
DSM-29752           CAATATTAATTTATTTTTAAAAAATAGAAAGGAGTGTATAAGATTTATTTTCAAAGTTTA       1258
DSM-29753           CAATATTAATTTATTTTTAAAAAATAGAAAGGAGTGTATAAGATTTATTTTCAAAGTTTA       1258
M120                CAATATTAATTTATTTTTAAAAAATAGAAAGGAGTGTATAAGATTTATTTTCAAAGTTTA       1258
                    ************** ** ******************************************
```

```
M68              AAAACAAGAAAATCAATTTAAATTTCAGAAGGAATAAATGTGGTTATAGAAGTGGATTTA      1309
CF5              AAAACAAGAAAATCAATTTAAATTTCAGAAGGAATAAATGTGGTTATAGAAGTGGATTTA      1309
DSM-29095        AAAACAAGAAAATCAATTTAAATTTCAGAAGGAATAAATGTGGTTATAGAAGTGGATTTA      1318
DSM-29090        AAAACAAGAAAATCAATTTAAATTTCAGAAGGAATAAATGTGGTTATAGAAGTGGATTTA      1318
DSM-28194        AAAACAAGAAAATCAATTTAAATTTCAGAAGGAATAAATGTGGTTATAGAAGTGGATTTA      1318
DSM-28197        AAAACAAGAAAATCAATTTAAATTTCAGAAGGAATAAATGTGGTTATAGAAGTGGATTTA      1318
DSM-1296T        AAAACAAGAAAATCAATTTAAATTTCAGAAGGAATAAATGTGGTTATAGAAGTGGATTTA      1318
DSM-27639        AAAACAAGAAAATCAATTTAAATTTCAGAAGGAATAAATGTGGTTATAGAAGTGGATTTA      1318
DSM-29675        AAAACAAGAAAATCAATTTAAATTTCAGAAGGAATAAATGTGGTTATAGAAGTGGATTTA      1318
DSM-29676        AAAACAAGAAAATCAATTTAAATTTCAGAAGGAATAAATGTGGTTATAGAAGTGGATTTA      1318
DSM-29677        AAAACAAGAAAATCAATTTAAATTTCAGAAGGAATAAATGTGGTTATAGAAGTGGATTTA      1318
DSM-29678        AAAACAAGAAAATCAATTTAAATTTCAGAAGGAATAAATGTGGTTATAGAAGTGGATTTA      1318
DSM-29679        AAAACAAGAAAATCAATTTAAATTTCAGAAGGAATAAATGTGGTTATAGAAGTGGATTTA      1318
DSM-29683        AAAACAAGAAAATCAATTTAAATTTCAGAAGGAATAAATGTGGTTATAGAAGTGGATTTA      1318
DSM-29684        AAAACAAGAAAATCAATTTAAATTTCAGAAGGAATAAATGTGGTTATAGAAGTGGATTTA      1318
DSM-29685        AAAACAAGAAAATCAATTTAAATTTCAGAAGGAATAAATGTGGTTATAGAAGTGGATTTA      1318
DSM-29686        AAAACAAGAAAATCAATTTAAATTTCAGAAGGAATAAATGTGGTTATAGAAGTGGATTTA      1318
DSM-29089        AAAACAAGAAAATCAATTTAAATTTCAGAAGGAATAAATGTGGTTATAGAAGTGGATTTA      1318
DSM-29689        AAAACAAGAAAATCAATTTAAATTTCAGAAGGAATAAATGTGGTTATAGAAGTGGATTTA      1318
DSM-29692        AAAACAAGAAAATCAATTTAAATTTCAGAAGGAATAAATGTGGTTATAGAAGTGGATTTA      1318
DSM-29091        AAAACAAGAAAATCAATTTAAATTTCAGAAGGAATAAATGTGGTTATAGAAGTGGATTTA      1318
630              AAAACAAGAAAATCAATTTAAATTTCAGAAGGAATAAATGTGGTTATAGAAGTGGATTTA      1318
630-delta-erm    AAAACAAGAAAATCAATTTAAATTTCAGAAGGAATAAATGTGGTTATAGAAGTGGATTTA      1318
DSM-29744        AAAACAAGAAAATCAATTTAAATTTCAGAAGGAATAAATGTGGTTATAGAAGTGGATTTA      1318
DSM-29745        AAAACAAGAAAATCAATTTAAATTTCAGAAGGAATAAATGTGGTTATAGAAGTGGATTTA      1318
DSM-29754        AAAACAAGAAAATCAATTTAAATTTCAGAAGGAATAAATGTGGTTATAGAAGTGGATTTA      1318
DSM-29687        AAAACAAGAAAATCAATTTAAATTTCAGAAGGAATAAATGTGGTTATAGAAGTGGATTTA      1318
DSM-29690        AAAACAAGAAAATCAATTTAAATTTCAGAAGGAATAAATGTGGTTATAGAAGTGGATTTA      1318
DSM-29691        AAAACAAGAAAATCAATTTAAATTTCAGAAGGAATAAATGTGGTTATAGAAGTGGATTTA      1318
DSM-29092        AAAACAAGAAAATCAATTTAAATTTCAGAAGGAATAAATGTGGTTATAGAAGTGGATTTA      1318
DSM-29093        AAAACAAGAAAATCAATTTAAATTTCAGAAGGAATAAATGTGGTTATAGAAGTGGATTTA      1318
DSM-29756        AAAACAAGAAAATCAATTTAAATTTCAGAAGGAATAAATGTGGTTATAGAAGTGGATTTA      1318
DSM-29094        AAAACAAGAAAATCAATTTAAATTTCAGAAGGAATAAATGTGGTTATAGAAGTGGATTTA      1318
2007855          AAAACAAGAAAATCAATTTAAATTTCAGAAGGAATAAATGTGGTTATAGAAGTGGATTTA      1318
BI1              AAAACAAGAAAATCAATTTAAATTTCAGAAGGAATAAATGTGGTTATAGAAGTGGATTTA      1318
DSM-28196        AAAACAAGAAAATCAATTTAAATTTCAGAAGGAATAAATGTGGTTATAGAAGTGGATTTA      1318
DSM-27638        AAAACAAGAAAATCAATTTAAATTTCAGAAGGAATAAATGTGGTTATAGAAGTGGATTTA      1318
DSM-27640        AAAACAAGAAAATCAATTTAAATTTCAGAAGGAATAAATGTGGTTATAGAAGTGGATTTA      1318
DSM-29680        AAAACAAGAAAATCAATTTAAATTTCAGAAGGAATAAATGTGGTTATAGAAGTGGATTTA      1318
R20291           AAAACAAGAAAATCAATTTAAATTTCAGAAGGAATAAATGTGGTTATAGAAGTGGATTTA      1318
DSM-29747        AAAAAAAGAAAATCAATTTAAATTTCAGAAGGAATAAATGTGGTTATAGAAGTGGATTTA      1318
DSM-29752        AAAAAAAGAAAATCAATTTAAATTTCAGAAGGAATAAATGTGGTTATAGAAGTGGATTTA      1318
DSM-29753        AAAAAAAGAAAATCAATTTAAATTTCAGAAGGAATAAATGTGGTTATAGAAGTGGATTTA      1318
M120             AAAAAAAGAAAATCAATTTAAATTTCAGAAGGAATAAATGTGGTTATAGAAGTGGATTTA      1318
                 **** *******************************************************
```

```
M68                TTATCAAAAATAATAATACTAGGAGGTTTTT       1340
CF5                TTATCAAAAATAATAATACTAGGAGGTTTTT       1340
DSM-29095          TTATCAAAAATAATAATACTAGGAGGTTTTT       1349
DSM-29090          TTATCAAAAATAATAATACTAGGAGGTTTTT       1349
DSM-28194          TTATCAAAAATAATAATACTAGGAGGTTTTT       1349
DSM-28197          TTATCAAAAATAATAATACTAGGAGGTTTTT       1349
DSM-1296T          TTATCAAAAATAATAATACTAGGAGGTTTTT       1349
DSM-27639          TTATCAAAAATAATAATACTAGGAGGTTTTT       1349
DSM-29675          TTATCAAAAATAATAATACTAGGAGGTTTTT       1349
DSM-29676          TTATCAAAAATAATAATACTAGGAGGTTTTT       1349
DSM-29677          TTATCAAAAATAATAATACTAGGAGGTTTTT       1349
DSM-29678          TTATCAAAAATAATAATACTAGGAGGTTTTT       1349
DSM-29679          TTATCAAAAATAATAATACTAGGAGGTTTTT       1349
DSM-29683          TTATCAAAAATAATAATACTAGGAGGTTTTT       1349
DSM-29684          TTATCAAAAATAATAATACTAGGAGGTTTTT       1349
DSM-29685          TTATCAAAAATAATAATACTAGGAGGTTTTT       1349
DSM-29686          TTATCAAAAATAATAATACTAGGAGGTTTTT       1349
DSM-29089          TTATCAAAAATAATAATACTAGGAGGTTTTT       1349
DSM-29689          TTATCAAAAATAATAATACTAGGAGGTTTTT       1349
DSM-29692          TTATCAAAAATAATAATACTAGGAGGTTTTT       1349
DSM-29091          TTATCAAAAATAATAATACTAGGAGGTTTTT       1349
630                TTATCAAAAATAATAATACTAGGAGGTTTTT       1349
630-delta-erm      TTATCAAAAATAATAATACTAGGAGGTTTTT       1349
DSM-29744          TTATCAAAAATAATAATACTAGGAGGTTTTT       1349
DSM-29745          TTATCAAAAATAATAATACTAGGAGGTTTTT       1349
DSM-29754          TTATCAAAAATAATAATACTAGGAGGTTTTT       1349
DSM-29687          TTATCAAAAATAATAATACTAGGAGGTTTTT       1349
DSM-29690          TTATCAAAAATAATAATACTAGGAGGTTTTT       1349
DSM-29691          TTATCAAAAATAATAATACTAGGAGGTTTTT       1349
DSM-29092          TTATCAAAAATAATAATACTAGGAGGTTTTT       1349
DSM-29093          TTATCAAAAATAATAATACTAGGAGGTTTTT       1349
DSM-29756          TTATCAAAAATAATAATACTAGGAGGTTTTT       1349
DSM-29094          TTATCAAAAATAATAATACTAGGAGGTTTTT       1349
2007855            TTATCAAAAATAATAGTACTAGGAGGTTTTT       1349
BI1                TTATCAAAAATAATAGTACTAGGAGGTTTTT       1349
DSM-28196          TTATCAAAAATAATAGTACTAGGAGGTTTTT       1349
DSM-27638          TTATCAAAAATAATAGTACTAGGAGGTTTTT       1349
DSM-27640          TTATCAAAAATAATAGTACTAGGAGGTTTTT       1349
DSM-29680          TTATCAAAAATAATAGTACTAGGAGGTTTTT       1349
R20291             TTATCAAAAATAATAGTACTAGGAGGTTTTT       1349
DSM-29747          TTATCAAAAATAATAATACTAGGAGGTTTTT       1349
DSM-29752          TTATCAAAAATAATAATACTAGGAGGTTTTT       1349
DSM-29753          TTATCAAAAATAATAATACTAGGAGGTTTTT       1349
M120               TTATCAAAAATAATAATACTAGGAGGTTTTT       1349
                   *************** ***************
```

(B)

>2007855

ATAAAAATACGTTAAATATATCCTCTTATACTTAAATATATAAAAATAAACAAAATGATACACTACATAAAGTGTTCTATCTAATATGAA
GATTTACCAATAAAAAGGTGAACTATGATGAATGCACAGTAGTTCACCTTTTTATATTTCTAATGGTAACAAAATATTTTTTTATATAAA
CCTAGGAGGCGTTATGAATATGACAATATCTTTTTTATCAGAGCATATATTTATAAAGTTAGTAATTTTAACTATATCATTTGATACATT
ATTAGGATGTTTAAGTGCAATAAAAAGTCGTAAATTTAATTCTAGTTTTGGAATAGATGGAGGAATCAGAAAAGTAGCAATGATAGCATG
TATATTTTTTTTATCAGTAGTTGACATTCTTACAAAGTTTAACTTTTTATTTATGTTACCACAAGATTGTATCAATTTTTTAAGACTAAA
ACATCTTGGAATATCTGAATTTTTCTCTATTTTATTTATTTTATATGAAAGTGTAAGCATATTAAAAAATATGTGCTTATGTGGATTACC
AGTACCTAAGAGATTAAAGGAAAAAATAGCAGTTTTACTAGATGCAATGACAGATGAAATGAATGCTAAGGATGAAAAGTAAGTAATGGT
AGATATAATAAAAATATTAACAAATAAAAAGTGTTATCCAAATAAGAATAGCTGAAAGTTATCATAATTCATGAAACTGATAATGAAAAC
GAGGGAGCAGATGCCAAGAGACACACAAGTATTAAATACATATAATTTCGAAGCAAGTGTTCATTACTATATGGATGACAAGGTAGTATA
TCAAACATTGGTTCACAAAGATGGTGCATGGTCAGTTGGTAAAATCTATTAAGCTACATTAGTTACAGATATCACAAACTATAATAGTTA
AACATAGAAATATGTGTAAATTGTGATGGAAATTATTCAAAAACACAAAAAATACGTGATGAAGGACAAAATGATAGAGAAAATAAGTATC
AAACCTTAATAAATGATTTAATTGATAGTTTAAAAGTTATAGGAAAAATATATAAAGAAATAAAAACATTAAAAAAATATAAGATATGTT
TACAAATTACTATCAGACAATCTCCTTATCTAATAGAAGAGTCAATTAACTAATTGAGTATCTTTAAATTGAAATGTTAGGAAGTGATTT
AAATATGAAAACTTAAATTATAAAAAATCAATATTAATTTATCTTTAAAAAATAGAAAGGAGTGTATAAGATTTATTTTCAAAGTTTAAA
AACAAGAAAATCAATTTAAATTTCAGAAGGAATAAATGTGGTTATAGAAGTGGATTTATTATCAAAAATAATAGTACTAGGAGGTTTTT

>BI1

ATAAAAATACGTTAAATATATCCTCTTATACTTAAATATATAAAAATAAACAAAATGATACACTACATAAAGTGTTCTATCTAATATGAA
GATTTACCAATAAAAAGGTGAACTATGATGAATGCACAGTAGTTCACCTTTTTATATTTCTAATGGTAACAAAATATTTTTTTATATAAA
CCTAGGAGGCGTTATGAATATGACAATATCTTTTTTATCAGAGCATATATTTATAAAGTTAGTAATTTTAACTATATCATTTGATACATT
ATTAGGATGTTTAAGTGCAATAAAAAGTCGTAAATTTAATTCTAGTTTTGGAATAGATGGAGGAATCAGAAAAGTAGCAATGATAGCATG
TATATTTTTTTTATCAGTAGTTGACATTCTTACAAAGTTTAACTTTTTATTTATGTTACCACAAGATTGTATCAATTTTTTAAGACTAAA
ACATCTTGGAATATCTGAATTTTTCTCTATTTTATTTATTTTATATGAAAGTGTAAGCATATTAAAAAATATGTGCTTATGTGGATTACC
AGTACCTAAGAGATTAAAGGAAAAAATAGCAGTTTTACTAGATGCAATGACAGATGAAATGAATGCTAAGGATGAAAAGTAAGTAATGGT
AGATATAATAAAAATATTAACAAATAAAAAGTGTTATCCAAATAAGAATAGCTGAAAGTTATCATAATTCATGAAACTGATAATGAAAAC
GAGGGAGCAGATGCCAAGAGACACACAAGTATTAAATACATATAATTTCGAAGCAAGTGTTCATTACTATATGGATGACAAGGTAGTATA
TCAAACATTGGTTCACAAAGATGGTGCATGGTCAGTTGGTAAAATCTATTAAGCTACATTAGTTACAGATATCACAAACTATAATAGTTA
AACATAGAAATATGTGTAAATTGTGATGGAAATTATTCAAAAACACAAAAAATACGTGATGAAGGACAAAATGATAGAGAAAATAAGTATC
AAACCTTAATAAATGATTTAATTGATAGTTTAAAAGTTATAGGAAAAATATATAAAGAAATAAAAACATTAAAAAAATATAAGATATGTT
TACAAATTACTATCAGACAATCTCCTTATCTAATAGAAGAGTCAATTAACTAATTGAGTATCTTTAAATTGAAATGTTAGGAAGTGATTT
AAATATGAAAACTTAAATTATAAAAAATCAATATTAATTTATCTTTAAAAAATAGAAAGGAGTGTATAAGATTTATTTTCAAAGTTTAAA
AACAAGAAAATCAATTTAAATTTCAGAAGGAATAAATGTGGTTATAGAAGTGGATTTATTATCAAAAATAATAGTACTAGGAGGTTTTT

>DSM-29095

ATAAAAATATGTTAAATATATCCTCTTATACTTAAATATATAAAAATAAACAAAATGATACACTACATAAAGTGTTCTATCTAATATGAA
GATTTACCAATAAAAAGGTGGACTATGATGAATGCACAGTAGTTCACCTTTTTATATTTCTAATGGTAACAAAATATTTTTTTATATAAA
CCTAGGAGGCGTTATGAATATGACAATATCTTTTTTATCAGAGCATATATTTATAAAGTTAGTAATTTTAACTATATCATTTGATACATT
ATTAGGATGTTTAAGTGCAATAAAAAGTCGTAAATTTAATTCTAGTTTTGGAATAGATGGAGGAATCAGAAAAGTAGCAATGATAGCATG
TATATTTTTTTTATCAGTAGTTGACATTCTTACAAAGTTTAACTTTTTATTTATGTTACCACAAGATTGTATCAATTTTTTAAGACTAAA
ACATCTTGGAATATCTGAATTTTTCTCTATTTTATTTATTTTATATGAAAGTGTAAGTATATTAAAAAATATGTGCTTATGTGGATTACC
AGTACCTAAGAGATTAAAGGAAAAAATAGCAATTTTACTAGATGCAATGACAGATGAAATGAATGCTAAGGATGAAAAGTAAGTAATGGT
AGATATAATAAAGATATTAACAAATAAAAAGTGTTATCCAAATAAGAATAGCTGAAAGTTATCATAATTCATGAAACTAATAATGAAAAC
GAGGGAGCAGATGCCAAGAGACACACAAGTATTAAATACATATAATTTCGAAGCAAGTGTTCATTACTATATAGATGACAAGGTAGTATA
TCAAACATTGGTTCACAAAGATGGTGCATGGTCAGTTGGTAAAATCTATTAAGCTACATTAGTTACAGATATCACAAACTATAATAGTTA
AACATAGAAATATGTGTAAATTGTGATGGAAATTATTCAAAAACACAAAAAATACGTGATGAAGGACAAAATGATATAGAAAATAAGTATC
AAACCTTAATAAATGATTTAATTGATAGTTTAAAAGTTATAGGAAAAATATATAAAGAAATAAAAACATTAAAAAAATATAAGATATGTT
TACAAATTACTATCAGACAATCTCCTTATCTAATAGAAGAGTCAATTAACTAATTGAGTATCTTTAAATTGAAATGTTAGGAAGTGATTT
AAATATGAAAACTTAAATTATAAAAAATCAATATTAATTTATTTTTAAAAAATAGAAAGGAGTGTATAAGATTTATTTTCAAAGTTTAAA
AACAAGAAAATCAATTTAAATTTCAGAAGGAATAAATGTGGTTATAGAAGTGGATTTATTATCAAAAATAATAATACTAGGAGGTTTTT

>DSM-29090

ATAAAAATATGTTAAATATATCCTCTTATACTTAAATATATAAAAATAAACAAAATGATACACTACATAAAGTGTTCTATCTAATATGAA
GATTTACCAATAAAAAGGTGGACTATGATGAATGCACAGTAGTTCACCTTTTTATATTTCTAATGGTAACAAAATATTTTTTTATATAAA
CCTAGGAGGCGTTATGAATATGACAATATCTTTTTTATCAGAGCATATATTTATAAAGTTAGTAATTTTAACTATATCATTTGATACATT
ATTAGGATGTTTAAGTGCAATAAAAAGTCGTAAATTTAATTCTAGTTTTGGAATAGATGGAGGAATCAGAAAAGTAGCAATGATAGCATG
TATATTTTTTTTATCAGTAGTTGACATTCTTACAAAGTTTAACTTTTTATTTATGTTACCACAAGATTGTATCAATTTTTTAAGACTAAA
ACATCTTGGAATATCTGAATTTTTCTCTATTTTATTTATTTTATATGAAAGTGTAAGTATATTAAAAAATATGTGCTTATGTGGATTACC
AGTACCTAAGAGATTAAAGGAAAAAATAGCAATTTTACTAGATGCAATGACAGATGAAATGAATGCTAAGGATGAAAAGTAAGTAATGGT
AGATATAATAAAGATATTAACAAATAAAAAGTGTTATCCAAATAAGAATAGCTGAAAGTTATCATAATTCATGAAACTAATAATGAAAAC
GAGGGAGCAGATGCCAAGAGACACACAAGTATTAAATACATATAATTTCGAAGCAAGTGTTCATTACTATATAGATGACAAGGTAGTATA
TCAAACATTGGTTCACAAAGATGGTGCATGGTCAGTTGGTAAAATCTATTAAGCTACATTAGTTACAGATATCACAAACTATAATAGTTA
AACATAGAAATATGTGTAAATTGTGATGGAAATTATTCAAAAACACAAAAATACGTGATGAAGGACAAAATGATATAGAAAATAAGTATC
AAACCTTAATAAATGATTTAATTGATAGTTTAAAAGTTATAGGAAAAATATATAAAGAAATAAAAACATTAAAAAAATATAAGATATGTT
TACAAATTACTATCAGACAATCTCCTTATCTAATAGAAGAGTCAATTAACTAATTGAGTATCTTTAAATTGAAATGTTAGGAAGTGATTT
AAATATGAAAACTTAAATTATAAAAAATCAATATTAATTTATTTTTAAAAAATAGAAAGGAGTGTATAAGATTTATTTTCAAAGTTTAAA
AACAAGAAAATCAATTTAAATTTCAGAAGGAATAAATGTGGTTATAGAAGTGGATTTATTATCAAAAATAATAATACTAGGAGGTTTTT

>DSM-28194

ATAAAAATATGTTAAATATATCCTCTTATACTTAAATATATAAAAATAAACAAAATGATACACTACATAAAGTGTTCTATCTAATATGAA
GATTTACCAATAAAAAGGTGGACTATGATGAATGCACAGTAGTTCACCTTTTTATATTTCTAATGGTAACAAAATATTTTTTTATATAAA
CCTAGGAGGCGTTATGAATATGACAATATCTTTTTTATCAGAGCATATATTTATAAAGTTAGTAATTTTAACTATATCATTTGATACATT
ATTAGGATGTTTAAGTGCAATAAAAAGTCGTAAATTTAATTCTAGTTTTGGAATAGATGGAGGAATCAGAAAAGTAGCAATGATAGCATG
TATATTTTTTTTATCAGTAGTTGACATTCTTACAAAGTTTAACTTTTTATTTATGTTACCACAAGATTGTATCAATTTTTTAAGACTAAA
ACATCTTGGAATATCTGAATTTTTCTCTATTTTATTTATTTTATATGAAAGTGTAAGTATATTAAAAAATATGTGCTTATGTGGATTACC
AGTACCTAAGAGATTAAAGGAAAAAATAGCAATTTTACTAGATGCAATGACAGATGAAATGAATGCTAAGGATGAAAAGTAAGTAATGGT
AGATATAATAAAGATATTAACAAATAAAAAGTGTTATCCAAATAAGAATAGCTGAAAGTTATCATAATTCATGAAACTAATAATGAAAAC
GAGGGAGCAGATGCCAAGAGACACACAAGTATTAAATACATATAATTTCGAAGCAAGTGTTCATTACTATATAGATGACAAGGTAGTATA
TCAAACATTGGTTCACAAAGATGGTGCATGGTCAGTTGGTAAAATCTATTAAGCTACATTAGTTACAGATATCACAAACTATAATAGTTA
AACATAGAAATATGTGTAAATTGTGATGGAAATTATTCAAAAACACAAAAATACGTGATGAAGGACAAAATGATATAGAAAATAAGTATC
AAACCTTAATAAATGATTTAATTGATAGTTTAAAAGTTATAGGAAAAATATATAAAGAAATAAAAACATTAAAAAAATATAAGATATGTT
TACAAATTACTATCAGACAATCTCCTTATCTAATAGAAGAGTCAATTAACTAATTGAGTATCTTTAAATTGAAATGTTAGGAAGTGATTT
AAATATGAAAACTTAAATTATAAAAAATCAATATTAATTTATTTTTAAAAAATAGAAAGGAGTGTATAAGATTTATTTTCAAAGTTTAAA
AACAAGAAAATCAATTTAAATTTCAGAAGGAATAAATGTGGTTATAGAAGTGGATTTATTATCAAAAATAATAATACTAGGAGGTTTTT

>DSM-28196

ATAAAAATACGTTAAATATATCCTCTTATACTTAAATATATAAAAATAAACAAAATGATACACTACATAAAGTGTTCTATCTAATATGAA
GATTTACCAATAAAAAGGTGAACTATGATGAATGCACAGTAGTTCACCTTTTTATATTTCTAATGGTAACAAAATATTTTTTTATATAAA
CCTAGGAGGCGTTATGAATATGACAATATCTTTTTTATCAGAGCATATATTTATAAAGTTAGTAATTTTAACTATATCATTTGATACATT
ATTAGGATGTTTAAGTGCAATAAAAAGTCGTAAATTTAATTCTAGTTTTGGAATAGATGGAGGAATCAGAAAAGTAGCAATGATAGCATG
TATATTTTTTTTATCAGTAGTTGACATTCTTACAAAGTTTAACTTTTTATTTATGTTACCACAAGATTGTATCAATTTTTTAAGACTAAA
ACATCTTGGAATATCTGAATTTTTCTCTATTTTATTTATTTTATATGAAAGTGTAAGCATATTAAAAAATATGTGCTTATGTGGATTACC
AGTACCTAAGAGATTAAAGGAAAAAATAGCAGTTTTACTAGATGCAATGACAGATGAAATGAATGCTAAGGATGAAAAGTAAGTAATGGT
AGATATAATAAAAATATTAACAAATAAAAAGTGTTATCCAAATAAGAATAGCTGAAAGTTATCATAATTCATGAAACTGATAATGAAAAC
GAGGGAGCAGATGCCAAGAGACACACAAGTATTAAATACATATAATTTCGAAGCAAGTGTTCATTACTATATGGATGACAAGGTAGTATA
TCAAACATTGGTTCACAAAGATGGTGCATGGTCAGTTGGTAAAATCTATTAAGCTACATTAGTTACAGATATCACAAACTATAATAGTTA
AACATAGAAATATGTGTAAATTGTGATGGAAATTATTCAAAAACACAAAAATACGTGATGAAGGACAAAATGATAGAGAAAATAAGTATC
AAACCTTAATAAATGATTTAATTGATAGTTTAAAAGTTATAGGAAAAATATATAAAGAAATAAAAACATTAAAAAAATATAAGATATGTT
TACAAATTACTATCAGACAATCTCCTTATCTAATAGAAGAGTCAATTAACTAATTGAGTATCTTTAAATTGAAATGTTAGGAAGTGATTT
AAATATGAAAACTTAAATTATAAAAAATCAATATTAATTTATCTTTAAAAAATAGAAAGGAGTGTATAAGATTTATTTTCAAAGTTTAAA
AACAAGAAAATCAATTTAAATTTCAGAAGGAATAAATGTGGTTATAGAAGTGGATTTATTATCAAAAATAATAGTACTAGGAGGTTTTT

>DSM-28197

ATAAAAATATGTTAAATATATCCTCTTATACTTAAATATATAAAAATAAACAAAATGATACACTACATAAAGTGTTCTATCTAATATGAA
GATTTACCAATAAAAAGGTGGACTATGATGAATGCACAGTAGTTCACCTTTTTATATTTCTAATGGTAACAAAATATTTTTTTATATAAA
CCTAGGAGGCGTTATGAATATGACAATATCTTTTTTATCAGAGCATATATTTATAAAGTTAGTAATTTTAACTATATCATTTGATACATT
ATTAGGATGTTTAAGTGCAATAAAAAGTCGTAAATTTAATTCTAGTTTTGGAATAGATGGAGGAATCAGAAAAGTAGCAATGATAGCATG
TATATTTTTTTTATCAGTAGTTGACATTCTTACAAAGTTTAACTTTTTATTTATGTTACCACAAGATTGTATCAATTTTTTAAGACTAAA
ACATCTTGGAATATCTGAATTTTTCTCTATTTTATTTATTTTATATGAAAGTGTAAGTATATTAAAAAATATGTGCTTATGTGGATTACC
AGTACCTAAGAGATTAAAGGAAAAAATAGCAATTTTACTAGATGCAATGACAGATGAAATGAATGCTAAGGATGAAAAGTAAGTAATGGT
AGATATAATAAAGATATTAACAAATAAAAAGTGTTATCCAAATAAGAATAGCTGAAAGTTATCATAATTCATGAAACTAATAATGAAAAC
GAGGGAGCAGATGCCAAGAGACACACAAGTATTAAATACATATAATTTCGAAGCAAGTGTTCATTACTATATAGATGACAAGGTAGTATA
TCAAACATTGGTTCACAAAGATGGTGCATGGTCAGTTGGTAAAATCTATTAAGCTACATTAGTTACAGATATCACAAACTATAATAGTTA
AACATAGAAATATGTGTAAATTGTGATGGAAATTATTCAAAAACACAAAAATACGTGATGAAGGACAAAATGATATAGAAAATAAGTATC
AAACCTTAATAAATGATTTAATTGATAGTTTAAAAGTTATAGGAAAAATATATAAAGAAATAAAAACATTAAAAAAATATAAGATATGTT
TACAAATTACTATCAGACAATCTCCTTATCTAATAGAAGAGTCAATTAACTAATTGAGTATCTTTAAATTGAAATGTTAGGAAGTGATTT
AAATATGAAAACTTAAATTATAAAAAATCAATATTAATTTATTTTTAAAAAATAGAAAGGAGTGTATAAGATTTATTTTCAAAGTTTAAA
AACAAGAAAATCAATTTAAATTTCAGAAGGAATAAATGTGGTTATAGAAGTGGATTTATTATCAAAAATAATAATACTAGGAGGTTTTT

>DSM-27638

ATAAAAATACGTTAAATATATCCTCTTATACTTAAATATATAAAAATAAACAAAATGATACACTACATAAAGTGTTCTATCTAATATGAA
GATTTACCAATAAAAAGGTGAACTATGATGAATGCACAGTAGTTCACCTTTTTATATTTCTAATGGTAACAAAATATTTTTTTATATAAA
CCTAGGAGGCGTTATGAATATGACAATATCTTTTTTATCAGAGCATATATTTATAAAGTTAGTAATTTTAACTATATCATTTGATACATT
ATTAGGATGTTTAAGTGCAATAAAAAGTCGTAAATTTAATTCTAGTTTTGGAATAGATGGAGGAATCAGAAAAGTAGCAATGATAGCATG
TATATTTTTTTTATCAGTAGTTGACATTCTTACAAAGTTTAACTTTTTATTTATGTTACCACAAGATTGTATCAATTTTTTAAGACTAAA
ACATCTTGGAATATCTGAATTTTTCTCTATTTTATTTATTTTATATGAAAGTGTAAGCATATTAAAAAATATGTGCTTATGTGGATTACC
AGTACCTAAGAGATTAAAGGAAAAAATAGCAGTTTTACTAGATGCAATGACAGATGAAATGAATGCTAAGGATGAAAAGTAAGTAATGGT
AGATATAATAAAAATATTAACAAATAAAAAGTGTTATCCAAATAAGAATAGCTGAAAGTTATCATAATTCATGAAACTGATAATGAAAAC
GAGGGAGCAGATGCCAAGAGACACACAAGTATTAAATACATATAATTTCGAAGCAAGTGTTCATTACTATATGGATGACAAGGTAGTATA
TCAAACATTGGTTCACAAAGATGGTGCATGGTCAGTTGGTAAAATCTATTAAGCTACATTAGTTACAGATATCACAAACTATAATAGTTA
AACATAGAAATATGTGTAAATTGTGATGGAAATTATTCAAAAACACAAAAATACGTGATGAAGGACAAAATGATAGAGAAAATAAGTATC
AAACCTTAATAAATGATTTAATTGATAGTTTAAAAGTTATAGGAAAAATATATAAAGAAATAAAAACATTAAAAAAATATAAGATATGTT
TACAAATTACTATCAGACAATCTCCTTATCTAATAGAAGAGTCAATTAACTAATTGAGTATCTTTAAATTGAAATGTTAGGAAGTGATTT
AAATATGAAAACTTAAATTATAAAAAATCAATATTAATTTATCTTTAAAAAATAGAAAGGAGTGTATAAGATTTATTTTCAAAGTTTAAA

>DSM-1296T

ATAAAAATATGTTAAATATATCCTCTTATACTTAAATATATAAAAATAAACAAAATGATACACTACATAAAGTGTTCTATCTAATATGAA
GATTTACCAATAAAAAGGTGGACTATGATGAATGCACAGTAGTTCACCTTTTTATATTTCTAATGGTAACAAAATATTTTTTTATATAAA
CCTAGGAGGCGTTATGAATATGACAATATCTTTTTTATCAGAGCATATATTTATAAAGTTAGTAATTTTAACTATATCATTTGATACATT
ATTAGGATGTTTAAGTGCAATAAAAAGTCGTAAATTTAATTCTAGTTTTGGAATAGATGGAGGAATCAGAAAAGTAGCAATGATAGCATG
TATATTTTTTTTATCAGTAGTTGACATTCTTACAAAGTTTAACTTTTTATTTATGTTACCACAAGATTGTATCAATTTTTTAAGACTAAA
ACATCTTGGAATATCTGAATTTTTCTCTATTTTATTTATTTTATATGAAAGTGTAAGTATATTAAAAAATATGTGCTTATGTGGATTACC
AGTACCTAAGAGATTAAAGGAAAAAATAGCAATTTTACTAGATGCAATGACAGATGAAATGAATGCTAAGGATGAAAAGTAAGTAATGGT
AGATATAATAAAGATATTAACAAATAAAAAGTGTTATCCAAATAAGAATAGCTGAAAGTTATCATAATTCATGAAACTAATAATGAAAAC
GAGGGAGCAGATGCCAAGAGACACACAAGTATTAAATACATATAATTTCGAAGCAAGTGTTCATTACTATATAGATGACAAGGTAGTATA
TCAAACATTGGTTCACAAAGATGGTGCATGGTCAGTTGGTAAAATCTATTAAGCTACATTAGTTACAGATATCACAAACTATAATAGTTA
AACATAGAAATATGTGTAAATTGTGATGGAAATTATTCAAAAACACAAAAATACGTGATGAAGGACAAAATGATATAGAAAATAAGTATC
AAACCTTAATAAATGATTTAATTGATAGTTTAAAAGTTATAGGAAAAATATATAAAGAAATAAAAACATTAAAAAAATATAAGATATGTT
TACAAATTACTATCAGACAATCTCCTTATCTAATAGAAGAGTCAATTAACTAATTGAGTATCTTTAAATTGAAATGTTAGGAAGTGATTT
AAATATGAAAACTTAAATTATAAAAAATCAATATTAATTTATTTTTAAAAAATAGAAAGGAGTGTATAAGATTTATTTTCAAAGTTTAAA
AACAAGAAAATCAATTTAAATTTCAGAAGGAATAAATGTGGTTATAGAAGTGGATTTATTATCAAAAATAATAATACTAGGAGGTTTTT

>DSM-27639

ATAAAAATATGTTAAATATATCCTCTTATACTTAAATATATAAAAATAAACAAAATGATACACTACATAAAGTGTTCTATCTAATATGAA
GATTTACCAATAAAAAGGTGGACTATGATGAATGCACAGTAGTTCACCTTTTTATATTTCTAATGGTAACAAAATATTTTTTTATATAAA
CCTAGGAGGCGTTATGAATATGACAATATCTTTTTTATCAGAGCATATATTTATAAAGTTAGTAATTTTAACTATATCATTTGATACATT
ATTAGGATGTTTAAGTGCAATAAAAAGTCGTAAATTTAATTCTAGTTTTGGAATAGATGGAGGAATCAGAAAAGTAGCAATGATAGCATG
TATATTTTTTTTATCAGTAGTTGACATTCTTACAAAGTTTAACTTTTTATTTATGTTACCACAAGATTGTATCAATTTTTTAAGACTAAA
ACATCTTGGAATATCTGAATTTTTCTCTATTTTATTTATTTTATATGAAAGTGTAAGTATATTAAAAAATATGTGCTTATGTGGATTACC
AGTACCTAAGAGATTAAAGGAAAAAATAGCAATTTTACTAGATGCAATGACAGATGAAATGAATGCTAAGGATGAAAAGTAAGTAATGGT
AGATATAATAAAGATATTAACAAATAAAAAGTGTTATCCAAATAAGAATAGCTGAAAGTTATCATAATTCATGAAACTAATAATGAAAAC
GAGGGAGCAGATGCCAAGAGACACACAAGTATTAAATACATATAATTTCGAAGCAAGTGTTCATTACTATATAGATGACAAGGTAGTATA
TCAAACATTGGTTCACAAAGATGGTGCATGGTCAGTTGGTAAAATCTATTAAGCTACATTAGTTACAGATATCACAAACTATAATAGTTA
AACATAGAAATATGTGTAAATTGTGATGGAAATTATTCAAAAACACAAAAATACGTGATGAAGGACAAAATGATATAGAAAATAAGTATC
AAACCTTAATAAATGATTTAATTGATAGTTTAAAAGTTATAGGAAAAATATATAAAGAAATAAAAACATTAAAAAAATATAAGATATGTT
TACAAATTACTATCAGACAATCTCCTTATCTAATAGAAGAGTCAATTAACTAATTGAGTATCTTTAAATTGAAATGTTAGGAAGTGATTT
AAATATGAAAACTTAAATTATAAAAAATCAATATTAATTTATTTTTAAAAAATAGAAAGGAGTGTATAAGATTTATTTTCAAAGTTTAAA
AACAAGAAAATCAATTTAAATTTCAGAAGGAATAAATGTGGTTATAGAAGTGGATTTATTATCAAAAATAATAATACTAGGAGGTTTTT

>DSM-29675

ATAAAAATATGTTAAATATATCCTCTTATACTTAAATATATAAAAATAAACAAAATGATACACTACATAAAGTGTTCTATCTAATATGAA
GATTTACCAATAAAAAGGTGGACTATGATGAATGCACAGTAGTTCACCTTTTTATATTTCTAATGGTAACAAAATATTTTTTTATATAAA
CCTAGGAGGCGTTATGAATATGACAATATCTTTTTTATCAGAGCATATATTTATAAAGTTAGTAATTTTAACTATATCATTTGATACATT
ATTAGGATGTTTAAGTGCAATAAAAAGTCGTAAATTTAATTCTAGTTTTGGAATAGATGGAGGAATCAGAAAAGTAGCAATGATAGCATG
TATATTTTTTTTATCAGTAGTTGACATTCTTACAAAGTTTAACTTTTTATTTATGTTACCACAAGATTGTATCAATTTTTTAAGACTAAA
ACATCTTGGAATATCTGAATTTTTCTCTATTTTATTTATTTTATATGAAAGTGTAAGTATATTAAAAAATATGTGCTTATGTGGATTACC
AGTACCTAAGAGATTAAAGGAAAAAATAGCAATTTTACTAGATGCAATGACAGATGAAATGAATGCTAAGGATGAAAAGTAAGTAATGGT
AGATATAATAAAGATATTAACAAATAAAAAGTGTTATCCAAATAAGAATAGCTGAAAGTTATCATAATTCATGAAACTAATAATGAAAAC
GAGGGAGCAGATGCCAAGAGACACACAAGTATTAAATACATATAATTTCGAAGCAAGTGTTCATTACTATATAGATGACAAGGTAGTATA
TCAAACATTGGTTCACAAAGATGGTGCATGGTCAGTTGGTAAAATCTATTAAGCTACATTAGTTACAGATATCACAAACTATAATAGTTA
AACATAGAAATATGTGTAAATTGTGATGGAAATTATTCAAAAACACAAAAATACGTGATGAAGGACAAAATGATATAGAAAATAAGTATC
AAACCTTAATAAATGATTTAATTGATAGTTTAAAAGTTATAGGAAAAATATATAAAGAAATAAAAACATTAAAAAAATATAAGATATGTT
TACAAATTACTATCAGACAATCTCCTTATCTAATAGAAGAGTCAATTAACTAATTGAGTATCTTTAAATTGAAATGTTAGGAAGTGATTT
AAATATGAAAACTTAAATTATAAAAAATCAATATTAATTTATTTTTAAAAAATAGAAAGGAGTGTATAAGATTTATTTTCAAAGTTTAAA
AACAAGAAAATCAATTTAAATTTCAGAAGGAATAAATGTGGTTATAGAAGTGGATTTATTATCAAAAATAATAATACTAGGAGGTTTTT

>DSM-29676

ATAAAAATATGTTAAATATATCCTCTTATACTTAAATATATAAAAATAAACAAAATGATACACTACATAAAGTGTTCTATCTAATATGAA
GATTTACCAATAAAAAGGTGGACTATGATGAATGCACAGTAGTTCACCTTTTTATATTTCTAATGGTAACAAAATATTTTTTTATATAAA
CCTAGGAGGCGTTATGAATATGACAATATCTTTTTTATCAGAGCATATATTTATAAAGTTAGTAATTTTAACTATATCATTTGATACATT
ATTAGGATGTTTAAGTGCAATAAAAAGTCGTAAATTTAATTCTAGTTTTGGAATAGATGGAGGAATCAGAAAAGTAGCAATGATAGCATG
TATATTTTTTTTATCAGTAGTTGACATTCTTACAAAGTTTAACTTTTTATTTATGTTACCACAAGATTGTATCAATTTTTTAAGACTAAA
ACATCTTGGAATATCTGAATTTTTCTCTATTTTATTTATTTTATATGAAAGTGTAAGTATATTAAAAAATATGTGCTTATGTGGATTACC
AGTACCTAAGAGATTAAAGGAAAAAATAGCAATTTTACTAGATGCAATGACAGATGAAATGAATGCTAAGGATGAAAAGTAAGTAATGGT
AGATATAATAAAGATATTAACAAATAAAAAGTGTTATCCAAATAAGAATAGCTGAAAGTTATCATAATTCATGAAACTAATAATGAAAAC
GAGGGAGCAGATGCCAAGAGACACACAAGTATTAAATACATATAATTTCGAAGCAAGTGTTCATTACTATATAGATGACAAGGTAGTATA
TCAAACATTGGTTCACAAAGATGGTGCATGGTCAGTTGGTAAAATCTATTAAGCTACATTAGTTACAGATATCACAAACTATAATAGTTA
AACATAGAAATATGTGTAAATTGTGATGGAAATTATTCAAAAACACAAAAATACGTGATGAAGGACAAAATGATATAGAAAATAAGTATC
AAACCTTAATAAATGATTTAATTGATAGTTTAAAAGTTATAGGAAAAATATATAAAGAAATAAAAACATTAAAAAAATATAAGATATGTT
TACAAATTACTATCAGACAATCTCCTTATCTAATAGAAGAGTCAATTAACTAATTGAGTATCTTTAAATTGAAATGTTAGGAAGTGATTT
AAATATGAAAACTTAAATTATAAAAAATCAATATTAATTTATTTTTAAAAAATAGAAAGGAGTGTATAAGATTTATTTTCAAAGTTTAAA
AACAAGAAAATCAATTTAAATTTCAGAAGGAATAAATGTGGTTATAGAAGTGGATTTATTATCAAAAATAATAATACTAGGAGGTTTTT

>DSM-29677

ATAAAAATATGTTAAATATATCCTCTTATACTTAAATATATAAAAATAAACAAAATGATACACTACATAAAGTGTTCTATCTAATATGAA
GATTTACCAATAAAAAGGTGGACTATGATGAATGCACAGTAGTTCACCTTTTTATATTTCTAATGGTAACAAAATATTTTTTTATATAAA
CCTAGGAGGCGTTATGAATATGACAATATCTTTTTTATCAGAGCATATATTTATAAAGTTAGTAATTTTAACTATATCATTTGATACATT
ATTAGGATGTTTAAGTGCAATAAAAAGTCGTAAATTTAATTCTAGTTTTGGAATAGATGGAGGAATCAGAAAAGTAGCAATGATAGCATG
TATATTTTTTTTATCAGTAGTTGACATTCTTACAAAGTTTAACTTTTTATTTATGTTACCACAAGATTGTATCAATTTTTTAAGACTAAA
ACATCTTGGAATATCTGAATTTTTCTCTATTTTATTTATTTTATATGAAAGTGTAAGTATATTAAAAAATATGTGCTTATGTGGATTACC
AGTACCTAAGAGATTAAAGGAAAAAATAGCAATTTTACTAGATGCAATGACAGATGAAATGAATGCTAAGGATGAAAAGTAAGTAATGGT
AGATATAATAAAGATATTAACAAATAAAAAGTGTTATCCAAATAAGAATAGCTGAAAGTTATCATAATTCATGAAACTAATAATGAAAAC
GAGGGAGCAGATGCCAAGAGACACACAAGTATTAAATACATATAATTTCGAAGCAAGTGTTCATTACTATATAGATGACAAGGTAGTATA
TCAAACATTGGTTCACAAAGATGGTGCATGGTCAGTTGGTAAAATCTATTAAGCTACATTAGTTACAGATATCACAAACTATAATAGTTA
AACATAGAAATATGTGTAAATTGTGATGGAAATTATTCAAAAACACAAAAATACGTGATGAAGGACAAAATGATATAGAAAATAAGTATC
AAACCTTAATAAATGATTTAATTGATAGTTTAAAAGTTATAGGAAAAATATATAAAGAAATAAAAACATTAAAAAAATATAAGATATGTT
TACAAATTACTATCAGACAATCTCCTTATCTAATAGAAGAGTCAATTAACTAATTGAGTATCTTTAAATTGAAATGTTAGGAAGTGATTT
AAATATGAAAACTTAAATTATAAAAAATCAATATTAATTTATTTTTAAAAAATAGAAAGGAGTGTATAAGATTTATTTTCAAAGTTTAAA
AACAAGAAAATCAATTTAAATTTCAGAAGGAATAAATGTGGTTATAGAAGTGGATTTATTATCAAAAATAATAATACTAGGAGGTTTTT

>DSM-27640

ATAAAAATACGTTAAATATATCCTCTTATACTTAAATATATAAAAATAAACAAAATGATACACTACATAAAGTGTTCTATCTAATATGAA
GATTTACCAATAAAAAGGTGAACTATGATGAATGCACAGTAGTTCACCTTTTTATATTTCTAATGGTAACAAAATATTTTTTTATATAAA
CCTAGGAGGCGTTATGAATATGACAATATCTTTTTTATCAGAGCATATATTTATAAAGTTAGTAATTTTAACTATATCATTTGATACATT
ATTAGGATGTTTAAGTGCAATAAAAAGTCGTAAATTTAATTCTAGTTTTGGAATAGATGGAGGAATCAGAAAAGTAGCAATGATAGCATG
TATATTTTTTTTATCAGTAGTTGACATTCTTACAAAGTTTAACTTTTTATTTATGTTACCACAAGATTGTATCAATTTTTTAAGACTAAA
ACATCTTGGAATATCTGAATTTTTCTCTATTTTATTTATTTTATATGAAAGTGTAAGCATATTAAAAAATATGTGCTTATGTGGATTACC
AGTACCTAAGAGATTAAAGGAAAAAATAGCAGTTTTACTAGATGCAATGACAGATGAAATGAATGCTAAGGATGAAAAGTAAGTAATGGT
AGATATAATAAAAATATTAACAAATAAAAAGTGTTATCCAAATAAGAATAGCTGAAAGTTATCATAATTCATGAAACTGATAATGAAAAC
GAGGGAGCAGATGCCAAGAGACACACAAGTATTAAATACATATAATTTCGAAGCAAGTGTTCATTACTATATGGATGACAAGGTAGTATA
TCAAACATTGGTTCACAAAGATGGTGCATGGTCAGTTGGTAAAATCTATTAAGCTACATTAGTTACAGATATCACAAACTATAATAGTTA
AACATAGAAATATGTGTAAATTGTGATGGAAATTATTCAAAAACACAAAAATACGTGATGAAGGACAAAATGATAGAGAAAATAAGTATC
AAACCTTAATAAATGATTTAATTGATAGTTTAAAAGTTATAGGAAAAATATATAAAGAAATAAAAACATTAAAAAAATATAAGATATGTT
TACAAATTACTATCAGACAATCTCCTTATCTAATAGAAGAGTCAATTAACTAATTGAGTATCTTTAAATTGAAATGTTAGGAAGTGATTT
AAATATGAAAACTTAAATTATAAAAAATCAATATTAATTTATCTTTAAAAAATAGAAAGGAGTGTATAAGATTTATTTTCAAAGTTTAAA
AACAAGAAAATCAATTTAAATTTCAGAAGGAATAAATGTGGTTATAGAAGTGGATTTATTATCAAAAATAATAGTACTAGGAGGTTTTT

>DSM-29678

ATAAAAATATGTTAAATATATCCTCTTATACTTAAATATATAAAAATAAACAAAATGATACACTACATAAAGTGTTCTATCTAATATGAA
GATTTACCAATAAAAAGGTGGACTATGATGAATGCACAGTAGTTCACCTTTTTATATTTCTAATGGTAACAAAATATTTTTTTATATAAA
CCTAGGAGGCGTTATGAATATGACAATATCTTTTTTATCAGAGCATATATTTATAAAGTTAGTAATTTTAACTATATCATTTGATACATT
ATTAGGATGTTTAAGTGCAATAAAAAGTCGTAAATTTAATTCTAGTTTTGGAATAGATGGAGGAATCAGAAAAGTAGCAATGATAGCATG
TATATTTTTTTTATCAGTAGTTGACATTCTTACAAAGTTTAACTTTTTATTTATGTTACCACAAGATTGTATCAATTTTTTAAGACTAAA
ACATCTTGGAATATCTGAATTTTTCTCTATTTTATTTATTTTATATGAAAGTGTAAGTATATTAAAAAATATGTGCTTATGTGGATTACC
AGTACCTAAGAGATTAAAGGAAAAAATAGCAATTTTACTAGATGCAATGACAGATGAAATGAATGCTAAGGATGAAAAGTAAGTAATGGT
AGATATAATAAAGATATTAACAAATAAAAAGTGTTATCCAAATAAGAATAGCTGAAAGTTATCATAATTCATGAAACTAATAATGAAAAC
GAGGGAGCAGATGCCAAGAGACACACAAGTATTAAATACATATAATTTCGAAGCAAGTGTTCATTACTATATAGATGACAAGGTAGTATA
TCAAACATTGGTTCACAAAGATGGTGCATGGTCAGTTGGTAAAATCTATTAAGCTACATTAGTTACAGATATCACAAACTATAATAGTTA
AACATAGAAATATGTGTAAATTGTGATGGAAATTATTCAAAAACACAAAAATACGTGATGAAGGACAAAATGATATAGAAAATAAGTATC
AAACCTTAATAAATGATTTAATTGATAGTTTAAAAGTTATAGGAAAAATATATAAAGAAATAAAAACATTAAAAAAATATAAGATATGTT
TACAAATTACTATCAGACAATCTCCTTATCTAATAGAAGAGTCAATTAACTAATTGAGTATCTTTAAATTGAAATGTTAGGAAGTGATTT
AAATATGAAAACTTAAATTATAAAAAATCAATATTAATTTATTTTTAAAAAATAGAAAGGAGTGTATAAGATTTATTTTCAAAGTTTAAA
AACAAGAAAATCAATTTAAATTTCAGAAGGAATAAATGTGGTTATAGAAGTGGATTTATTATCAAAAATAATAATACTAGGAGGTTTTT

>DSM-29679

ATAAAAATATGTTAAATATATCCTCTTATACTTAAATATATAAAAATAAACAAAATGATACACTACATAAAGTGTTCTATCTAATATGAA
GATTTACCAATAAAAAGGTGGACTATGATGAATGCACAGTAGTTCACCTTTTTATATTTCTAATGGTAACAAAATATTTTTTTATATAAA
CCTAGGAGGCGTTATGAATATGACAATATCTTTTTTATCAGAGCATATATTTATAAAGTTAGTAATTTTAACTATATCATTTGATACATT
ATTAGGATGTTTAAGTGCAATAAAAAGTCGTAAATTTAATTCTAGTTTTGGAATAGATGGAGGAATCAGAAAAGTAGCAATGATAGCATG
TATATTTTTTTTATCAGTAGTTGACATTCTTACAAAGTTTAACTTTTTATTTATGTTACCACAAGATTGTATCAATTTTTTAAGACTAAA
ACATCTTGGAATATCTGAATTTTTCTCTATTTTATTTATTTTATATGAAAGTGTAAGTATATTAAAAAATATGTGCTTATGTGGATTACC
AGTACCTAAGAGATTAAAGGAAAAAATAGCAATTTTACTAGATGCAATGACAGATGAAATGAATGCTAAGGATGAAAAGTAAGTAATGGT
AGATATAATAAAGATATTAACAAATAAAAAGTGTTATCCAAATAAGAATAGCTGAAAGTTATCATAATTCATGAAACTAATAATGAAAAC
GAGGGAGCAGATGCCAAGAGACACACAAGTATTAAATACATATAATTTCGAAGCAAGTGTTCATTACTATATAGATGACAAGGTAGTATA
TCAAACATTGGTTCACAAAGATGGTGCATGGTCAGTTGGTAAAATCTATTAAGCTACATTAGTTACAGATATCACAAACTATAATAGTTA
AACATAGAAATATGTGTAAATTGTGATGGAAATTATTCAAAAACACAAAAATACGTGATGAAGGACAAAATGATATAGAAAATAAGTATC
AAACCTTAATAAATGATTTAATTGATAGTTTAAAAGTTATAGGAAAAATATATAAAGAAATAAAAACATTAAAAAAATATAAGATATGTT
TACAAATTACTATCAGACAATCTCCTTATCTAATAGAAGAGTCAATTAACTAATTGAGTATCTTTAAATTGAAATGTTAGGAAGTGATTT
AAATATGAAAACTTAAATTATAAAAAATCAATATTAATTTATTTTTAAAAAATAGAAAGGAGTGTATAAGATTTATTTTCAAAGTTTAAA
AACAAGAAAATCAATTTAAATTTCAGAAGGAATAAATGTGGTTATAGAAGTGGATTTATTATCAAAAATAATAATACTAGGAGGTTTTT

>DSM-29680

ATAAAAATACGTTAAATATATCCTCTTATACTTAAATATATAAAAATAAACAAAATGATACACTACATAAAGTGTTCTATCTAATATGAA
GATTTACCAATAAAAAGGTGAACTATGATGAATGCACAGTAGTTCACCTTTTTATATTTCTAATGGTAACAAAATATTTTTTTATATAAA
CCTAGGAGGCGTTATGAATATGACAATATCTTTTTTATCAGAGCATATATTTATAAAGTTAGTAATTTTAACTATATCATTTGATACATT
ATTAGGATGTTTAAGTGCAATAAAAAGTCGTAAATTTAATTCTAGTTTTGGAATAGATGGAGGAATCAGAAAAGTAGCAATGATAGCATG
TATATTTTTTTTATCAGTAGTTGACATTCTTACAAAGTTTAACTTTTTATTTATGTTACCACAAGATTGTATCAATTTTTTAAGACTAAA
ACATCTTGGAATATCTGAATTTTTCTCTATTTTATTTATTTTATATGAAAGTGTAAGCATATTAAAAAATATGTGCTTATGTGGATTACC
AGTACCTAAGAGATTAAAGGAAAAAATAGCAGTTTTACTAGATGCAATGACAGATGAAATGAATGCTAAGGATGAAAAGTAAGTAATGGT
AGATATAATAAAAATATTAACAAATAAAAAGTGTTATCCAAATAAGAATAGCTGAAAGTTATCATAATTCATGAAACTGATAATGAAAAC
GAGGGAGCAGATGCCAAGAGACACACAAGTATTAAATACATATAATTTCGAAGCAAGTGTTCATTACTATATGGATGACAAGGTAGTATA
TCAAACATTGGTTCACAAAGATGGTGCATGGTCAGTTGGTAAAATCTATTAAGCTACATTAGTTACAGATATCACAAACTATAATAGTTA
AACATAGAAATATGTGTAAATTGTGATGGAAATTATTCAAAAACACAAAAATACGTGATGAAGGACAAAATGATAGAGAAAATAAGTATC
AAACCTTAATAAATGATTTAATTGATAGTTTAAAAGTTATAGGAAAAATATATAAAGAAATAAAAACATTAAAAAAATATAAGATATGTT
TACAAATTACTATCAGACAATCTCCTTATCTAATAGAAGAGTCAATTAACTAATTGAGTATCTTTAAATTGAAATGTTAGGAAGTGATTT
AAATATGAAAACTTAAATTATAAAAAATCAATATTAATTTATCTTTAAAAAATAGAAAGGAGTGTATAAGATTTATTTTCAAAGTTTAAA
AACAAGAAAATCAATTTAAATTTCAGAAGGAATAAATGTGGTTATAGAAGTGGATTTATTATCAAAAATAATAGTACTAGGAGGTTTTT

>DSM-29683

ATAAAAATATGTTAAATATATCCTCTTATACTTAAATATATAAAAATAAACAAAATGATACACTACATAAAGTGTTCTATCTAATATGAA
GATTTACCAATAAAAAGGTGGACTATGATGAATGCACAGTAGTTCACCTTTTTATATTTCTAATGGTAACAAAATATTTTTTTATATAAA
CCTAGGAGGCGTTATGAATATGACAATATCTTTTTTATCAGAGCATATATTTATAAAGTTAGTAATTTTAACTATATCATTTGATACATT
ATTAGGATGTTTAAGTGCAATAAAAAGTCGTAAATTTAATTCTAGTTTTGGAATAGATGGAGGAATCAGAAAAGTAGCAATGATAGCATG
TATATTTTTTTTATCAGTAGTTGACATTCTTACAAAGTTTAACTTTTTATTTATGTTACCACAAGATTGTATCAATTTTTTAAGACTAAA
ACATCTTGGAATATCTGAATTTTTCTCTATTTTATTTATTTTATATGAAAGTGTAAGTATATTAAAAAATATGTGCTTATGTGGATTACC
AGTACCTAAGAGATTAAAGGAAAAAATAGCAATTTTACTAGATGCAATGACAGATGAAATGAATGCTAAGGATGAAAAGTAAGTAATGGT
AGATATAATAAAGATATTAACAAATAAAAAGTGTTATCCAAATAAGAATAGCTGAAAGTTATCATAATTCATGAAACTAATAATGAAAAC
GAGGGAGCAGATGCCAAGAGACACACAAGTATTAAATACATATAATTTCGAAGCAAGTGTTCATTACTATATAGATGACAAGGTAGTATA
TCAAACATTGGTTCACAAAGATGGTGCATGGTCAGTTGGTAAAATCTATTAAGCTACATTAGTTACAGATATCACAAACTATAATAGTTA
AACATAGAAATATGTGTAAATTGTGATGGAAATTATTCAAAAACACAAAAATACGTGATGAAGGACAAAATGATATAGAAAATAAGTATC
AAACCTTAATAAATGATTTAATTGATAGTTTAAAAGTTATAGGAAAAATATATAAAGAAATAAAAACATTAAAAAAATATAAGATATGTT
TACAAATTACTATCAGACAATCTCCTTATCTAATAGAAGAGTCAATTAACTAATTGAGTATCTTTAAATTGAAATGTTAGGAAGTGATTT
AAATATGAAAACTTAAATTATAAAAAATCAATATTAATTTATTTTTAAAAAATAGAAAGGAGTGTATAAGATTTATTTTCAAAGTTTAAA
AACAAGAAAATCAATTTAAATTTCAGAAGGAATAAATGTGGTTATAGAAGTGGATTTATTATCAAAAATAATAATACTAGGAGGTTTTT

>DSM-29684

ATAAAAATATGTTAAATATATCCTCTTATACTTAAATATATAAAAATAAACAAAATGATACACTACATAAAGTGTTCTATCTAATATGAA
GATTTACCAATAAAAAGGTGGACTATGATGAATGCACAGTAGTTCACCTTTTTATATTTCTAATGGTAACAAAATATTTTTTTATATAAA
CCTAGGAGGCGTTATGAATATGACAATATCTTTTTTATCAGAGCATATATTTATAAAGTTAGTAATTTTAACTATATCATTTGATACATT
ATTAGGATGTTTAAGTGCAATAAAAAGTCGTAAATTTAATTCTAGTTTTGGAATAGATGGAGGAATCAGAAAAGTAGCAATGATAGCATG
TATATTTTTTTTATCAGTAGTTGACATTCTTACAAAGTTTAACTTTTTATTTATGTTACCACAAGATTGTATCAATTTTTTAAGACTAAA
ACATCTTGGAATATCTGAATTTTTCTCTATTTTATTTATTTTATATGAAAGTGTAAGTATATTAAAAAATATGTGCTTATGTGGATTACC
AGTACCTAAGAGATTAAAGGAAAAAATAGCAATTTTACTAGATGCAATGACAGATGAAATGAATGCTAAGGATGAAAAGTAAGTAATGGT
AGATATAATAAAGATATTAACAAATAAAAAGTGTTATCCAAATAAGAATAGCTGAAAGTTATCATAATTCATGAAACTAATAATGAAAAC
GAGGGAGCAGATGCCAAGAGACACACAAGTATTAAATACATATAATTTCGAAGCAAGTGTTCATTACTATATAGATGACAAGGTAGTATA
TCAAACATTGGTTCACAAAGATGGTGCATGGTCAGTTGGTAAAATCTATTAAGCTACATTAGTTACAGATATCACAAACTATAATAGTTA
AACATAGAAATATGTGTAAATTGTGATGGAAATTATTCAAAAACACAAAAATACGTGATGAAGGACAAAATGATATAGAAAATAAGTATC
AAACCTTAATAAATGATTTAATTGATAGTTTAAAAGTTATAGGAAAAATATATAAAGAAATAAAAACATTAAAAAAATATAAGATATGTT
TACAAATTACTATCAGACAATCTCCTTATCTAATAGAAGAGTCAATTAACTAATTGAGTATCTTTAAATTGAAATGTTAGGAAGTGATTT
AAATATGAAAACTTAAATTATAAAAAATCAATATTAATTTATTTTTAAAAAATAGAAAGGAGTGTATAAGATTTATTTTCAAAGTTTAAA
AACAAGAAAATCAATTTAAATTTCAGAAGGAATAAATGTGGTTATAGAAGTGGATTTATTATCAAAAATAATAATACTAGGAGGTTTTT

>DSM-29685

ATAAAAATATGTTAAATATATCCTCTTATACTTAAATATATAAAAATAAACAAAATGATACACTACATAAAGTGTTCTATCTAATATGAA
GATTTACCAATAAAAAGGTGGACTATGATGAATGCACAGTAGTTCACCTTTTTATATTTCTAATGGTAACAAAATATTTTTTTATATAAA
CCTAGGAGGCGTTATGAATATGACAATATCTTTTTTATCAGAGCATATATTTATAAAGTTAGTAATTTTAACTATATCATTTGATACATT
ATTAGGATGTTTAAGTGCAATAAAAAGTCGTAAATTTAATTCTAGTTTTGGAATAGATGGAGGAATCAGAAAAGTAGCAATGATAGCATG
TATATTTTTTTTATCAGTAGTTGACATTCTTACAAAGTTTAACTTTTTATTTATGTTACCACAAGATTGTATCAATTTTTTAAGACTAAA
ACATCTTGGAATATCTGAATTTTTCTCTATTTTATTTATTTTATATGAAAGTGTAAGTATATTAAAAAATATGTGCTTATGTGGATTACC
AGTACCTAAGAGATTAAAGGAAAAAATAGCAATTTTACTAGATGCAATGACAGATGAAATGAATGCTAAGGATGAAAAGTAAGTAATGGT
AGATATAATAAAGATATTAACAAATAAAAAGTGTTATCCAAATAAGAATAGCTGAAAGTTATCATAATTCATGAAACTAATAATGAAAAC
GAGGGAGCAGATGCCAAGAGACACACAAGTATTAAATACATATAATTTCGAAGCAAGTGTTCATTACTATATAGATGACAAGGTAGTATA
TCAAACATTGGTTCACAAAGATGGTGCATGGTCAGTTGGTAAAATCTATTAAGCTACATTAGTTACAGATATCACAAACTATAATAGTTA
AACATAGAAATATGTGTAAATTGTGATGGAAATTATTCAAAAACACAAAAATACGTGATGAAGGACAAAATGATATAGAAAATAAGTATC
AAACCTTAATAAATGATTTAATTGATAGTTTAAAAGTTATAGGAAAAATATATAAAGAAATAAAAACATTAAAAAAATATAAGATATGTT
TACAAATTACTATCAGACAATCTCCTTATCTAATAGAAGAGTCAATTAACTAATTGAGTATCTTTAAATTGAAATGTTAGGAAGTGATTT
AAATATGAAAACTTAAATTATAAAAAATCAATATTAATTTATTTTTAAAAAATAGAAAGGAGTGTATAAGATTTATTTTCAAAGTTTAAA
AACAAGAAAATCAATTTAAATTTCAGAAGGAATAAATGTGGTTATAGAAGTGGATTTATTATCAAAAATAATAATACTAGGAGGTTTTT

>DSM-29686

ATAAAAATATGTTAAATATATCCTCTTATACTTAAATATATAAAAATAAACAAAATGATACACTACATAAAGTGTTCTATCTAATATGAA
GATTTACCAATAAAAAGGTGGACTATGATGAATGCACAGTAGTTCACCTTTTTATATTTCTAATGGTAACAAAATATTTTTTTATATAAA
CCTAGGAGGCGTTATGAATATGACAATATCTTTTTTATCAGAGCATATATTTATAAAGTTAGTAATTTTAACTATATCATTTGATACATT
ATTAGGATGTTTAAGTGCAATAAAAAGTCGTAAATTTAATTCTAGTTTTGGAATAGATGGAGGAATCAGAAAAGTAGCAATGATAGCATG
TATATTTTTTTTATCAGTAGTTGACATTCTTACAAAGTTTAACTTTTTATTTATGTTACCACAAGATTGTATCAATTTTTTAAGACTAAA
ACATCTTGGAATATCTGAATTTTTCTCTATTTTATTTATTTTATATGAAAGTGTAAGTATATTAAAAAATATGTGCTTATGTGGATTACC
AGTACCTAAGAGATTAAAGGAAAAAATAGCAATTTTACTAGATGCAATGACAGATGAAATGAATGCTAAGGATGAAAAGTAAGTAATGGT
AGATATAATAAAGATATTAACAAATAAAAAGTGTTATCCAAATAAGAATAGCTGAAAGTTATCATAATTCATGAAACTAATAATGAAAAC
GAGGGAGCAGATGCCAAGAGACACACAAGTATTAAATACATATAATTTCGAAGCAAGTGTTCATTACTATATAGATGACAAGGTAGTATA
TCAAACATTGGTTCACAAAGATGGTGCATGGTCAGTTGGTAAAATCTATTAAGCTACATTAGTTACAGATATCACAAACTATAATAGTTA
AACATAGAAATATGTGTAAATTGTGATGGAAATTATTCAAAAACACAAAAATACGTGATGAAGGACAAAATGATATAGAAAATAAGTATC
AAACCTTAATAAATGATTTAATTGATAGTTTAAAAGTTATAGGAAAAATATATAAAGAAATAAAAACATTAAAAAAATATAAGATATGTT
TACAAATTACTATCAGACAATCTCCTTATCTAATAGAAGAGTCAATTAACTAATTGAGTATCTTTAAATTGAAATGTTAGGAAGTGATTT
AAATATGAAAACTTAAATTATAAAAAATCAATATTAATTTATTTTTAAAAAATAGAAAGGAGTGTATAAGATTTATTTTCAAAGTTTAAA
AACAAGAAAATCAATTTAAATTTCAGAAGGAATAAATGTGGTTATAGAAGTGGATTTATTATCAAAAATAATAATACTAGGAGGTTTTT

>DSM-29687

ATAAAAATATGTTAAATATATCCTCTTATACTTAAATATATAAAAATAAACAAAATGATACACTACATAAAGTGTTCTATCTAATATGAA
GATTTACCAATAAAAAGGTGGACTATGATGAATGCACAGTAGTTCACCTTTTTATATTTCTAATGGTAACAAAATATTTTTTTATATAAA
CCTAGGAGGCGTTATGAATATGACAATATCTTTTTTATCAGAGCATATATTTATAAAGTTAGTAATTTTAACTATATCATTTGATACATT
ATTAGGATGTTTAAGTGCAATAAAAAGTCGTAAATTTAATTCTAGTTTTGGAATAGATGGAGGAATCAGAAAAGTAGCAATGATAGCATG
TATATTTTTTTTATCAGTAGTTGACATTCTTACAAAGTTTAACTTTTTATTTATGTTACCACAAGATTGTATCAATTTTTTAAGACTAAA
ACATCTTGGAATATCTGAATTTTTCTCTATTTTATTTATTTTATATGAAAGTGTAAGTATATTAAAAAATATGTGCTTATGTGGATTACC
AGTACCTAAGAGATTAAAGGAAAAAATAGCAATTTTACTAGATGCAATGACAGATGAAATGAATGCTAAGGATGAAAAGTAAGTAATGGT
AGATATAATAAAGATATTAACAAATAAAAAGTGTTATCCAAATAAGAATAGCTGAAAGTTATCATAATTCATGAAACTAATAATGAAAAC
GAGGGAGCAGATGCCAAGAGACACACAAGTATTAAATACATATAATTTCGAAGCAAGTGTTCATTACTATATAGATGACAAGGTAGTATA
TCAAACATTGGTTCACAAAGATGGTGCATGGTCAGTTGGTAAAATCTATTAAGCTACATTAGTTACAGATATCACAAACTATAATAGTTA
AACATAGAAATATGTGTAAATTGTGATTGAAATTATTCAAAAACACAAAAATACGTGATGAAGGACAAAATGATATAGAAAATAAGTATC
AAACCTTAATAAATGATTTAATTGATAGTTTAAAAGTTATAGGAAAAATATATAAAGAAATAAAAACATTAAAAAAATATAAGATATGTT
TACAAATTACTATCAGACAATCTCCTTATCTAATAGAAGAGTCAATTAACTAATTGAGTATCTTTAAATTGAAATGTTAGGAAGTGATTT
AAATATGAAAACTTAAATTATAAAAAATCAATATTAATTTATTTTTAAAAAATAGAAAGGAGTGTATAAGATTTATTTTCAAAGTTTAAA
AACAAGAAAATCAATTTAAATTTCAGAAGGAATAAATGTGGTTATAGAAGTGGATTTATTATCAAAAATAATAATACTAGGAGGTTTTT

>DSM-29089

ATAAAAATATGTTAAATATATCCTCTTATACTTAAATATATAAAAATAAACAAAATGATACACTACATAAAGTGTTCTATCTAATATGAA
GATTTACCAATAAAAAGGTGGACTATGATGAATGCACAGTAGTTCACCTTTTTATATTTCTAATGGTAACAAAATATTTTTTTATATAAA
CCTAGGAGGCGTTATGAATATGACAATATCTTTTTTATCAGAGCATATATTTATAAAGTTAGTAATTTTAACTATATCATTTGATACATT
ATTAGGATGTTTAAGTGCAATAAAAAGTCGTAAATTTAATTCTAGTTTTGGAATAGATGGAGGAATCAGAAAAGTAGCAATGATAGCATG
TATATTTTTTTTATCAGTAGTTGACATTCTTACAAAGTTTAACTTTTTATTTATGTTACCACAAGATTGTATCAATTTTTTAAGACTAAA
ACATCTTGGAATATCTGAATTTTTCTCTATTTTATTTATTTTATATGAAAGTGTAAGTATATTAAAAAATATGTGCTTATGTGGATTACC
AGTACCTAAGAGATTAAAGGAAAAAATAGCAATTTTACTAGATGCAATGACAGATGAAATGAATGCTAAGGATGAAAAGTAAGTAATGGT
AGATATAATAAAGATATTAACAAATAAAAAGTGTTATCCAAATAAGAATAGCTGAAAGTTATCATAATTCATGAAACTAATAATGAAAAC
GAGGGAGCAGATGCCAAGAGACACACAAGTATTAAATACATATAATTTCGAAGCAAGTGTTCATTACTATATAGATGACAAGGTAGTATA
TCAAACATTGGTTCACAAAGATGGTGCATGGTCAGTTGGTAAAATCTATTAAGCTACATTAGTTACAGATATCACAAACTATAATAGTTA
AACATAGAAATATGTGTAAATTGTGATGGAAATTATTCAAAAACACAAAAATACGTGATGAAGGACAAAATGATATAGAAAATAAGTATC
AAACCTTAATAAATGATTTAATTGATAGTTTAAAAGTTATAGGAAAAATATATAAAGAAATAAAAACATTAAAAAAATATAAGATATGTT
TACAAATTACTATCAGACAATCTCCTTATCTAATAGAAGAGTCAATTAACTAATTGAGTATCTTTAAATTGAAATGTTAGGAAGTGATTT
AAATATGAAAACTTAAATTATAAAAAATCAATATTAATTTATTTTTAAAAAATAGAAAGGAGTGTATAAGATTTATTTTCAAAGTTTAAA
AACAAGAAAATCAATTTAAATTTCAGAAGGAATAAATGTGGTTATAGAAGTGGATTTATTATCAAAAATAATAATACTAGGAGGTTTTT

>DSM-29689

ATAAAAATATGTTAAATATATCCTCTTATACTTAAATATATAAAAATAAACAAAATGATACACTACATAAAGTGTTCTATCTAATATGAA
GATTTACCAATAAAAAGGTGGACTATGATGAATGCACAGTAGTTCACCTTTTTATATTTCTAATGGTAACAAAATATTTTTTTATATAAA
CCTAGGAGGCGTTATGAATATGACAATATCTTTTTTATCAGAGCATATATTTATAAAGTTAGTAATTTTAACTATATCATTTGATACATT
ATTAGGATGTTTAAGTGCAATAAAAAGTCGTAAATTTAATTCTAGTTTTGGAATAGATGGAGGAATCAGAAAAGTAGCAATGATAGCATG
TATATTTTTTTTATCAGTAGTTGACATTCTTACAAAGTTTAACTTTTTATTTATGTTACCACAAGATTGTATCAATTTTTTAAGACTAAA
ACATCTTGGAATATCTGAATTTTTCTCTATTTTATTTATTTTATATGAAAGTGTAAGTATATTAAAAAATATGTGCTTATGTGGATTACC
AGTACCTAAGAGATTAAAGGAAAAAATAGCAATTTTACTAGATGCAATGACAGATGAAATGAATGCTAAGGATGAAAAGTAAGTAATGGT
AGATATAATAAAGATATTAACAAATAAAAAGTGTTATCCAAATAAGAATAGCTGAAAGTTATCATAATTCATGAAACTAATAATGAAAAC
GAGGGAGCAGATGCCAAGAGACACACAAGTATTAAATACATATAATTTCGAAGCAAGTGTTCATTACTATATAGATGACAAGGTAGTATA
TCAAACATTGGTTCACAAAGATGGTGCATGGTCAGTTGGTAAAATCTATTAAGCTACATTAGTTACAGATATCACAAACTATAATAGTTA
AACATAGAAATATGTGTAAATTGTGATGGAAATTATTCAAAAACACAAAAATACGTGATGAAGGACAAAATGATATAGAAAATAAGTATC
AAACCTTAATAAATGATTTAATTGATAGTTTAAAAGTTATAGGAAAAATATATAAAGAAATAAAAACATTAAAAAAATATAAGATATGTT
TACAAATTACTATCAGACAATCTCCTTATCTAATAGAAGAGTCAATTAACTAATTGAGTATCTTTAAATTGAAATGTTAGGAAGTGATTT
AAATATGAAAACTTAAATTATAAAAAATCAATATTAATTTATTTTTAAAAAATAGAAAGGAGTGTATAAGATTTATTTTCAAAGTTTAAA
AACAAGAAAATCAATTTAAATTTCAGAAGGAATAAATGTGGTTATAGAAGTGGATTTATTATCAAAAATAATAATACTAGGAGGTTTTT

>DSM-29690

ATAAAAATATGTTAAATATATCCTCTTATACTTAAATATATAAAAATAAACAAAATGATACACTACATAAAGTGTTCTATCTAATATGAA
GATTTACCAATAAAAAGGTGGACTATGATGAATGCACAGTAGTTCACCTTTTTATATTTCTAATGGTAACAAAATATTTTTTTATATAAA
CCTAGGAGGCGTTATGAATATGACAATATCTTTTTTATCAGAGCATATATTTATAAAGTTAGTAATTTTAACTATATCATTTGATACATT
ATTAGGATGTTTAAGTGCAATAAAAAGTCGTAAATTTAATTCTAGTTTTGGAATAGATGGAGGAATCAGAAAAGTAGCAATGATAGCATG
TATATTTTTTTTATCAGTAGTTGACATTCTTACAAAGTTTAACTTTTTATTTATGTTACCACAAGATTGTATCAATTTTTTAAGACTAAA
ACATCTTGGAATATCTGAATTTTTCTCTATTTTATTTATTTTATATGAAAGTGTAAGTATATTAAAAAATATGTGCTTATGTGGATTACC
AGTACCTAAGAGATTAAAGGAAAAAATAGCAATTTTACTAGATGCAATGACAGATGAAATGAATGCTAAGGATGAAAAGTAAGTAATGGT
AGATATAATAAAGATATTAACAAATAAAAAGTGTTATCCAAATAAGAATAGCTGAAAGTTATCATAATTCATGAAACTAATAATGAAAAC
GAGGGAGCAGATGCCAAGAGACACACAAGTATTAAATACATATAATTTCGAAGCAAGTGTTCATTACTATATAGATGACAAGGTAGTATA
TCAAACATTGGTTCACAAAGATGGTGCATGGTCAGTTGGTAAAATCTATTAAGCTACATTAGTTACAGATATCACAAACTATAATAGTTA
AACATAGAAATATGTGTAAATTGTGATTGAAATTATTCAAAAACACAAAAATACGTGATGAAGGACAAAATGATATAGAAAATAAGTATC
AAACCTTAATAAATGATTTAATTGATAGTTTAAAAGTTATAGGAAAAATATATAAAGAAATAAAAACATTAAAAAAATATAAGATATGTT
TACAAATTACTATCAGACAATCTCCTTATCTAATAGAAGAGTCAATTAACTAATTGAGTATCTTTAAATTGAAATGTTAGGAAGTGATTT
AAATATGAAAACTTAAATTATAAAAAATCAATATTAATTTATTTTTAAAAAATAGAAAGGAGTGTATAAGATTTATTTTCAAAGTTTAAA
AACAAGAAAATCAATTTAAATTTCAGAAGGAATAAATGTGGTTATAGAAGTGGATTTATTATCAAAAATAATAATACTAGGAGGTTTTT

>DSM-29691

ATAAAAATATGTTAAATATATCCTCTTATACTTAAATATATAAAAATAAACAAAATGATACACTACATAAAGTGTTCTATCTAATATGAA
GATTTACCAATAAAAAGGTGGACTATGATGAATGCACAGTAGTTCACCTTTTTATATTTCTAATGGTAACAAAATATTTTTTTATATAAA
CCTAGGAGGCGTTATGAATATGACAATATCTTTTTTATCAGAGCATATATTTATAAAGTTAGTAATTTTAACTATATCATTTGATACATT
ATTAGGATGTTTAAGTGCAATAAAAAGTCGTAAATTTAATTCTAGTTTTGGAATAGATGGAGGAATCAGAAAAGTAGCAATGATAGCATG
TATATTTTTTTTATCAGTAGTTGACATTCTTACAAAGTTTAACTTTTTATTTATGTTACCACAAGATTGTATCAATTTTTTAAGACTAAA
ACATCTTGGAATATCTGAATTTTTCTCTATTTTATTTATTTTATATGAAAGTGTAAGTATATTAAAAAATATGTGCTTATGTGGATTACC
AGTACCTAAGAGATTAAAGGAAAAAATAGCAATTTTACTAGATGCAATGACAGATGAAATGAATGCTAAGGATGAAAAGTAAGTAATGGT
AGATATAATAAAGATATTAACAAATAAAAAGTGTTATCCAAATAAGAATAGCTGAAAGTTATCATAATTCATGAAACTAATAATGAAAAC
GAGGGAGCAGATGCCAAGAGACACACAAGTATTAAATACATATAATTTCGAAGCAAGTGTTCATTACTATATAGATGACAAGGTAGTATA
TCAAACATTGGTTCACAAAGATGGTGCATGGTCAGTTGGTAAAATCTATTAAGCTACATTAGTTACAGATATCACAAACTATAATAGTTA
AACATAGAAATATGTGTAAATTGTGATTGAAATTATTCAAAAACACAAAAATACGTGATGAAGGACAAAATGATATAGAAAATAAGTATC
AAACCTTAATAAATGATTTAATTGATAGTTTAAAAGTTATAGGAAAAATATATAAAGAAATAAAAACATTAAAAAAATATAAGATATGTT
TACAAATTACTATCAGACAATCTCCTTATCTAATAGAAGAGTCAATTAACTAATTGAGTATCTTTAAATTGAAATGTTAGGAAGTGATTT
AAATATGAAAACTTAAATTATAAAAAATCAATATTAATTTATTTTTAAAAAATAGAAAGGAGTGTATAAGATTTATTTTCAAAGTTTAAA
AACAAGAAAATCAATTTAAATTTCAGAAGGAATAAATGTGGTTATAGAAGTGGATTTATTATCAAAAATAATAATACTAGGAGGTTTTT

>DSM-29692

ATAAAAATATGTTAAATATATCCTCTTATACTTAAATATATAAAAATAAACAAAATGATACACTACATAAAGTGTTCTATCTAATATGAA
GATTTACCAATAAAAAGGTGGACTATGATGAATGCACAGTAGTTCACCTTTTTATATTTCTAATGGTAACAAAATATTTTTTTATATAAA
CCTAGGAGGCGTTATGAATATGACAATATCTTTTTTATCAGAGCATATATTTATAAAGTTAGTAATTTTAACTATATCATTTGATACATT
ATTAGGATGTTTAAGTGCAATAAAAAGTCGTAAATTTAATTCTAGTTTTGGAATAGATGGAGGAATCAGAAAAGTAGCAATGATAGCATG
TATATTTTTTTTATCAGTAGTTGACATTCTTACAAAGTTTAACTTTTTATTTATGTTACCACAAGATTGTATCAATTTTTTAAGACTAAA
ACATCTTGGAATATCTGAATTTTTCTCTATTTTATTTATTTTATATGAAAGTGTAAGTATATTAAAAAATATGTGCTTATGTGGATTACC
AGTACCTAAGAGATTAAAGGAAAAAATAGCAATTTTACTAGATGCAATGACAGATGAAATGAATGCTAAGGATGAAAAGTAAGTAATGGT
AGATATAATAAAGATATTAACAAATAAAAAGTGTTATCCAAATAAGAATAGCTGAAAGTTATCATAATTCATGAAACTAATAATGAAAAC
GAGGGAGCAGATGCCAAGAGACACACAAGTATTAAATACATATAATTTCGAAGCAAGTGTTCATTACTATATAGATGACAAGGTAGTATA
TCAAACATTGGTTCACAAAGATGGTGCATGGTCAGTTGGTAAAATCTATTAAGCTACATTAGTTACAGATATCACAAACTATAATAGTTA
AACATAGAAATATGTGTAAATTGTGATGGAAATTATTCAAAAACACAAAAATACGTGATGAAGGACAAAATGATATAGAAAATAAGTATC
AAACCTTAATAAATGATTTAATTGATAGTTTAAAAGTTATAGGAAAAATATATAAAGAAATAAAAACATTAAAAAAATATAAGATATGTT
TACAAATTACTATCAGACAATCTCCTTATCTAATAGAAGAGTCAATTAACTAATTGAGTATCTTTAAATTGAAATGTTAGGAAGTGATTT
AAATATGAAAACTTAAATTATAAAAAATCAATATTAATTTATTTTTAAAAAATAGAAAGGAGTGTATAAGATTTATTTTCAAAGTTTAAA
AACAAGAAAATCAATTTAAATTTCAGAAGGAATAAATGTGGTTATAGAAGTGGATTTATTATCAAAAATAATAATACTAGGAGGTTTTT

>DSM-29091

ATAAAAATATGTTAAATATATCCTCTTATACTTAAATATATAAAAATAAACAAAATGATACACTACATAAAGTGTTCTATCTAATATGAA
GATTTACCAATAAAAAGGTGGACTATGATGAATGCACAGTAGTTCACCTTTTTATATTTCTAATGGTAACAAAATATTTTTTTATATAAA
CCTAGGAGGCGTTATGAATATGACAATATCTTTTTTATCAGAGCATATATTTATAAAGTTAGTAATTTTAACTATATCATTTGATACATT
ATTAGGATGTTTAAGTGCAATAAAAAGTCGTAAATTTAATTCTAGTTTTGGAATAGATGGAGGAATCAGAAAAGTAGCAATGATAGCATG
TATATTTTTTTTATCAGTAGTTGACATTCTTACAAAGTTTAACTTTTTATTTATGTTACCACAAGATTGTATCAATTTTTTAAGACTAAA
ACATCTTGGAATATCTGAATTTTTCTCTATTTTATTTATTTTATATGAAAGTGTAAGTATATTAAAAAATATGTGCTTATGTGGATTACC
AGTACCTAAGAGATTAAAGGAAAAAATAGCAATTTTACTAGATGCAATGACAGATGAAATGAATGCTAAGGATGAAAAGTAAGTAATGGT
AGATATAATAAAGATATTAACAAATAAAAAGTGTTATCCAAATAAGAATAGCTGAAAGTTATCATAATTCATGAAACTAATAATGAAAAC
GAGGGAGCAGATGCCAAGAGACACACAAGTATTAAATACATATAATTTCGAAGCAAGTGTTCATTACTATATAGATGACAAGGTAGTATA
TCAAACATTGGTTCACAAAGATGGTGCATGGTCAGTTGGTAAAATCTATTAAGCTACATTAGTTACAGATATCACAAACTATAATAGTTA
AACATAGAAATATGTGTAAATTGTGATGGAAATTATTCAAAAACACAAAAATACGTGATGAAGGACAAAATGATATAGAAAATAAGTATC
AAACCTTAATAAATGATTTAATTGATAGTTTAAAAGTTATAGGAAAAATATATAAAGAAATAAAAACATTAAAAAAATATAAGATATGTT
TACAAATTACTATCAGACAATCTCCTTATCTAATAGAAGAGTCAATTAACTAATTGAGTATCTTTAAATTGAAATGTTAGGAAGTGATTT
AAATATGAAAACTTAAATTATAAAAAATCAATATTAATTTATTTTTAAAAAATAGAAAGGAGTGTATAAGATTTATTTTCAAAGTTTAAA
AACAAGAAAATCAATTTAAATTTCAGAAGGAATAAATGTGGTTATAGAAGTGGATTTATTATCAAAAATAATAATACTAGGAGGTTTTT

>630

ATAAAAATATGTTAAATATATCCTCTTATACTTAAATATATAAAAATAAACAAAATGATACACTACATAAAGTGTTCTATCTAATATGAA
GATTTACCAATAAAAAGGTGGACTATGATGAATGCACAGTAGTTCACCTTTTTATATTTCTAATGGTAACAAAATATTTTTTTATATAAA
CCTAGGAGGCGTTATGAATATGACAATATCTTTTTTATCAGAGCATATATTTATAAAGTTAGTAATTTTAACTATATCATTTGATACATT
ATTAGGATGTTTAAGTGCAATAAAAAGTCGTAAATTTAATTCTAGTTTTGGAATAGATGGAGGAATCAGAAAAGTAGCAATGATAGCATG
TATATTTTTTTTATCAGTAGTTGACATTCTTACAAAGTTTAACTTTTTATTTATGTTACCACAAGATTGTATCAATTTTTTAAGACTAAA
ACATCTTGGAATATCTGAATTTTTCTCTATTTTATTTATTTTATATGAAAGTGTAAGTATATTAAAAAATATGTGCTTATGTGGATTACC
AGTACCTAAGAGATTAAAGGAAAAAATAGCAATTTTACTAGATGCAATGACAGATGAAATGAATGCTAAGGATGAAAAGTAAGTAATGGT
AGATATAATAAAGATATTAACAAATAAAAAGTGTTATCCAAATAAGAATAGCTGAAAGTTATCATAATTCATGAAACTAATAATGAAAAC
GAGGGAGCAGATGCCAAGAGACACACAAGTATTAAATACATATAATTTCGAAGCAAGTGTTCATTACTATATAGATGACAAGGTAGTATA
TCAAACATTGGTTCACAAAGATGGTGCATGGTCAGTTGGTAAAATCTATTAAGCTACATTAGTTACAGATATCACAAACTATAATAGTTA
AACATAGAAATATGTGTAAATTGTGATGGAAATTATTCAAAAACACAAAAATACGTGATGAAGGACAAAATGATATAGAAAATAAGTATC
AAACCTTAATAAATGATTTAATTGATAGTTTAAAAGTTATAGGAAAAATATATAAAGAAATAAAAACATTAAAAAAATATAAGATATGTT
TACAAATTACTATCAGACAATCTCCTTATCTAATAGAAGAGTCAATTAACTAATTGAGTATCTTTAAATTGAAATGTTAGGAAGTGATTT
AAATATGAAAACTTAAATTATAAAAAATCAATATTAATTTATTTTTAAAAAATAGAAAGGAGTGTATAAGATTTATTTTCAAAGTTTAAA
AACAAGAAAATCAATTTAAATTTCAGAAGGAATAAATGTGGTTATAGAAGTGGATTTATTATCAAAAATAATAATACTAGGAGGTTTTT

>630-delta-erm

ATAAAAATATGTTAAATATATCCTCTTATACTTAAATATATAAAAATAAACAAAATGATACACTACATAAAGTGTTCTATCTAATATGAA
GATTTACCAATAAAAAGGTGGACTATGATGAATGCACAGTAGTTCACCTTTTTATATTTCTAATGGTAACAAAATATTTTTTTATATAAA
CCTAGGAGGCGTTATGAATATGACAATATCTTTTTTATCAGAGCATATATTTATAAAGTTAGTAATTTTAACTATATCATTTGATACATT
ATTAGGATGTTTAAGTGCAATAAAAAGTCGTAAATTTAATTCTAGTTTTGGAATAGATGGAGGAATCAGAAAAGTAGCAATGATAGCATG
TATATTTTTTTTATCAGTAGTTGACATTCTTACAAAGTTTAACTTTTTATTTATGTTACCACAAGATTGTATCAATTTTTTAAGACTAAA
ACATCTTGGAATATCTGAATTTTTCTCTATTTTATTTATTTTATATGAAAGTGTAAGTATATTAAAAAATATGTGCTTATGTGGATTACC
AGTACCTAAGAGATTAAAGGAAAAAATAGCAATTTTACTAGATGCAATGACAGATGAAATGAATGCTAAGGATGAAAAGTAAGTAATGGT
AGATATAATAAAGATATTAACAAATAAAAAGTGTTATCCAAATAAGAATAGCTGAAAGTTATCATAATTCATGAAACTAATAATGAAAAC
GAGGGAGCAGATGCCAAGAGACACACAAGTATTAAATACATATAATTTCGAAGCAAGTGTTCATTACTATATAGATGACAAGGTAGTATA
TCAAACATTGGTTCACAAAGATGGTGCATGGTCAGTTGGTAAAATCTATTAAGCTACATTAGTTACAGATATCACAAACTATAATAGTTA
AACATAGAAATATGTGTAAATTGTGATGGAAATTATTCAAAAACACAAAAATACGTGATGAAGGACAAAATGATATAGAAAATAAGTATC
AAACCTTAATAAATGATTTAATTGATAGTTTAAAAGTTATAGGAAAAATATATAAAGAAATAAAAACATTAAAAAAATATAAGATATGTT
TACAAATTACTATCAGACAATCTCCTTATCTAATAGAAGAGTCAATTAACTAATTGAGTATCTTTAAATTGAAATGTTAGGAAGTGATTT
AAATATGAAAACTTAAATTATAAAAAATCAATATTAATTTATTTTTAAAAAATAGAAAGGAGTGTATAAGATTTATTTTCAAAGTTTAAA
AACAAGAAAATCAATTTAAATTTCAGAAGGAATAAATGTGGTTATAGAAGTGGATTTATTATCAAAAATAATAATACTAGGAGGTTTTT

>DSM-29092

ATAAAAATATGTTAAATATATCCTCTTATACTTAAATATATAAAAATAAACAAAATGATACACTACATAAAGTGTTCTATCTAATATGAA
GATTTACCAATAAAAAGGTGGACTATGATGAATGCACAGTAGTTCACCTTTTTATATTTCTAATGGTAACAAAATATTTTTTTATATAAA
CCTAGGAGGCGTTATGAATATGACAATATCTTTTTTATCAGAGCATATATTTATAAAGTTAGTAATTTTAACTATATCATTTGATACATT
ATTAGGATGTTTAAGTGCAATAAAAAGTCGTAAATTTAATTCTAGTTTTGGAATAGATGGAGGAATCAGAAAAGTAGCAATGATAGCATG
TATATTTTTTTTATCAGTAGTTGACATTCTTACAAAGTTTAACTTTTTATTTATGTTACCACAAGATTGTATCAATTTTTTAAGACTAAA
ACATCTTGGAATATCTGAATTTTTCTCTATTTTATTTATTTTATATGAAAGTGTAAGTATATTAAAAAAATATGTGCTTATGTGGATTACC
AGTACCTAAGAGATTAAAGGAAAAAAATAGCAATTTTACTAGATGCAATGACAGATGAAATGAATGCTAAGGATGAAAAGTAAGTAATGGT
AGATATAATAAAGATATTAACAAATAAAAAGTGTTATCCAAATAAGAATAGCTGAAAGTTATCATAATTCATGAAACTAATAATGAAAAC
GAGGGAGCAGATGCCAAGAGACACACAAGTATTAAATACATATAATTTCGAAGCAAGTGTTCATTACTATATAGATGACAAGGTAGTATA
TCAAACATTGGTTCACAAAGATGGTGCATGGTCAGTTGGTAAAATCTATTAAGCTACATTAGTTACAGATATCACAAACTATAATAGTTA
AACATAGAAATATGTGTAAATTGTGATTGAAATTATTCAAAAACACAAAAATACGTGATGAAGGACAAAATGATATAGAAAATAAGTATC
AAACCTTAATAAATGATTTAATTGATAGTTTAAAAGTTATAGGAAAAATATATAAAGAAATAAAAACATTAAAAAAATATAAGATATGTT
TACAAATTACTATCAGACAATCTCCTTATCTAATAGAAGAGTCAATTAACTAATTGAGTATCTTTAAATTGAAATGTTAGGAAGTGATTT
AAATATGAAAACTTAAATTATAAAAAAATCAATATTAATTTATTTTTAAAAAAATAGAAAGGAGTGTATAAGATTTATTTTCAAAGTTTAAA
AACAAGAAAATCAATTTAAATTTCAGAAGGAATAAATGTGGTTATAGAAGTGGATTTATTATCAAAAATAATAATACTAGGAGGTTTTT

>DSM-29744

ATAAAAATATGTTAAATATATCCTCTTATACTTAAATATATAAAAATAAACAAAATGATACACTACATAAAGTGTTCTATCTAATATGAA
GATTTACCAATAAAAAGGTGGACTATGATGAATGCACAGTAGTTCACCTTTTTATATTTCTAATGGTAACAAAATATTTTTTTATATAAA
CCTAGGAGGCGTTATGAATATGACAATATCTTTTTTATCAGAGCATATATTTATAAAGTTAGTAATTTTAACTATATCATTTGATACATT
ATTAGGATGTTTAAGTGCAATAAAAAGTCGTAAATTTAATTCTAGTTTTGGAATAGATGGAGGAATCAGAAAAGTAGCAATGATAGCATG
TATATTTTTTTTATCAGTAGTTGACATTCTTACAAAGTTTAACTTTTTATTTATGTTACCACAAGATTGTATCAATTTTTTAAGACTAAA
ACATCTTGGAATATCTGAATTTTTCTCTATTTTATTTATTTTATATGAAAGTGTAAGTATATTAAAAAAATATGTGCTTATGTGGATTACC
AGTACCTAAGAGATTAAAGGAAAAAAATAGCAATTTTACTAGATGCAATGACAGATGAAATGAATGCTAAGGATGAAAAGTAAGTAATGGT
AGATATAATAAAGATATTAACAAATAAAAAGTGTTATCCAAATAAGAATAGCTGAAAGTTATCATAATTCATGAAACTAATAATGAAAAC
GAGGGAGCAGATGCCAAGAGACACACAAGTATTAAATACATATAATTTCGAAGCAAGTGTTCATTACTATATAGATGACAAGGTAGTATA
TCAAACATTGGTTCACAAAGATGGTGCATGGTCAGTTGGTAAAATCTATTAAGCTACATTAGTTACAGATATCACAAACTATAATAGTTA
AACATAGAAATATGTGTAAATTGTGATGGAAATTATTCAAAAACACAAAAATACGTGATGAAGGACAAAATGATATAGAAAATAAGTATC
AAACCTTAATAAATGATTTAATTGATAGTTTAAAAGTTATAGGAAAAATATATAAAGAAATAAAAACATTAAAAAAATATAAGATATGTT
TACAAATTACTATCAGACAATCTCCTTATCTAATAGAAGAGTCAATTAACTAATTGAGTATCTTTAAATTGAAATGTTAGGAAGTGATTT
AAATATGAAAACTTAAATTATAAAAAAATCAATATTAATTTATTTTTAAAAAAATAGAAAGGAGTGTATAAGATTTATTTTCAAAGTTTAAA
AACAAGAAAATCAATTTAAATTTCAGAAGGAATAAATGTGGTTATAGAAGTGGATTTATTATCAAAAATAATAATACTAGGAGGTTTTT

>DSM-29745

ATAAAAATATGTTAAATATATCCTCTTATACTTAAATATATAAAAATAAACAAAATGATACACTACATAAAGTGTTCTATCTAATATGAA
GATTTACCAATAAAAAGGTGGACTATGATGAATGCACAGTAGTTCACCTTTTTATATTTCTAATGGTAACAAAATATTTTTTTATATAAA
CCTAGGAGGCGTTATGAATATGACAATATCTTTTTTATCAGAGCATATATTTATAAAGTTAGTAATTTTAACTATATCATTTGATACATT
ATTAGGATGTTTAAGTGCAATAAAAAGTCGTAAATTTAATTCTAGTTTTGGAATAGATGGAGGAATCAGAAAAGTAGCAATGATAGCATG
TATATTTTTTTTATCAGTAGTTGACATTCTTACAAAGTTTAACTTTTTATTTATGTTACCACAAGATTGTATCAATTTTTTAAGACTAAA
ACATCTTGGAATATCTGAATTTTTCTCTATTTTATTTATTTTATATGAAAGTGTAAGTATATTAAAAAAATATGTGCTTATGTGGATTACC
AGTACCTAAGAGATTAAAGGAAAAAAATAGCAATTTTACTAGATGCAATGACAGATGAAATGAATGCTAAGGATGAAAAGTAAGTAATGGT
AGATATAATAAAGATATTAACAAATAAAAAGTGTTATCCAAATAAGAATAGCTGAAAGTTATCATAATTCATGAAACTAATAATGAAAAC
GAGGGAGCAGATGCCAAGAGACACACAAGTATTAAATACATATAATTTCGAAGCAAGTGTTCATTACTATATAGATGACAAGGTAGTATA
TCAAACATTGGTTCACAAAGATGGTGCATGGTCAGTTGGTAAAATCTATTAAGCTACATTAGTTACAGATATCACAAACTATAATAGTTA
AACATAGAAATATGTGTAAATTGTGATGGAAATTATTCAAAAACACAAAAATACGTGATGAAGGACAAAATGATATAGAAAATAAGTATC
AAACCTTAATAAATGATTTAATTGATAGTTTAAAAGTTATAGGAAAAATATATAAAGAAATAAAAACATTAAAAAAATATAAGATATGTT
TACAAATTACTATCAGACAATCTCCTTATCTAATAGAAGAGTCAATTAACTAATTGAGTATCTTTAAATTGAAATGTTAGGAAGTGATTT
AAATATGAAAACTTAAATTATAAAAAAATCAATATTAATTTATTTTTAAAAAAATAGAAAGGAGTGTATAAGATTTATTTTCAAAGTTTAAA
AACAAGAAAATCAATTTAAATTTCAGAAGGAATAAATGTGGTTATAGAAGTGGATTTATTATCAAAAATAATAATACTAGGAGGTTTTT

>DSM-29747

ATAAAAATATGTTAAATATATCCTCTTATACTTAAATATATAAAAATAAACAAAATGATACACTACATAAAGTGTTCTATCTAATATGAA
GATTTACCAATAAAAAGGTGAACTATGATGAATTCACAGTAGTTCACCTTTTTATATTTCTAATGGTAACAAAATATTTTTTTATATAAA
CCTAGGAGGCGTTATGAATATGACAATATCTTTTTTATCAGAGCATATATTTATAAAGTTAGTAATTTTAACTATATCATTTGATACATT
ATTAGGATGTTTAAGTGCAATAAAAAGTCGTAAATTTAATTCTAGTTTTGGAATAGATGGAGGAATCAGAAAAGTAGCAATGATAGCATG
TATATTTTTTTTATCAGTAGTTGACATTCTTACAAAGTTTAACTTTTTATTTATGTTACCACAAGATTGTATCAATTTTTTAAGACTAAA
ACATCTTGGAATATCTGAATTTTTCTCTATTTTATTTATTTTATATGAAAGTGTAAGTATATTAAAAAATATGTGCTTATGTGGATTACC
AGTACCTAAGAGATTAAAGGAAAAAATAGCAGTTTTACTAGATGCAATGACAGATGAAATGAATGCTAAGGATGAAAAGTAAGTACTGGT
AGATATAATAAAGATATTAACAAATAAAAAGTGTTATCCAAATAAGAATAGCTGAAAGTTATCATAATTCATGAAACTGATAATGAAAAC
GAGGGAGCAGATGCCAAGAGACACACAAGTATTAAATACATATAATTTCGAAGCAAGTGTTCATTACTATATGGATGACAAGGTAGTATA
TCAAACATTGGTTCACAAAGATGGTGCATGGTCAGTTGGTAAAATCTATTAAGCTACATTAGTTACAGATATCACAAACTATAATAGTTA
AACATAGAAATATGTGTAAATTGTGATGGAAATTATTCAAAAACACAAAAATACGTGATGAAGGACAAAATGATAGAGAAAATAAGTATC
AAACCTTAATAAATGATTTAATTGATAGTTTAAAAGTTATAGGAAAAATATATAAAGAAATAAAAACATTAAAAAAATATAAGATATGTT
TACAAATTACTATCAGACAATCTCCTTATCTAATAGAAGAGTCAATTAACTAATTGAGTATCTTTAAATTGAAATGTTAGGAAGTGATTT
AAATATGAAAACTTAAATTATAAAAAATCAATATTAATTTATTTTTAAAAAATAGAAAGGAGTGTATAAGATTTATTTTCAAAGTTTAAA
AAAAAGAAAATCAATTTAAATTTCAGAAGGAATAAATGTGGTTATAGAAGTGGATTTATTATCAAAAATAATAATACTAGGAGGTTTTT

>DSM-29093

ATAAAAATATGTTAAATATATCCTCTTATACTTAAATATATAAAAATAAACAAAATGATACACTACATAAAGTGTTCTATCTAATATGAA
GATTTACCAATAAAAAGGTGGACTATGATGAATGCACAGTAGTTCACCTTTTTATATTTCTAATGGTAACAAAATATTTTTTTATATAAA
CCTAGGAGGCGTTATGAATATGACAATATCTTTTTTATCAGAGCATATATTTATAAAGTTAGTAATTTTAACTATATCATTTGATACATT
ATTAGGATGTTTAAGTGCAATAAAAAGTCGTAAATTTAATTCTAGTTTTGGAATAGATGGAGGAATCAGAAAAGTAGCAATGATAGCATG
TATATTTTTTTTATCAGTAGTTGACATTCTTACAAAGTTTAACTTTTTATTTATGTTACCACAAGATTGTATCAATTTTTTAAGACTAAA
ACATCTTGGAATATCTGAATTTTTCTCTATTTTATTTATTTTATATGAAAGTGTAAGTATATTAAAAAATATGTGCTTATGTGGATTACC
AGTACCTAAGAGATTAAAGGAAAAAATAGCAATTTTACTAGATGCAATGACAGATGAAATGAATGCTAAGGATGAAAAGTAAGTAATGGT
AGATATAATAAAGATATTAACAAATAAAAAGTGTTATCCAAATAAGAATAGCTGAAAGTTATCATAATTCATGAAACTAATAATGAAAAC
GAGGGAGCAGATGCCAAGAGACACACAAGTATTAAATACATATAATTTCGAAGCAAGTGTTCATTACTATATAGATGACAAGGTAGTATA
TCAAACATTGGTTCACAAAGATGGTGCATGGTCAGTTGGTAAAATCTATTAAGCTACATTAGTTACAGATATCACAAACTATAATAGTTA
AACATAGAAATATGTGTAAATTGTGATTGAAATTATTCAAAAACACAAAAATACGTGATGAAGGACAAAATGATATAGAAAATAAGTATC
AAACCTTAATAAATGATTTAATTGATAGTTTAAAAGTTATAGGAAAAATATATAAAGAAATAAAAACATTAAAAAAATATAAGATATGTT
TACAAATTACTATCAGACAATCTCCTTATCTAATAGAAGAGTCAATTAACTAATTGAGTATCTTTAAATTGAAATGTTAGGAAGTGATTT
AAATATGAAAACTTAAATTATAAAAAATCAATATTAATTTATTTTTAAAAAATAGAAAGGAGTGTATAAGATTTATTTTCAAAGTTTAAA
AACAAGAAAATCAATTTAAATTTCAGAAGGAATAAATGTGGTTATAGAAGTGGATTTATTATCAAAAATAATAATACTAGGAGGTTTTT

>DSM-29752

ATAAAAATATGTTAAATATATCCTCTTATACTTAAATATATAAAAATAAACAAAATGATACACTACATAAAGTGTTCTATCTAATATGAA
GATTTACCAATAAAAAGGTGAACTATGATGAATTCACAGTAGTTCACCTTTTTATATTTCTAATGGTAACAAAATATTTTTTTATATAAA
CCTAGGAGGCGTTATGAATATGACAATATCTTTTTTATCAGAGCATATATTTATAAAGTTAGTAATTTTAACTATATCATTTGATACATT
ATTAGGATGTTTAAGTGCAATAAAAAGTCGTAAATTTAATTCTAGTTTTGGAATAGATGGAGGAATCAGAAAAGTAGCAATGATAGCATG
TATATTTTTTTTATCAGTAGTTGACATTCTTACAAAGTTTAACTTTTTATTTATGTTACCACAAGATTGTATCAATTTTTTAAGACTAAA
ACATCTTGGAATATCTGAATTTTTCTCTATTTTATTTATTTTATATGAAAGTGTAAGTATATTAAAAAATATGTGCTTATGTGGATTACC
AGTACCTAAGAGATTAAAGGAAAAAATAGCAGTTTTACTAGATGCAATGACAGATGAAATGAATGCTAAGGATGAAAAGTAAGTACTGGT
AGATATAATAAAGATATTAACAAATAAAAAGTGTTATCCAAATAAGAATAGCTGAAAGTTATCATAATTCATGAAACTGATAATGAAAAC
GAGGGAGCAGATGCCAAGAGACACACAAGTATTAAATACATATAATTTCGAAGCAAGTGTTCATTACTATATGGATGACAAGGTAGTATA
TCAAACATTGGTTCACAAAGATGGTGCATGGTCAGTTGGTAAAATCTATTAAGCTACATTAGTTACAGATATCACAAACTATAATAGTTA
AACATAGAAATATGTGTAAATTGTGATGGAAATTATTCAAAAACACAAAAATACGTGATGAAGGACAAAATGATAGAGAAAATAAGTATC
AAACCTTAATAAATGATTTAATTGATAGTTTAAAAGTTATAGGAAAAATATATAAAGAAATAAAAACATTAAAAAAATATAAGATATGTT
TACAAATTACTATCAGACAATCTCCTTATCTAATAGAAGAGTCAATTAACTAATTGAGTATCTTTAAATTGAAATGTTAGGAAGTGATTT
AAATATGAAAACTTAAATTATAAAAAATCAATATTAATTTATTTTTAAAAAATAGAAAGGAGTGTATAAGATTTATTTTCAAAGTTTAAA
AAAAAGAAAATCAATTTAAATTTCAGAAGGAATAAATGTGGTTATAGAAGTGGATTTATTATCAAAAATAATAATACTAGGAGGTTTTT

>DSM-29753

ATAAAAATATGTTAAATATATCCTCTTATACTTAAATATATAAAAATAAACAAAATGATACACTACATAAAGTGTTCTATCTAATATGAA
GATTTACCAATAAAAAGGTGAACTATGATGAATTCACAGTAGTTCACCTTTTTATATTTCTAATGGTAACAAAATATTTTTTTATATAAA
CCTAGGAGGCGTTATGAATATGACAATATCTTTTTTATCAGAGCATATATTTATAAAGTTAGTAATTTTAACTATATCATTTGATACATT
ATTAGGATGTTTAAGTGCAATAAAAAGTCGTAAATTTAATTCTAGTTTTGGAATAGATGGAGGAATCAGAAAAGTAGCAATGATAGCATG
TATATTTTTTTTATCAGTAGTTGACATTCTTACAAAGTTTAACTTTTTATTTATGTTACCACAAGATTGTATCAATTTTTTAAGACTAAA
ACATCTTGGAATATCTGAATTTTTCTCTATTTTATTTATTTTATATGAAAGTGTAAGTATATTAAAAAATATGTGCTTATGTGGATTACC
AGTACCTAAGAGATTAAAGGAAAAAATAGCAGTTTTACTAGATGCAATGACAGATGAAATGAATGCTAAGGATGAAAAGTAAGTACTGGT
AGATATAATAAAGATATTAACAAATAAAAAGTGTTATCCAAATAAGAATAGCTGAAAGTTATCATAATTCATGAAACTGATAATGAAAAC
GAGGGAGCAGATGCCAAGAGACACACAAGTATTAAATACATATAATTTCGAAGCAAGTGTTCATTACTATATGGATGACAAGGTAGTATA
TCAAACATTGGTTCACAAAGATGGTGCATGGTCAGTTGGTAAAATCTATTAAGCTACATTAGTTACAGATATCACAAACTATAATAGTTA
AACATAGAAATATGTGTAAATTGTGATGGAAATTATTCAAAAACACAAAAATACGTGATGAAGGACAAAATGATAGAGAAAATAAGTATC
AAACCTTAATAAATGATTTAATTGATAGTTTAAAAGTTATAGGAAAAATATATAAAGAAATAAAAACATTAAAAAAATATAAGATATGTT
TACAAATTACTATCAGACAATCTCCTTATCTAATAGAAGAGTCAATTAACTAATTGAGTATCTTTAAATTGAAATGTTAGGAAGTGATTT
AAATATGAAAACTTAAATTATAAAAAATCAATATTAATTTATTTTTAAAAAATAGAAAGGAGTGTATAAGATTTATTTTCAAAGTTTAAA
AAAAAGAAAATCAATTTAAATTTCAGAAGGAATAAATGTGGTTATAGAAGTGGATTTATTATCAAAAATAATAATACTAGGAGGTTTTT

>DSM-29754

ATAAAAATATGTTAAATATATCCTCTTATACTTAAATATATAAAAATAAACAAAATGATACACTACATAAAGTGTTCTATCTAATATGAA
GATTTACCAATAAAAAGGTGGACTATGATGAATGCACAGTAGTTCACCTTTTTATATTTCTAATGGTAACAAAATATTTTTTTATATAAA
CCTAGGAGGCGTTATGAATATGACAATATCTTTTTTATCAGAGCATATATTTATAAAGTTAGTAATTTTAACTATATCATTTGATACATT
ATTAGGATGTTTAAGTGCAATAAAAAGTCGTAAATTTAATTCTAGTTTTGGAATAGATGGAGGAATCAGAAAAGTAGCAATGATAGCATG
TATATTTTTTTTATCAGTAGTTGACATTCTTACAAAGTTTAACTTTTTATTTATGTTACCACAAGATTGTATCAATTTTTTAAGACTAAA
ACATCTTGGAATATCTGAATTTTTCTCTATTTTATTTATTTTATATGAAAGTGTAAGTATATTAAAAAATATGTGCTTATGTGGATTACC
AGTACCTAAGAGATTAAAGGAAAAAATAGCAATTTTACTAGATGCAATGACAGATGAAATGAATGCTAAGGATGAAAAGTAAGTAATGGT
AGATATAATAAAGATATTAACAAATAAAAAGTGTTATCCAAATAAGAATAGCTGAAAGTTATCATAATTCATGAAACTAATAATGAAAAC
GAGGGAGCAGATGCCAAGAGACACACAAGTATTAAATACATATAATTTCGAAGCAAGTGTTCATTACTATATAGATGACAAGGTAGTATA
TCAAACATTGGTTCACAAAGATGGTGCATGGTCAGTTGGTAAAATCTATTAAGCTACATTAGTTACAGATATCACAAACTATAATAGTTA
AACATAGAAATATGTGTAAATTGTGATGGAAATTATTCAAAAACACAAAAATACGTGATGAAGGACAAAATGATATAGAAAATAAGTATC
AAACCTTAATAAATGATTTAATTGATAGTTTAAAAGTTATAGGAAAAATATATAAAGAAATAAAAACATTAAAAAAATATAAGATATGTT
TACAAATTACTATCAGACAATCTCCTTATCTAATAGAAGAGTCAATTAACTAATTGAGTATCTTTAAATTGAAATGTTAGGAAGTGATTT
AAATATGAAAACTTAAATTATAAAAAATCAATATTAATTTATTTTTAAAAAATAGAAAGGAGTGTATAAGATTTATTTTCAAAGTTTAAA
AACAAGAAAATCAATTTAAATTTCAGAAGGAATAAATGTGGTTATAGAAGTGGATTTATTATCAAAAATAATAATACTAGGAGGTTTTT

>DSM-29756

ATAAAAATATGTTAAATATATCCTCTTATACTTAAATATATAAAAATAAACAAAATGATACACTACATAAAGTGTTCTATCTAATATGAA
GATTTACCAATAAAAAGGTGGACTATGATGAATGCACAGTAGTTCACCTTTTTATATTTCTAATGGTAACAAAATATTTTTTTATATAAA
CCTAGGAGGCGTTATGAATATGACAATATCTTTTTTATCAGAGCATATATTTATAAAGTTAGTAATTTTAACTATATCATTTGATACATT
ATTAGGATGTTTAAGTGCAATAAAAAGTCGTAAATTTAATTCTAGTTTTGGAATAGATGGAGGAATCAGAAAAGTAGCAATGATAGCATG
TATATTTTTTTTATCAGTAGTTGACATTCTTACAAAGTTTAACTTTTTATTTATGTTACCACAAGATTGTATCAATTTTTTAAGACTAAA
ACATCTTGGAATATCTGAATTTTTCTCTATTTTATTTATTTTATATGAAAGTGTAAGTATATTAAAAAATATGTGCTTATGTGGATTACC
AGTACCTAAGAGATTAAAGGAAAAAATAGCAATTTTACTAGATGCAATGACAGATGAAATGAATGCTAAGGATGAAAAGTAAGTAATGGT
AGATATAATAAAGATATTAACAAATAAAAAGTGTTATCCAAATAAGAATAGCTGAAAGTTATCATAATTCATGAAACTAATAATGAAAAC
GAGGGAGCAGATGCCAAGAGACACACAAGTATTAAATACATATAATTTCGAAGCAAGTGTTCATTACTATATAGATGACAAGGTAGTATA
TCAAACATTGGTTCACAAAGATGGTGCATGGTCAGTTGGTAAAATCTATTAAGCTACATTAGTTACAGATATCACAAACTATAATAGTTA
AACATAGAAATATGTGTAAATTGTGATTGAAATTATTCAAAAACACAAAAATACGTGATGAAGGACAAAATGATATAGAAAATAAGTATC
AAACCTTAATAAATGATTTAATTGATAGTTTAAAAGTTATAGGAAAAATATATAAAGAAATAAAAACATTAAAAAAATATAAGATATGTT
TACAAATTACTATCAGACAATCTCCTTATCTAATAGAAGAGTCAATTAACTAATTGAGTATCTTTAAATTGAAATGTTAGGAAGTGATTT
AAATATGAAAACTTAAATTATAAAAAATCAATATTAATTTATTTTTAAAAAATAGAAAGGAGTGTATAAGATTTATTTTCAAAGTTTAAA
AACAAGAAAATCAATTTAAATTTCAGAAGGAATAAATGTGGTTATAGAAGTGGATTTATTATCAAAAATAATAATACTAGGAGGTTTTT

>DSM-29094

ATAAAAATATGTTAAATATATCCTCTTATACTTAAATATATAAAAATAAACAAAATGATACACTACATAAAGTGTTCTATCTAATATGAA
GATTTACCAATAAAAAGGTGGACTATGATGAATGCACAGTAGTTCACCTTTTTATATTTCTAATGGTAACAAAATATTTTTTTATATAAA
CCTAGGAGGCGTTATGAATATGACAATATCTTTTTTATCAGAGCATATATTTATAAAGTTAGTAATTTTAACTATATCATTTGATACATT
ATTAGGATGTTTAAGTGCAATAAAAAGTCGTAAATTTAATTCTAGTTTTGGAATAGATGGAGGAATCAGAAAAGTAGCAATGATAGCATG
TATATTTTTTTTATCAGTAGTTGACATTCTTACAAAGTTTAACTTTTTATTTATGTTACCACAAGATTGTATCAATTTTTTAAGACTAAA
ACATCTTGGAATATCTGAATTTTTCTCTATTTTATTTATTTTATATGAAAGTGTAAGTATATTAAAAAATATGTGCTTATGTGGATTACC
AGTACCTAAGAGATTAAAGGAAAAAATAGCAATTTTACTAGATGCAATGACAGATGAAATGAATGCTAAGGATGAAAAGTAAGTAATGGT
AGATATAATAAAGATATTAACAAATAAAAAGTGTTATCCAAATAAGAATAGCTGAAAGTTATCATAATTCATGAAACTAATAATGAAAAC
GAGGGAGCAGATGCCAAGAGACACACAAGTATTAAATACATATAATTTCGAAGCAAGTGTTCATTACTATATAGATGACAAGGTAGTATA
TCAAACATTGGTTCACAAAGATGGTGCATGGTCAGTTGGTAAAATCTATTAAGCTACATTAGTTACAGATATCACAAACTATAATAGTTA
AACATAGAAATATGTGTAAATTGTGATTGAAATTATTCAAAAACACAAAAATACGTGATGAAGGACAAAATGATATAGAAAATAAGTATC
AAACCTTAATAAATGATTTAATTGATAGTTTAAAAGTTATAGGAAAAATATATAAAGAAATAAAAACATTAAAAAAATATAAGATATGTT
TACAAATTACTATCAGACAATCTCCTTATCTAATAGAAGAGTCAATTAACTAATTGAGTATCTTTAAATTGAAATGTTAGGAAGTGATTT
AAATATGAAAACTTAAATTATAAAAAATCAATATTAATTTATTTTTAAAAAATAGAAAGGAGTGTATAAGATTTATTTTCAAAGTTTAAA
AACAAGAAAATCAATTTAAATTTCAGAAGGAATAAATGTGGTTATAGAAGTGGATTTATTATCAAAAATAATAATACTAGGAGGTTTTT

>M120

ATAAAAATATGTTAAATATATCCTCTTATACTTAAATATATAAAAATAAACAAAATGATACACTACATAAAGTGTTCTATCTAATATGAA
GATTTACCAATAAAAAGGTGAACTATGATGAATTCACAGTAGTTCACCTTTTTATATTTCTAATGGTAACAAAATATTTTTTTATATAAA
CCTAGGAGGCGTTATGAATATGACAATATCTTTTTTATCAGAGCATATATTTATAAAGTTAGTAATTTTAACTATATCATTTGATACATT
ATTAGGATGTTTAAGTGCAATAAAAAGTCGTAAATTTAATTCTAGTTTTGGAATAGATGGAGGAATCAGAAAAGTAGCAATGATAGCATG
TATATTTTTTTTATCAGTAGTTGACATTCTTACAAAGTTTAACTTTTTATTTATGTTACCACAAGATTGTATCAATTTTTTAAGACTAAA
ACATCTTGGAATATCTGAATTTTTCTCTATTTTATTTATTTTATATGAAAGTGTAAGTATATTAAAAAATATGTGCTTATGTGGATTACC
AGTACCTAAGAGATTAAAGGAAAAAATAGCAGTTTTACTAGATGCAATGACAGATGAAATGAATGCTAAGGATGAAAAGTAAGTACTGGT
AGATATAATAAAGATATTAACAAATAAAAAGTGTTATCCAAATAAGAATAGCTGAAAGTTATCATAATTCATGAAACTGATAATGAAAAC
GAGGGAGCAGATGCCAAGAGACACACAAGTATTAAATACATATAATTTCGAAGCAAGTGTTCATTACTATATGGATGACAAGGTAGTATA
TCAAACATTGGTTCACAAAGATGGTGCATGGTCAGTTGGTAAAATCTATTAAGCTACATTAGTTACAGATATCACAAACTATAATAGTTA
AACATAGAAATATGTGTAAATTGTGATGGAAATTATTCAAAAACACAAAAATACGTGATGAAGGACAAAATGATAGAGAAAATAAGTATC
AAACCTTAATAAATGATTTAATTGATAGTTTAAAAGTTATAGGAAAAATATATAAAGAAATAAAAACATTAAAAAAATATAAGATATGTT
TACAAATTACTATCAGACAATCTCCTTATCTAATAGAAGAGTCAATTAACTAATTGAGTATCTTTAAATTGAAATGTTAGGAAGTGATTT
AAATATGAAAACTTAAATTATAAAAAATCAATATTAATTTATTTTTAAAAAATAGAAAGGAGTGTATAAGATTTATTTTCAAAGTTTAAA
AAAAAGAAAATCAATTTAAATTTCAGAAGGAATAAATGTGGTTATAGAAGTGGATTTATTATCAAAAATAATAATACTAGGAGGTTTTT

>R20291

ATAAAAATACGTTAAATATATCCTCTTATACTTAAATATATAAAAATAAACAAAATGATACACTACATAAAGTGTTCTATCTAATATGAA
GATTTACCAATAAAAAGGTGAACTATGATGAATGCACAGTAGTTCACCTTTTTATATTTCTAATGGTAACAAAATATTTTTTTATATAAA
CCTAGGAGGCGTTATGAATATGACAATATCTTTTTTATCAGAGCATATATTTATAAAGTTAGTAATTTTAACTATATCATTTGATACATT
ATTAGGATGTTTAAGTGCAATAAAAAGTCGTAAATTTAATTCTAGTTTTGGAATAGATGGAGGAATCAGAAAAGTAGCAATGATAGCATG
TATATTTTTTTTATCAGTAGTTGACATTCTTACAAAGTTTAACTTTTTATTTATGTTACCACAAGATTGTATCAATTTTTTAAGACTAAA
ACATCTTGGAATATCTGAATTTTTCTCTATTTTATTTATTTTATATGAAAGTGTAAGCATATTAAAAAATATGTGCTTATGTGGATTACC
AGTACCTAAGAGATTAAAGGAAAAAATAGCAGTTTTACTAGATGCAATGACAGATGAAATGAATGCTAAGGATGAAAAGTAAGTAATGGT
AGATATAATAAAAATATTAACAAATAAAAAGTGTTATCCAAATAAGAATAGCTGAAAGTTATCATAATTCATGAAACTGATAATGAAAAC
GAGGGAGCAGATGCCAAGAGACACACAAGTATTAAATACATATAATTTCGAAGCAAGTGTTCATTACTATATGGATGACAAGGTAGTATA
TCAAACATTGGTTCACAAAGATGGTGCATGGTCAGTTGGTAAAATCTATTAAGCTACATTAGTTACAGATATCACAAACTATAATAGTTA
AACATAGAAATATGTGTAAATTGTGATGGAAATTATTCAAAAACACAAAAATACGTGATGAAGGACAAAATGATAGAGAAAATAAGTATC
AAACCTTAATAAATGATTTAATTGATAGTTTAAAAGTTATAGGAAAAATATATAAAGAAATAAAAACATTAAAAAAATATAAGATATGTT
TACAAATTACTATCAGACAATCTCCTTATCTAATAGAAGAGTCAATTAACTAATTGAGTATCTTTAAATTGAAATGTTAGGAAGTGATTT
AAATATGAAAACTTAAATTATAAAAAATCAATATTAATTTATCTTTAAAAAATAGAAAGGAGTGTATAAGATTTATTTTCAAAGTTTAAA
AACAAGAAAATCAATTTAAATTTCAGAAGGAATAAATGTGGTTATAGAAGTGGATTTATTATCAAAAATAATAGTACTAGGAGGTTTTT

```
>M68

ATAAAAATATGTTAAATATATCCTCTTATACTTAAATATATAAAAATAAACAAAATGATACACTACATAAAGTGTTCTATCTAATATGAA
GATTTACCAATAAAAAGGTGGACTATGATGAATGCACAGTAGTTCACCTTTTTATATTTCTAATGGTAACAAAATATTTTTTTATATAAA
CCTAGGAGGCGTTATGAATATGACAATATCTTTTTTATCAGAGCATATATTTATAAAGTTAGTAATTTTAACTATATCATTTGATACATT
ATTAGGATGTTTAAGTGCAATAAAAAGTCGTAAATTTAATTCTAGTTTTGGAATAGATGGAGGAATCAGAAAAGTAGCAATGATAGCATG
TATATTTTTTTTATCAGTAGTTGACATTCTTACAAAGTTTAACTTTTTATTTATGTTACCACAAGATTGTATCAATTTTTTAAGACTAAA
ACATCTTGGAATATCTGAATTTTTCTCTATTTTATTTATTTTATATGAAAGTGTAAGTATATTAAAAAATATGTGCTTATGTGGATTACC
AGTACCTAAGAGATTAAAGGAAAAAATAGCAGTTTTACTAGATGCAATGACAGATGAAATGAATGCTAAGGATGAAAAGTAAGTAATGGT
AGATATAATAAAGATATTAACAAATAAAAAGTGTTATCCAAATAAGAATAGCTGAAAGTTATCATAATTCATGAAACTAATAATGAAAAC
GAGGGAGCAGATGCCAAGAGACACACAAGTATTAAATACATATAATTTCGAAGCAAGTGTTCATTACTATATGGATGACAAGGTAGTATA
TCAAACATTGGTTCACAAAGATGGTGCATGGTCAGTTGGTAAAATCTATTAAGCTACATTAGTTACAGATATCACAAACTATAATAGTTA
AACATAGAAATATGTGTAAATTGTGATGGAAATTATTCAAAAACACAAAAATACGTGATGAAGGACAAAATGATAGAGAAAATAAGTATC
AAACCTTAATAAATGATTTAATTGATAGTTTAAAAGTTATAGGAAAAATATATAAAGAAATAAAAACATTAAAAAAATATAAGATATGTT
CTATCATACAATCTCCTTATCTAATAGAAGAGTCAATTAACTAATTGAGTATCTTTAAATTGAAATGTTAGGAAGTGATTTAAATATGAA
AACTTAAATTATAAAAAATCAATATTAATTTATTTTAAAAAAATAGAAAGGAGTGTATAAGATTTATTTTCAAAGTTTAAAAACAAGAAA
ATCAATTTAAATTTCAGAAGGAATAAATGTGGTTATAGAAGTGGATTTATTATCAAAAATAATAATACTAGGAGGTTTTT

>CF5

ATAAAAATATGTTAAATATATCCTCTTATACTTAAATATATAAAAATAAACAAAATGATACACTACATAAAGTGTTCTATCTAATATGAA
GATTTACCAATAAAAAGGTGGACTATGATGAATGCACAGTAGTTCACCTTTTTATATTTCTAATGGTAACAAAATATTTTTTTATATAAA
CCTAGGAGGCGTTATGAATATGACAATATCTTTTTTATCAGAGCATATATTTATAAAGTTAGTAATTTTAACTATATCATTTGATACATT
ATTAGGATGTTTAAGTGCAATAAAAAGTCGTAAATTTAATTCTAGTTTTGGAATAGATGGAGGAATCAGAAAAGTAGCAATGATAGCATG
TATATTTTTTTTATCAGTAGTTGACATTCTTACAAAGTTTAACTTTTTATTTATGTTACCACAAGATTGTATCAATTTTTTAAGACTAAA
ACATCTTGGAATATCTGAATTTTTCTCTATTTTATTTATTTTATATGAAAGTGTAAGTATATTAAAAAATATGTGCTTATGTGGATTACC
AGTACCTAAGAGATTAAAGGAAAAAATAGCAGTTTTACTAGATGCAATGACAGATGAAATGAATGCTAAGGATGAAAAGTAAGTAATGGT
AGATATAATAAAGATATTAACAAATAAAAAGTGTTATCCAAATAAGAATAGCTGAAAGTTATCATAATTCATGAAACTAATAATGAAAAC
GAGGGAGCAGATGCCAAGAGACACACAAGTATTAAATACATATAATTTCGAAGCAAGTGTTCATTACTATATGGATGACAAGGTAGTATA
TCAAACATTGGTTCACAAAGATGGTGCATGGTCAGTTGGTAAAATCTATTAAGCTACATTAGTTACAGATATCACAAACTATAATAGTTA
AACATAGAAATATGTGTAAATTGTGATGGAAATTATTCAAAAACACAAAAATACGTGATGAAGGACAAAATGATAGAGAAAATAAGTATC
AAACCTTAATAATTGATTTAATTGATAGTTTAAAAGTTATAGGAAAAATATATAAAGAAATAAAAACATTAAAAAAATATAAGATATGTT
CTATCATACAATCTCCTTATCTAATAGAAGAGTCAATTAACTAATTGAGTATCTTTAAATTGAAATGTTAGGAAGTGATTTAAATATGAA
AACTTAAATTATAAAAAATCAATATTAATTTATTTTAAAAAAATAGAAAGGAGTGTATAAGATTTATTTTCAAAGTTTAAAAACAAGAAA
ATCAATTTAAATTTCAGAAGGAATAAATGTGGTTATAGAAGTGGATTTATTATCAAAAATAATAATACTAGGAGGTTTTT
```

3 REFERENCES

Reuter, J. S., and Mathews, D. H. (2010). RNAstructure: Software for RNA secondary structure prediction and analysis. *BMC bioinformatics* 11, 129. doi: 10.1186/1471-2105-11-129

Sievers, F., and Higgins, D. G. (2014). Clustal Omega, accurate alignment of very large numbers of sequences. *Methods Mol. Biol.* 1079, 105–116. doi: 10.1007/978-1-62703-646-7_6
